# Improved transcription and translation with L-leucine stimulation of mTORC1 in Roberts syndrome

**DOI:** 10.1186/s12864-015-2354-y

**Published:** 2016-01-05

**Authors:** Baoshan Xu, Madelaine Gogol, Karin Gaudenz, Jennifer L. Gerton

**Affiliations:** Stowers Institute for Medical Research, 1000 E 50th St, Kansas City, MO 64110 USA; Department of Biochemistry and Molecular Biology, University of Kansas School of Medicine, 3901 Rainbow Blvd, Kansas City, KS 66160 USA

**Keywords:** Ribosome profiling, Gene expression, Roberts syndrome (RBS), mTORC1 pathway, Translation, Cohesin

## Abstract

**Background:**

Roberts syndrome (RBS) is a human developmental disorder caused by mutations in the cohesin acetyltransferase ESCO2. We previously reported that mTORC1 signaling was depressed and overall translation was reduced in RBS cells and zebrafish models for RBS. Treatment of RBS cells and zebrafish RBS models with L-leucine partially rescued mTOR function and protein synthesis, correlating with increased cell division and improved development.

**Results:**

In this study, we use RBS cells to model mTORC1 repression and analyze transcription and translation with ribosome profiling to determine gene-level effects of L-leucine. L-leucine treatment partially rescued translational efficiency of ribosomal subunits, translation initiation factors, snoRNA production, and mitochondrial function in RBS cells, consistent with these processes being mTORC1 controlled. In contrast, other genes are differentially expressed independent of L-leucine treatment, including imprinted genes such as *H19* and *GTL2*, miRNAs regulated by GTL2, *HOX* genes, and genes in nucleolar associated domains.

**Conclusions:**

Our study distinguishes between gene expression changes in RBS cells that are TOR dependent and those that are independent. Some of the TOR independent gene expression changes likely reflect the architectural role of cohesin in chromatin looping and gene expression. This study reveals the dramatic rescue effects of L-leucine stimulation of mTORC1 in RBS cells and supports that normal gene expression and translation requires *ESCO2* function.

**Electronic supplementary material:**

The online version of this article (doi:10.1186/s12864-015-2354-y) contains supplementary material, which is available to authorized users.

## Background

Cohesinopathies are a group of developmental disorders, including Roberts syndrome (RBS) and Cornelia de Lange syndrome (CdLS), caused by loss of function mutations in the cohesin complex or its regulators. The cohesin complex is a structural component of chromosomes and helps to facilitate many different chromosomal processes such as genome organization, chromosome segregation, double-strand break repair, and gene expression. The developmental defects associated with the cohesinopathies include slow growth and small size, hirsutism, mental retardation, craniofacial anomalies, limb malformations, and heart, gastrointestinal, and auditory problems. While the molecular etiology of these developmental disorders is unclear, one working model is that the loss of cohesin function results in changes in gene expression during embryogenesis [1–5]. These changes in gene expression could occur via several mechanisms including altered gene looping or genome architecture [6–10].

RBS is an autosomal recessive, multi-system developmental syndrome caused by loss of function mutation in a gene that encodes a cohesin acetyltransferase, *ESCO2* [11]. A hallmark of chromosomes from RBS cells is heterochromatic repulsion, observed in metaphase spreads, possibly indicating a lack of cohesion at these regions [12]. Two genes, *ESCO1* and *ESCO2*, both encode acetyltransferases that acetylate the SMC3 subunit of cohesin during DNA replication to lock the cohesin ring onto DNA. A mutation in yeast *ECO1*, which is a yeast homolog of human *ESCO1/2*, was recently shown to disrupt cohesion, replication, transcription, and looping at the ribosomal DNA (rDNA) repeats [13, 14]. Mutations that disrupt the acetyltransferase activity of Eco1/ESCO2 also disrupt nucleolar architecture, impair ribosomal RNA (rRNA) production and ribosome biogenesis, and reduce protein biosynthesis in budding yeast and human RBS cells [14–16]. Cohesin binds to the rDNA in every organism studied [17], giving cohesin the potential to affect the structure and function of the nucleolus which is essential for both ribosome biogenesis and organization of the genome.

We recently reported that mTOR (mammalian target of rapamycin) signaling was strongly downregulated in human RBS patient cells, accompanied by p53 activation [16]. Amino acids, and in particular L-leucine (L-Leu), have been shown to stimulate mTORC1. In zebrafish models for RBS and CdLS, L-Leu boosted cell proliferation, protein synthesis, and development [16, 18]. The dramatic rescue effect of L-Leu at the cellular and organismal level suggests that cohesinopathies are caused in part by translational defects [19]. Since the mTOR pathway is a critical regulator of protein translation and ribosome function, and p53 is an indicator of nucleolar stress, translational impairment may contribute to differential gene expression in RBS. Therefore, we have used RBS as a disease model to address mTOR-associated transcription and translation dysfunction. A number of reports have shown that mTOR kinase signaling controls mRNA translation by two branches. The first is through phosphorylation of 4EBP1 (Eukaryotic translation initiation factor 4E-binding protein 1) [20, 21]. The unphosphorylated form of 4EBP1 is a translational repressor that directly binds to eIF4E (eukaryotic translation initiation factor 4E), a limiting component for translation initiation for 40S. Phosphorylation of 4EBP1 releases eIF4E for translation initiation. mTOR also controls translation via phosphorylation of RPS6 by RPS6 kinase (S6K1) which activates ribosomal protein S6 to promote its 40S ribosome function. The depression of mTOR observed in RBS cells affects both branches of the pathway which converge on 40S function.

In addition to its role in promoting nucleolar function, cohesin plays a role throughout the genome in forming chromatin loops that can affect gene expression. For instance, cohesin promotes the formation of loops at the imprinted loci *IGF2-H19* [22, 23], Myc [24, 25], and pluripotency factors [6]. The misregulation of any of these master regulators can have grave consequences for cell-type specification and cellular function. The cohesin-dependent control of chromosome organization is another mechanism, in addition to changes in mTOR signaling, that is predicted to underpin the gene expression changes associated with the cohesinopathies.

The rescue provided by L-Leu in animal and tissue culture models for the cohesinopathies strongly suggested that many of the critical transcriptional changes were ameliorated by boosting translation. To address L-Leu dependent transcription and translation at a gene-by-gene level, we examined translation initiation complexes and performed ribosome profiling in RBS cells. We found that L-Leu partially rescued translation initiation, translational efficiency of ribosomal subunits and translation factors, and mitochondrial function in RBS cells. However, other differentially expressed genes do not respond to L-Leu, suggesting they are misexpressed independent of the TOR pathway. These include the imprinted genes and *HOX* genes which are known to be regulated by cohesin-dependent looping events. This is consistent with our previous observation that L-Leu stimulates mTORC1 without rescuing the architectural defects in the nucleolus observed in RBS cells. Our results suggest targeting mTORC1 with L-Leu rescues a significant fraction of the differential gene expression associated with RBS. L-Leu could be a promising therapeutic strategy for human diseases associated with poor translation.

## Results

### 40S and 60S ribosome subunits are present at lower levels in RBS cells

Our previous studies demonstrated ribosome biogenesis and protein synthesis were defective in RBS cells. For our analysis, we used normal human fibroblasts, RBS fibroblasts (homozygous mutation 877_878 delAG in exon 4 of *ESCO2*), and RBS fibroblasts in which a wild-type copy of the *ESCO2* gene has been added back (corrected cells) [12]. We also used two other RBS cell lines (GM21873 and GM21872), which were (1) untransformed amniotic fluid-derived, and (2) a fetal skin fibroblast cell line. For untransformed primary fibroblasts, the donor subject was homozygous for a 5 bp deletion at nucleotide 307 in exon 3 of the *ESCO2* gene (c.307_311delAGAAA) resulting in a frameshift that leads to a truncated protein (p.I102fsX1). For untransformed amniocytes, the donor subject was a compound heterozygote. One allele has a 1 bp deletion at nucleotide 752 in exon 3 of the *ESCO2* gene (c.752delA), and the second allele has an A > G substitution in intron 6 [c.IVS6-7A > G (c.1132-7A > G)]. Both the immortalized RBS fibroblasts and the two untransformed RBS cell lines had similar depression of the mTORC1 signaling pathway, an aberrant cell cycle pattern, and reduced protein translation. Moreover, L-Leu treatment partially rescued cell proliferation and survival, ribosome biogenesis, and protein biosynthesis similarly in all three RBS lines [16]. We selected the transformed RBS fibroblasts for use in our current study because the corrected version provides an excellent control.

**Fig. 1:**
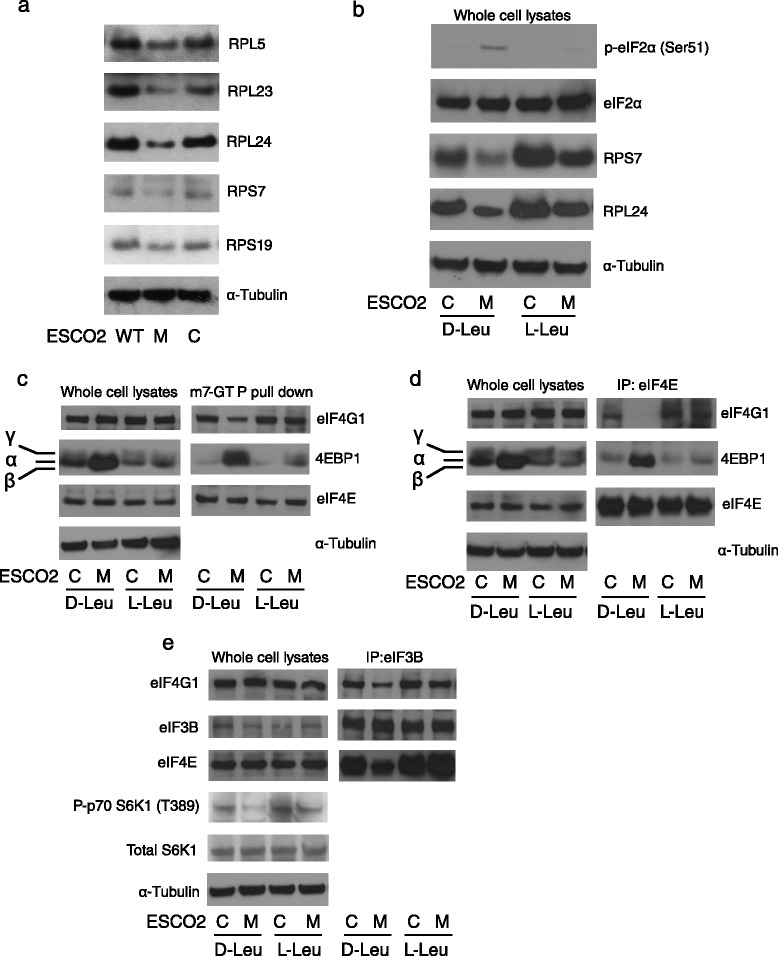
Ribosomal components and translation initiation complexes were present at low levels in RBS cells. **a** Western blotting showed that 40S small ribosome proteins RPS7 and RPS19, and 60S large ribosome proteins RPL5, RPL23, and RPL24 were decreased in *ESCO2* mutant (M) compared to WT fibroblasts (WT) or corrected fibroblasts (C). **b** L-Leu supplement, but not D-Leu, partially rescued RPS7 and RPL24 protein levels, and reversed the elevation of eIF2α phosphorylation in RBS cells. α-Tubulin and eiF2α serve as loading controls. **c** m7-GTP coupled beads were used to pull down translation initiation complexes from whole cell lysates. 4EBP1 protein was strongly enriched in RBS cells, accompanied by less binding of eIF4G1, but this trend was partially reversed in RBS cells treated with L-Leu. eIF4E levels were not affected. **d** Antibodies to eIF4E were used to pull down translation initiation complexes. 4EBP1 was present at high levels in RBS cells, correlating with less eIF4G1 and the inhibition of translation initiation. L-Leu supplement promoted the assembly of the translation competent eIF4E complex. **e** Antibodies to eIF3B were used to pull down translation initiation complexes. eIF4E and eIF4G1 were present at lower levels in RBS cells, but this trend was partially reversed by L-Leu supplement

We decided to examine expression of individual ribosomal proteins in the WT, RBS mutant, and corrected cell lines. Western blotting analysis revealed lower levels of both 40S small subunit and 60S large subunit ribosome proteins including RPS7, RPS19, RPL5, RPL23, and RPL24 in the mutant relative to WT and corrected cells (Fig. 1a, Additional file 1: Figure S1a). Since L-Leu is able to improve protein biosynthesis in RBS cells, we examined the effect of L-Leu on ribosomal proteins. Since D-leucine (D-Leu) is not used as an amino acid, we used D-Leu treatment as a negative control. The levels of both RPS7 and RPL24 were partially rescued by L-Leu supplement but not the bioinactive D-Leu (Fig. 1b, Additional file 1: Figure S1b). In addition, eIF2α phosphorylation was elevated in RBS cells, suggesting a state of translational repression similar to nutrition starvation. Interestingly, we found the phospho-eIF2α level in RBS cells declined with L-Leu supplementation. The data suggested that defective ribosome biogenesis in RBS includes lower levels of ribosomal proteins that can be rescued with L-Leu. Furthermore, the eIF2α phosphorylation suggests the possibility of an integrated stress response [26] that includes defective translation initiation that can be relieved with L-Leu.

### Low levels of translation initiation complexes in RBS cells are partially rescued by L-Leu

To further investigate translation initiation, we used pull downs to examine the formation of translation initiation complexes. 4EBP1 is a protein that prevents translation initiation when its unphosphorylated form interacts with eIF4E. Since L-Leu improved 4EBP1 phosphorylation in RBS cells (Fig. 1c, d), we further examined the 5’cap mRNA translation initiation complex using an m7GTP binding assay. m7GTP beads pull down more 4EBP1 protein in RBS cells compared to corrected cells (Fig. 1c). 4EBP1 binding to eIF4E inhibits the eIF4E-eIF4G interaction, blocking translation initiation. Consistently, eIF4G protein displayed less binding to m7GTP in RBS cells. The addition of L-Leu partially reduced 4EBP1 levels in m7GTP fraction, and restored eIF4G binding for RBS cells.

To further evaluate translation initiation complexes, we immunoprecipitated eIF4E and examined interacting proteins (Fig. 1d). We observed an enrichment of 4EBP1 in the eIF4E pull down in RBS cell lysates, and a marked reduction in eIF4G1. L-Leu treatment rescued eIF4G1- eIF4E association, and released the 4EBP1 inhibitory interaction. Finally, we pulled down eIF3B to assess eIF3B-eIF4E-eIF4G assembly of the 43S pre-initiation complex. The eIF4G1 and eIF4E proteins were present at lower levels in immunoprecipitations from RBS cells (Fig. 1e), but their levels were efficiently rescued by the addition of L-Leu. We also found that L-Leu partly rescued low levels of phospho-S6K1 in RBS fibroblasts (Fig. 1e). Collectively, the results clearly indicated that RBS fibroblasts have fewer translation initiation competent complexes. Their formation could be partially rescued by L-Leu supplement.

### L-Leu relieved translational efficiency for several gene classes in RBS

To systematically determine the translational efficiency of each mRNA in RBS fibroblasts, we used ribosome profiling and RNA deep sequencing. Wild-type, mutant, and corrected fibroblasts were treated with D-Leu (bioinactive form) or L-Leu. Ribosome profiling monitors translational efficiency by measuring ribosome-protected mRNA fragments (ribosome footprints) relative to the number of mRNAs [27]. We collected cells treated for 3 h to assess mRNAs with immediate translational changes in response to L-Leu. We also collected cells treated for 24 h to examine the long term effects of L-Leu on RBS cells. We detected exon-mapped ribosome footprints that corresponded to actively translated RefSeq mRNAs. Numbers of total reads per sample ranged from 5.0e + 06 to 3.0e + 07. The number of ribosome footprints that map to each mRNA divided by the number of total mRNA reads (gene-specific reads per million total exon-mapped reads, or RPKM) reflects the proportion of ribosomes engaged in the translation of that transcript. In our ribosome profiling and RNA seq analysis, the patterns between wild-type and *ESCO2*-corrected cells were similar, although not identical, at the transcriptional and translational levels (Additional file 1: Figure S2). The differences between the WT cells and the corrected cells could be due to the differences in genetic background as well as slight overexpression of ESCO2 in the corrected cells [12]. Given that the *ESCO2*-corrected cells mostly resembled WT cells, we decided to focus our analysis on the comparison of the mutant and corrected cells since they have the identical genetic backgrounds.

**Fig. 2:**
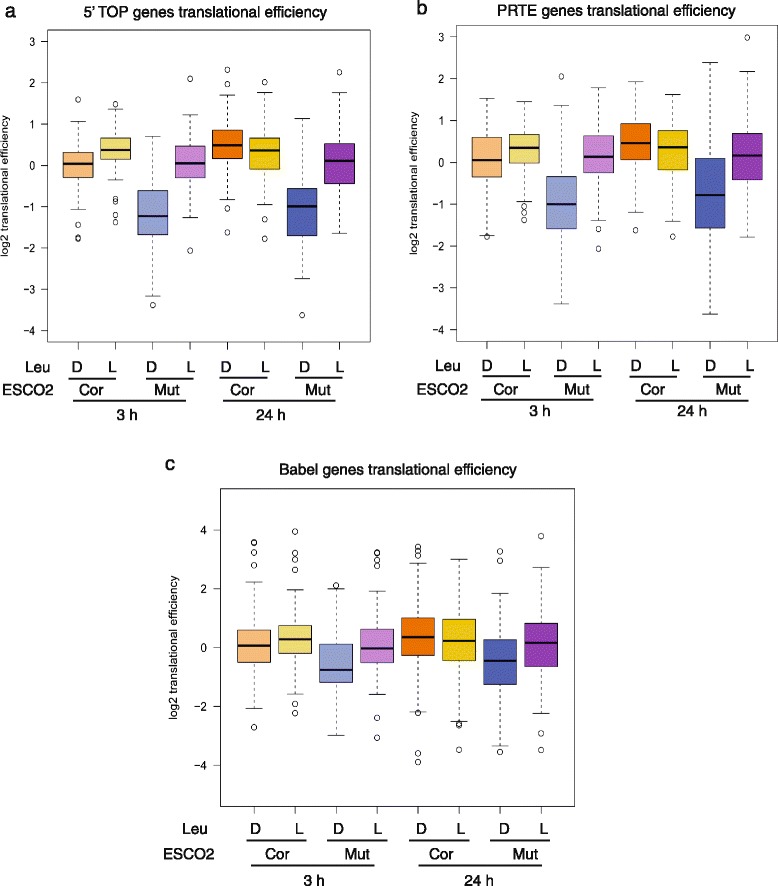
L-Leu increased translation of genes with poor translational efficiency in RBS cells. The corrected (cor) and RBS mutant (mut) cells were treated with either D-Leu or L-Leu for 3 h or 24 h. Cells were collected for ribosome profiling. **a** The boxplot shows the translational efficiency for genes with a 5’ TOP sequence. These mRNAs showed poor translational efficiency in RBS cells, which was partly rescued by L-Leu treatment. Corrected cells with D-Leu 24 h versus Mutant cells with D-Leu 24 h, *P* = 6.9e-22; Mutant cells with L-Leu 24 h versus Mutant cells with D-Leu 24 h, *P* = 4.4e-14; Corrected cells with D-Leu 3 h versus Mutant cells with D-Leu 3 h, *P* = 8.8e-16; Mutant cells with L-Leu 3 h versus Mutant cells with D-Leu 3 h, *P* = 1.9e-16. **b** The boxplot shows the translational efficiency for genes with a PRTE sequence. These mRNAs showed poor translational efficiency in RBS cells that was partially improved by L-Leu. Corrected cells with D-Leu 24 h versus Mutant cells with D-Leu 24 h, *P* = 7.1e-14; Mutant cells with L-Leu 24 h versus Mutant cells with D-Leu 24 h, *P* = 8.5e-9; Corrected cells with D-Leu 3 h versus Mutant cells with D-Leu 3 h, *P* = 2.8e-14; Mutant cells with L-Leu 3 h versus Mutant cells with D-Leu 3 h, *P* = 5.3e-15. **c** The boxplot shows the translational efficiency for genes previously defined to be hypersensitive to mTOR inhibition via Babel analysis [28]. These mRNAs showed poor translational efficiency in RBS cells that was partially improved by L-Leu. Corrected cells with D-Leu 24 h versus Mutant cells with D-Leu 24 h, *P* = 0.0002; Mutant cells with L-Leu 24 h versus Mutant cells with D-Leu 24 h, *P* = 0.14; Corrected cells with D-Leu 3 h versus Mutant cells with D-Leu 3 h, *P* = 3.5e-8; Mutant cells with L-Leu 3 h versus Mutant cells with D-Leu 3 h, *P* = 2.1e-6

Two recent studies have examined translational efficiency in the presence of mTOR chemical inhibitors [20, 21]. The translation of genes with 5' terminal oligopyrimidine (5′ TOP) motifs, which includes many ribosomal protein genes and a number of translation initiation and elongation factors, is particularly inefficient in the presence of these compounds [20, 21]. Strikingly, almost all 5’ TOP genes showed poor translational efficiency in RBS cells (Fig. 2a). This efficiency was partially rescued with L-Leu at both 3 and 24 h treatment, consistent with our Western blotting results for individual ribosomal protein subunits. Furthermore, the 5’ TOP genes were likely *de novo* translational targets of mTORC1 stimulation because they responded strongly at 3 h L-Leu treatment.

Hsieh et al. reported a second group of genes with a pyrimidine-rich translational element (PRTE) that were hyperdependent on mTOR-controlled translation [20]. Similar to the 5'terminal oligopyrimidine (5’TOP) genes, almost 90 % of these genes were poorly translated in RBS cells, but their translational efficiency improved dramatically with both 3 and 24 h of L-Leu supplementation (Fig. 2b). These genes function in various cellular processes such as glycogen storage (PGM1), cytokinesis (MYH14), mRNA metabolism (PABPC1), nuclear import (IPO7), protein transport (AP2A1), osteogenesis (CRTAP), nucleosome assembly (NAP1L1), and heat shock (HSPA8). Moreover, the PRTE genes, like the 5’TOP genes, were likely *de novo* translational targets of mTORC1 signaling because they responded strongly to 3 h treatment.

Previous analysis by another group using a bioinformatics approach known as Babel analysis identified genes with significantly reduced translation associated with impeded mTOR activity [28]. In addition to the PRTE and 5’TOP genes, this group of genes includes subunits of the eIF3 complex, and multiple Rab family Ras-related GTPases involved in endocytic trafficking. More than 60 % of these genes showed poor translational efficiency in RBS cells that was partially rescued with both 3 and 24 h L-Leu treatment (Fig. 2c; Additional file 2: Table S1). The mRNA levels of the 5’TOP, PRTE, and Babel gene groups were not significantly affected in mutant versus corrected cells, nor were they affected by L-leucine treatment (Additional file 1: Figure S3), strongly arguing for a translation-based rescue.

These results demonstrate that the gene groups that show exceptionally poor translation in response to pharmacological inhibition of mTORC1 also show poor translational activity in RBS cells. Furthermore, the translation of these same gene groups was partly improved by L-Leu treatment. For these gene groups, the effects of 3 and 24 h treatments were similar, suggesting that these gene groups contain many direct targets of the mTORC1 pathway. Taken together, these results are consistent with the conclusion that L-Leu activates mTORC1 function in RBS fibroblasts.

In addition to using predefined gene lists, we identified all genes whose translational efficiency was increased with L-Leu treatment in RBS cells. We used different methods to identify genes with improved translation efficiency: 1) Babel analysis, 2) fold change in translational efficiency greater than two and a minimum of 20 reads. For both methods, the gene ontology (GO) term analysis was very similar and included enrichment for ribosome components, translation initiation and elongation factors, protein targeting/sorting genes (co-translational process, and post-translational translocation), and RNA metabolism genes (Tables S2, S3, S4). We found that L-Leu improved the translational efficiency of more genes at the long timepoint (561 genes at 24 h *vs* 299 genes at 3 h). These results suggest that improved translational efficiency of direct mTORC1 targets at the short timepoint improved the translational efficiency of more genes following longer term treatment.

We used MEME to discover new motifs associated with genes with 2 fold or greater improved translational efficiency at 3 h [29]. Motif analysis identified 5’TOP and PRTE sequences, as expected, but also identified a new motif “CCAGGCTGGTCT” (Additional file 1: Figure S4). This motif does not correspond to any known transcription factor binding site. GO term analysis for genes with the new motif included translational elongation and translational termination, but also more surprisingly, protein localization to the endoplasmic reticulum, and mRNA catabolic processes (Additional file 1: Figure S4; Additional file 2: Table S5). mRNAs with the motif may represent newly discovered targets of mTORC1 dependent translation (Additional file 2: Table S6).

Loss of mTORC2 function has been shown to inhibit translation of many cell cycle proteins, including cohesin and condensin subunits such as SMC3, STAG1, NIPBL, SMC2 and SMC4 [30]. We wondered whether L-Leu stimulation would affect transcription or translation of these mTORC2 targets. While the transcription and translation of many of these genes is altered in the mutant as compared to corrected cells, they did not show a coherent response to L-Leu (Additional file 1: Figure S5; Additional file 2: Table S7), helping to confirm that L-Leu specifically boosted mTORC1-dependent translation.

### Poor mitochondrial function in RBS is partially improved by L-Leu

**Fig. 3:**
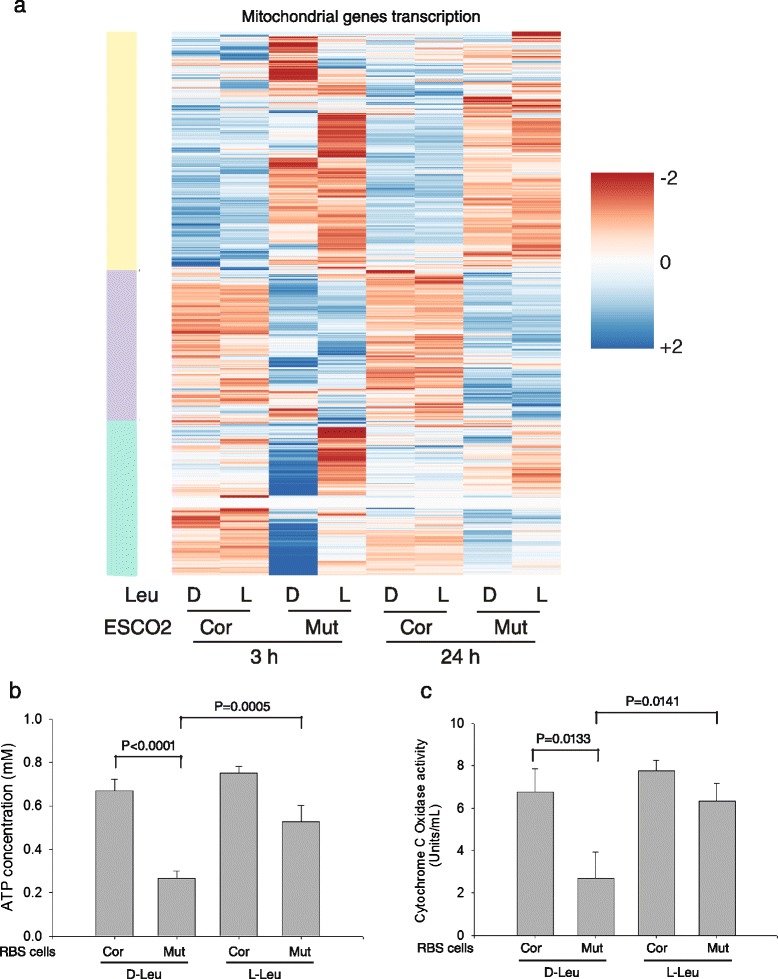
L-Leu treatment partially rescued mitochondrial function in RBS cells. **a** The heatmap shows that genes with mitochondrial function are differentially expressed in RBS cells. The yellow and purple bars indicate gene groups that are downregulated or upregulated, respectively, in the mutant cells, and are unresponsive to leucine. The subgroup that is affected by L-leucine treatment in the RBS cells is indicated by the green bar. See Additional file 2: Table S9 for GO terms for the leucine responsive cluster. **b** Intracellular ATP concentration was low in RBS cells but significantly improved by L-Leu treatment for 24 h. **c** Cytochrome c oxidase activity was impaired in RBS cells but significantly improved by L-Leu supplement for 24 h. For **b** and **c** error bars represent standard deviation of three biological replicates and the *p* value was calculated from a *t*-test

It has been reported that mTORC1 inhibition reduces mitochondrial biogenesis and activity via a 4EBP1 dependent mechanism [31]. We examined the behavior of 868 human genes annotated with cellular component “mitochondrial part.” We found that most of these genes are differentially expressed in the RBS cells compared to corrected cells (heatmap, Fig. 3a; Additional file 2: Table S8), and remain differentially expressed upon L-Leu treatment. However, about ~30 % of these genes are leucine responsive at both timepoints (green bar). The GO terms associated with the leucine responsive cluster are ATP synthesis (biological process), cytochrome C oxidase activity and NADH dehydrogenase activity (molecular function), and respiratory chain complex I (cellular compartment) (Additional file 2: Table S9).

We also examined the translational efficiency of the same 868 genes. We found that the translational efficiency of 32 genes showed improvement at 3 h and 96 genes at 24 h with L-Leu (Additional file 2: Table S8). GO terms associated with the genes that respond at 3 h were mitochondrial ribosome and respiratory chain, followed with mitochondrial membrane and metabolic processes related to the production of ATP, NADH, and cytochrome C at 24 h (Additional file 2: Table S10). Overall it appears that for a subset of genes with mitochondrial function, L-Leu treatment improved gene expression, and this occurred in conjunction with an effect on translational efficiency for a smaller group of genes in the RBS cells. The expression and translation of a subset of genes with mitochondrial function may be responsive to mTORC1 signaling. The genes with increased translational efficiency at 24 h in L-Leu are less likely to be direct targets of mTORC1 signaling, in contrast to the 5’TOP, PRTE, and Babel genes.

To further study whether the improved transcription and translation of mitochondrial genes with L-Leu manifested in improved mitochondrial function, we measured cellular ATP levels (Fig. 3b) and cytochrome c oxidase activity (Fig. 3c). These measures of mitochondrial activity in RBS cells were low compared to controls. Low ATP levels are associated with upregulation of the AMP-activated protein kinase (AMPK) signaling [32], and, consistently, we previously reported an increase in AMPK signaling in RBS [16]. Additionally, the dysfunction of cytochrome C oxidase activity elevates intracellular reactive oxygen species production [33, 34], a finding that correlates well with the previously reported increased ROS in RBS cells [16]. Both ATP levels and cytochrome C oxidase activity were partly rescued by stimulation of mTORC1 function with L-Leu. Collectively, our results indicate impaired mitochondrial gene expression, translation, and function are associated with RBS, and these defects can be partially restored by L-Leu supplementation, suggesting the defects may be due in part to low mTORC1 signaling.

### snoRNAs are differentially expressed in RBS cells

**Fig. 4:**
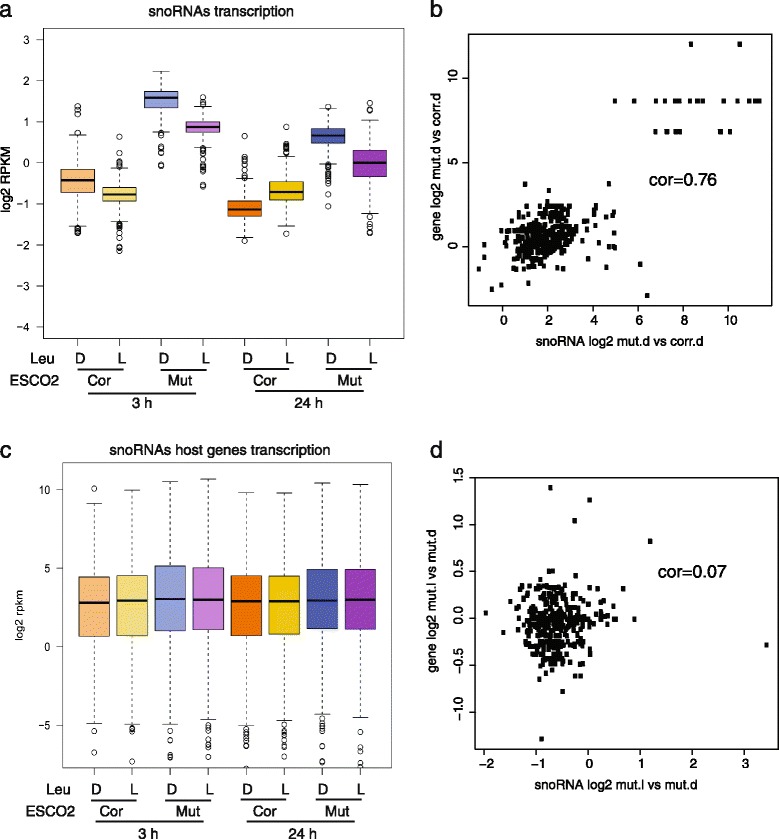
High levels of snoRNAs in RBS cells were partially reduced by L-Leu treatment. We selected a group of 379 snoRNA genes based on the biotype “snoRNA” and having the words “C/D box” or “H/ACA box” in the description field from ensembl. **a** The boxplot shows the expression of these genes is increased in RBS cells, but partly reduced with L-Leu treatment (gene data in Additional file 2: Table S11). Corrected cells with D-Leu 24 h versus Mutant cells with D-Leu 24 h, *P* = 1.4e-275; Mutant cells with L-Leu 24 h versus Mutant cells with D-Leu 24 h, *P* = 3e-53; Corrected cells with D-Leu 3 h versus Mutant cells with D-Leu 3 h, *P* = 7.6e-243; Mutant cells with L-Leu 3 h versus Mutant cells with D-Leu 3 h, *P* = 7.1e-98. *P* values in **a** and **c** were generated using a *t* test. **b** The scatter plot depicts the log2 fold change for snoRNAs in RBS mutant vs corrected at 3 h D-Leu (x axis) versus the same for host genes (y axis). The correlation is 0.76. **c** The boxplot shows the snoRNA host gene expression was not significantly different between corrected cells and mutant cells, and the host gene expression is not affected by L-Leu treatment. Corrected cells with D-Leu 24 h versus Mutant cells with D-Leu 24 h, *P* = 0.3; Mutant cells with L-Leu 24 h versus Mutant cells with D-Leu 24 h, *P* = 0.96; Corrected cells with D-Leu 3 h versus Mutant cells with D-Leu 3 h, *P* = 0.1; Mutant cells with L-Leu 3 h versus Mutant cells with D-Leu 3 h, *P* = 0.62. **d** The scatter plot depicts the log2 fold change for snoRNAs in RBS mutant L-Leu vs D-Leu at 3 h (x axis) versus the same for host genes (y axis). The correlation is 0.07

snoRNAs guide chemical modification of ribosomal RNAs, transfer RNAs, small nuclear RNAs, and mRNAs [35, 36]. C/D box snoRNAs guide methylation and H/ACA box snoRNAs guide pseudouridylation. These modifications can affect the stability of RNAs and their ability to interact with other RNAs and proteins. We examined the expression of snoRNAs in RBS cells. Many snoRNAs of both types were elevated in the RBS patient cells (Fig. 4a; Additional file 2: Table S11). L-Leu supplement significantly affected snoRNA levels in both the RBS and corrected cells, but had a bigger effect and partially reversed the increased levels of snoRNAs in RBS cells, suggesting that this gene group may be responsive to mTORC1 signaling.

Many snoRNA genes are hosted by ribosomal protein genes. We found a positive correlation (0.76) between snoRNA levels and the expression of their host gene for the *ESCO2*-mutant cells (Fig. 4b). However, host genes were relatively unaffected with L-Leu (Fig. 4c); the correlation between host genes and snoRNAs with L-Leu treatment was 0.07 (Fig. 4d). Taken together, these results suggest that the production of snoRNAs may be controlled by an unknown mTORC1-dependent mechanism that does not rely on the expression of the host genes. Nevertheless, the increase in snoRNAs in RBS cells has the potential to affect the modification and behavior of many RNAs.

### The imprinted *H19* and *GTL2* loci are differentially expressed in RBS cells

The cohesin complex has been shown to physically regulate expression via looping at the *IGF2-H19* imprinted region [22]. *IGF2-H19* shows a parent of origin specific monoallelic expression pattern that is important for embryogenesis and its disruption contributes to the etiology of several fetal disorders [23]. The long noncoding RNA H19 negatively regulates IGF2 (insulin growth factor 2), and *H19* deletion increases IGF2 signaling. In addition, H19 serves as a precursor of miRNA-675, which prevents *IGF-1R* (insulin-like growth factor 1 receptor) expression. *IGF-1R* activity promotes downstream PI3K/Akt/mTOR signaling [37, 38].

**Fig. 5:**
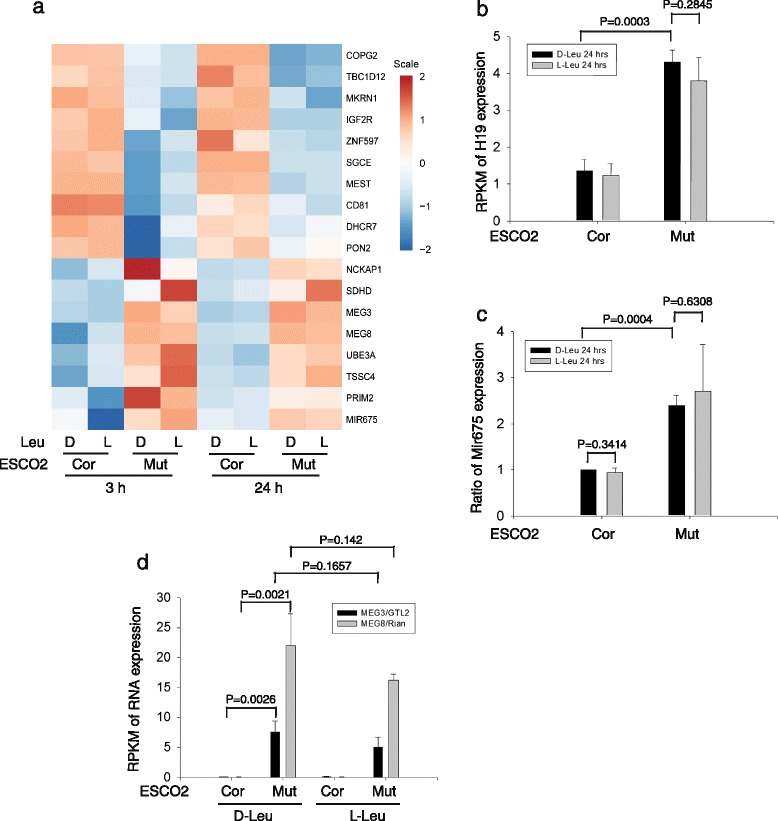
Imprinted genes are differentially expressed in RBS cells. **a** A heatmap displays the expression pattern of various imprinted genes in RBS and corrected cells with or without L-Leu. **b** The histograms show the average from three biological samples and the error bar indicates the standard deviation. The results are shown from 24 h treatment with L-Leu. The data from the 3 h treatment showed a similar pattern. H19 was upregulated ~ 4 fold in the mutant cells relative to the corrected cells. **c** The micro-RNA 675 was elevated ~ 3 fold in the mutant cells. **d** The imprinted *MEG3/GTL2* and *MEG8/Rian* genes were markedly increased in expression in RBS cells

We wondered whether the expression of imprinted loci was affected in RBS cells. In fact, several imprinted genes were differentially expressed in RBS cells (Fig. 5a). For example, RNAs involved in growth suppression and stem cell maintenance, including *MEG3/GTL2,* were present at higher levels. Another group of RNAs from imprinted genes were present at lower levels, including *MEST*, and the p53 repressor (*MKRN1*). *H19* was significantly elevated in RBS cells (Fig. 5b). Moreover, miRNA-675 was also elevated (Fig. 5c), as would be expected based on the elevation in *H19*. Since miRNA-675 may negatively regulate the PI3K-Akt- mTORC1 pathway, this H19/miRNA-675 elevation might contribute to mTORC1 depression in RBS. The differential expression of imprinted genes was not significantly affected by L-Leu, suggesting the differential expression of these genes is not due to low mTORC1 signaling, but may instead be related to defects in gene looping/chromosome architecture and/or altered DNA methylation patterns [39].

*MEG3/GTL2-DLK1* is another imprinted locus regulated by differentially methylated regions (DMRs). Cohesin has been reported to colocalize with CTCF at this locus where it binds to the *GTL2* DMR on the unmethylated maternal allele [40, 41], potentially playing a repressive function for *MEG3/GTL2* expression [40]. Cohesin binds to the *GTL2* DMR on the unmethylated maternal allele. *GTL2* is a growth suppressor that strongly activates p53 expression. *GTL2* regulates maternal expression of an miRNA cluster [42–47], where it promotes the expression of miRNAs that could reduce mTORC1 signaling by inhibiting translation of their target mRNAs [48, 49]. Both *GTL2* mRNA and *GTL2*-regulated miRNAs showed an elevation in RBS cells by RNA seq analysis which was verified by qPCR (Fig. 5d; Additional file 1: Figure S6). We also found a significant increase in *MEG8/Rian* in RBS cells. *MEG8* is a maternally expressed, imprinted long non-coding RNA transcribed from the same DNA as *GTL2*. Our results suggest that loss of cohesin acetyltransferase function alters expression from the *IGF2-H19* and *MEG3/GTL2-DLK1* imprinted loci, providing one possible speculative mechanism by which cohesin could influence mTORC1 signaling and translation.

### L-Leu independent differential gene expression in RBS cells

Homeobox (*HOX*) genes are a group of transcription factors that determine the anterior-posterior axis of an embryo. Recent work indicates that cohesin influences *HOX* gene expression through chromatin architecture organization [3, 50–52]. We examined *HOX* gene expression in RBS cells and found that the expression of many *HOX* A, B, C, and D subunits is reduced [3], independent of L-Leu treatment, while translation is unaffected (Additional file 1: Figure S7). Although taken all together the differential expression for this gene group is not statistically significant between mutant and corrected cells, the reduced expression of many individual genes is significant, and this reduced expression is apparent in the stretch of the boxes into the negative log2 values. The data suggest that *ESCO2* dependent *HOX* gene expression and architecture is independent of *ESCO2*-induced mTORC1 defects.

There are over 6000 differentially expressed genes in RBS cells, but ~1000 fewer with L-Leu treatment, suggesting a significant fraction of the differential expression could be translation-driven. The most notable and top GO term for the upregulated genes in the RBS mutant cells treated with L-Leu vs D-Leu at 3 h is “respiratory chain complex I” (Additional file 2: Table S12). However, the differential expression of particular gene groups in RBS cells, such as *HOX* genes and imprinted genes, was independent of L-Leu. We previously reported that the nucleolar architecture in RBS cells is only rescued by *ESCO2* replacement, not by L-Leu addition [16]. Similar to the rDNA, these loci may be examples where basic chromatin architecture is dependent on cohesin acetylation and cannot be rescued with leucine.

**Fig. 6:**
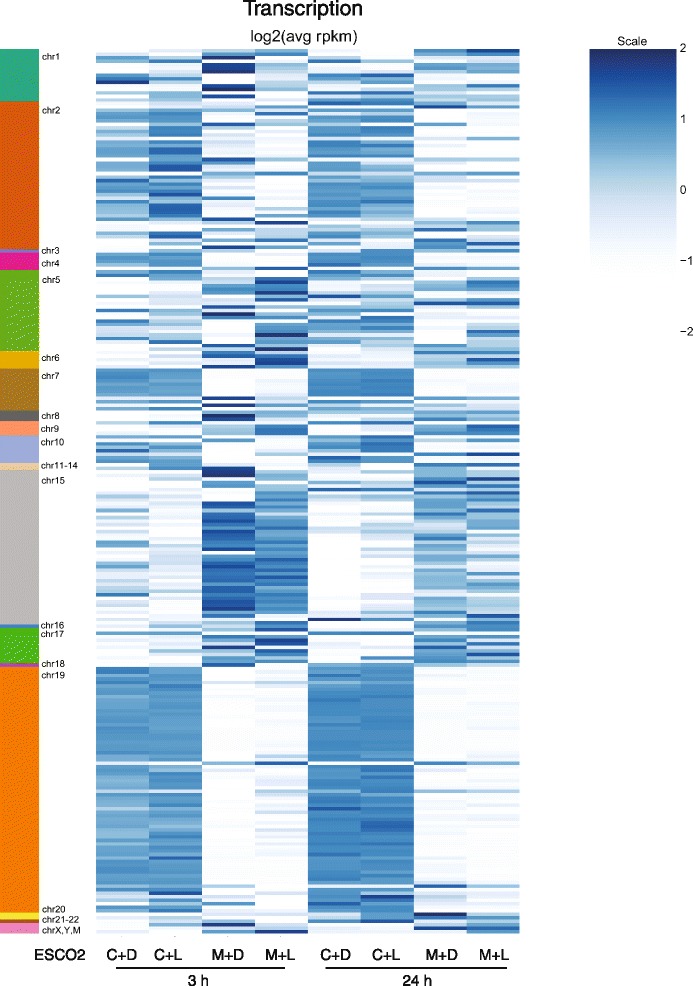
Nucleolar associated genes are differentially expressed in RBS cells. Expression levels of genes located in nucleolar associated domains (NADs, indicated on the left) is shown in a heatmap for corrected cells (C) and RBS cells (M) treated with either D-Leu (D) or L-Leu (L) for 3 or 24 h. Within each domain, genes tend to show a similar pattern, although some domains show increased expression in the corrected cells while others show reduced expression. In general these patterns are unaffected by the addition of L-leucine. Data can be found in Additional file 2: Table S13

Previous work has shown that regions from almost all human chromosomes associate with nucleoli [53, 54]. These regions have been termed NADs for nucleolar associated domains. Given the disruption of nucleolar morphology in the RBS cells, we wondered whether the expression of genes within NADs was affected. We found that genes in many of the domains were differentially expressed in RBS cells (Fig. 6, Additional file 2: Table S13). In some cases genes that were not expressed in RBS cells became expressed in the corrected cells, and in other cases the reverse occurred. Whichever trend was observed was true for most of the genes within that NAD, arguing that the domains are behaving as a unit. The differential expression was mostly independent of L-Leu, consistent with the lack of rescue of the nucleolar morphology with L-Leu. We conclude that disruption of nucleolar morphology has the potential to affect the expression of genes normally associated with nucleoli.

## Discussion

We provide compelling molecular evidence that L-Leu can partially rescue translation initiation and mitochondrial function via its stimulation of mTORC1 in RBS cells. Ribosome profiling combined with RNA seq allowed us to evaluate the contribution of differential expression and translation in RBS. We speculate that L-Leu provides partial rescue of translation and translation-dependent gene expression without rescuing cohesin dependent chromatin organization. Our data, combined with the existing literature, argues that at least two different mechanisms generate differential gene expression in RBS cells: 1) defects in looping or other aspects of chromosome architecture and 2) loss of translation function. This second mechanism can be targeted by L-Leu stimulation of mTORC1 signaling. Our work suggests the possibility that a few critical loci that contribute to translation, such as imprinted genes and rDNA, play a key role in the RBS disorder. L-Leu treatment allows us to begin to distinguish between differential gene expression that is dependent on mTORC1 signaling versus independent in the *ESCO2* mutant. Our studies suggest targeting translation with the non-toxic amino acid L-Leu may be a productive strategy in human diseases with poor translation, such as the cohesinopathies.

Additional human diseases with defects in translation include the ribosomopathies, diseases caused by defects in ribosome biogenesis [55]. Stimulation of the TOR pathway with L-Leu in the ribosomopathy Diamond Blackfan anemia has shown promise as a therapeutic [56, 57] and is currently being tested in a Phase I clinical trial. Translational control is emerging as an important regulatory mechanism for many different cell types, such as germline stem cells, hematopoietic stem cells, and neurons [58]. Another process affected in over 40 different human diseases is mitochondrial function. We provide the first evidence that mitochondrial function is affected in RBS fibroblasts, and we further report that L-Leu stimulation of mTORC1 could promote both translation initiation and mitochondrial function. Together these findings suggest that it will be important to study the role of mTORC1 in embryo development, cell identity, and mitochondrial function in order to discover strategies to target this pathway to treat human disease associated with translation impairment.

How L-Leu stimulates mTORC1 has been the topic of debate. Two groups have argued that the charged form of the leucine tRNA synthetase is a key factor in the activation of the Ragulator complex that activates TOR [59, 60]. Others have argued that the mechanism may involve direct sensing of amino acids in the lysosome, where mTORC1 is located during activation [61, 62]. While the exact molecular mechanism is still unclear, our work provides a detailed molecular picture of how transcription and translation respond to L-Leu activation of mTORC1. We found evidence that the 5’TOP and PRTE-containing mRNAs respond more quickly to L-Leu than other targets, such as mRNAs with mitochondrial function, suggesting that mTORC1 stimulation affects immediate targets followed by secondary targets. Our study also suggests that pharmacological inhibition of mTORC1 with small molecules has similar effects on translational efficiency as genetically-induced depression by *ESCO2* mutation.

Cohesin and CTCF (CCCTC-binding factor) co-localize at a significant fraction of cohesin binding sites where these proteins likely directly regulate genome architecture and gene expression. Some of these sites include the imprinted genes and the *HOX* genes. Recently, the Hi-C method has identified that loss of cohesin or CTCF function not only causes the loss of some chromatin interactions, but also leads to the gain of other interactions [63]. Cohesin appears to positively regulate some loci and negatively regulate others, making the effects of loss of function difficult to predict. However, we have previously provided evidence that acetylated cohesin promotes expression of the ribosomal DNA repeats and the formation of nucleoli. CTCF is also important for the formation of nucleoli [64]. Nucleoli likely provide a keystone for genome architecture [64–67], suggesting that disruption in the organization of rDNA could have extensive effects on the organization and expression of most chromosomes such as that observed at NADs. In the future it will be interesting to analyze chromosome organization in the absence of *ESCO2* function and how this impacts gene expression.

Partial loss of function in many different cohesin related genes (*ESCO2, SMC1, SMC3, NIPBL, RAD21*) causes translation defects in yeast, zebrafish, and human cells [1, 4, 14, 16, 18, 19]. Collectively, these observations suggest that one evolutionarily conserved function of cohesin is to couple chromosome structure with the translational output of the cell. The mechanisms that link these processes may include the role of cohesin in bringing two DNA sequences together such as (1) looping of promoter and terminator within the rDNA for effective polymerase recycling and rRNA production, (2) gathering of the rDNA repeats into a functional nucleolus which may provide an anchor point for genome architecture, and (3) regulation of looping of promoter and regulatory sequences and therefore expression at imprinted loci and *HOX* genes. Additional mechanisms for coupling chromosome structure to translation may exist. This study provides a gene by gene understanding of how L-Leu stimulation of mTORC1 affects gene expression and translation.

## Conclusions

*ESCO2*, a gene encoding an acetyltransferase for cohesin, is required for normal gene expression and translation. We characterized the effect of L-Leu on translation initiation complexes, mitochondrial function, gene expression and translational efficiency in *ESCO2* mutant RBS cells in which mTORC1 signaling is depressed. We find L-Leu provides a significant rescue effect for all of these measures. L-Leu activation of mTORC1 function may be a useful approach for human diseases with disruption of these functions.

## Methods

### Reagents

Reagents were obtained from the following sources: antibodies to S6K1, 4EBP1, eIF4E, phospho-S51 eIF2α, eIF2α, eIF4G1 from Cell Signaling; antibodies to eIF3b (N20), α-tubulin, S6K, RPS7, RPS19, RPL5 and horseradish-peroxidase-labelled anti-mouse, anti-goat and anti-rabbit secondary antibodies from Santa Cruz Biotechnology; Anti-p70S6K1 (phospho T389) antibody from Abcam company; antibodies to RPL23 from Sigma company; antibodies to RPL24 from Genetex company. Complete Protease Mixture from Roche Applied Science; cycloheximide from Sigma; immobilized 2’/3’-EDA-7-methyl-GTP- agarose beads from Jena Bioscience GmbH, Germany; DMEM from Life Technologies Inc.; inactivated fetal calf serum from Invitrogen; Dynabeads® magnetic separation system from Life Technologies Inc.

### Preparation of cell lysates and affinity purifications

Cells were rinsed once with ice-cold PBS and lysed in ice-cold lysis buffer (buffer A: 50 mM HEPES-KOH (pH 7.4), 2 mM EDTA, 10 mM pyrophosphate, 10 mM β-glycerophosphate, 40 mM NaCl, 1 % Trition X-100 and one tablet of EDTA-free protease inhibitors (Roche) per 25 mL. The soluble fraction of the cell lysate was isolated by centrifugation at 12,000 g for 10 min. For immunoprecipitations, primary antibodies were added to lysates and incubated with rotation for overnight at 4 °C. 20 μl of a 50 % slurry of protein G-dynabeads were then added and the incubation continued for an additional 3 h. Immunoprecipitates were washed three times with lysis buffer. Immunoprecipitated proteins were denatured by the addition of 20 μl of sample buffer and boiled for 5 min, resolved by 8–16 % SDS–PAGE, and analyzed by Western blot. For m7GTP affinity purifications, 2’/3’-EDA-7-methyl-GTP- agarose beads were washed with lysis buffer. 20 μl of beads from a 50 % slurry was added to cell lysates and incubated with rotation overnight at 4 °C. Beads were washed three times with lysis buffer, denatured by the addition of 50 μl sample buffer, and analyzed by Western blot.

### Preparation of ribosome profiling samples

Ribosome profiling samples were essentially collected and processed as described in the Mammalian ARTseq™ Ribosome Profiling Kit (Epicentre, cat. no. RPHMR12126, protocol version 2012). In brief, human wild-type fibroblasts, *ESCO2*-mutant RBS fibroblasts, and *ESCO2*-corrected RBS fibroblasts were grown on 15-cm plates in DMEM medium supplemented with 10 % Fetal Bovine Serum (FBS). At 80 % confluence, the cells were washed two times with PBS, and grown for another 24 h in DMEM/10 % FBS. Subsequently, the cells were supplemented with 10 mM D-leucine (D-Leu) or L-Leu and incubated for either 3 or 24 h. Prior to lysis, the cells were incubated for 1 min in DMEM containing 0.1 mg/ml cycloheximide (US Biological, cat. no. C8500-10). After a rinse with ice cold PBS containing 0.1 mg/ml cycloheximide, the cells from up to three plates (~20–60, 000,000 cells) were collected by scraping into 800 μl chilled Mammalian Lysis Buffer (ART-Seq 1X Mammalian Polysome Buffer, 1 % Triton X-100, 0.1 % NP-40, 1 mM DTT, 10 U/ml DNase I, 0.1 mg/ml cycloheximide). The cell lysate was triturated 10 times through a 26G needle to insure complete lysis, incubated for 10 min on ice with periodic agitation, and clarified by a 10 min centrifugation at 20,000 x g at 4 °C. The RNA concentration of the supernatant was determined using the Quant-iT RiboGreen RNA Assay Kit (Invitrogen) and a SpectraMax M2 reader (Molecular Devices) according to manufacturer’s instructions. Aliquots were flash-frozen in liquid nitrogen and stored at −80 °C. Ribosome-protected mRNA fragments (RPFs) and fragmented total RNA depleted for ribosomal RNA were sequenced for each sample. Footprinting was performed at room temperature for 45 min using ART-Seq Nuclease (Epicentre), then stopped with SUPERase•In™ RNase Inhibitor (Life Technologies). For the 24 h timepoint samples, 400 μl of clarified lysate was used and 2.8 units of nuclease was added per μg of RNA. For the 3 h timepoint samples, 300 μl of lysate was used and the amount of nuclease was reduced to 0.5 units/μg of RNA. RPFs were isolated from 200 to 400 μl of the nuclease/SUPERase•In™ treated lysate using 2–4 Sephacryl S400 columns (GE Healthcare: MicroSpin S-400 HR, cat. no. 27-5140-01), followed by acid phenol:chloroform extraction and isopropanol precipitation. Total RNA was isolated from untreated lysate by acid phenol:chloroform extraction and isopropanol precipitation. Ribosomal RNA was removed from 1–5 μg of RPFs and total RNA using either the Ribo-Zero™ Magnetic or the Ribo-Zero™ Magnetic Gold Kit (Human/Mouse/Rat) (Epicentre). To purify the Ribo-Zero treated samples, the RNA Clean & Concentrator™-5 Kit (Zymo Research) was used as described in the ART-Seq protocol. RPFs in the range of 26–34 nt were size selected by PAGE purification and the total RNA was heat fragmented. Libraries were constructed as described in the ART-Seq protocol. The desired 140–160 bp PCR-amplified libraries were purified from excess adapter-only product (~113 bp) by PAGE purification. Library pools were sequenced on a HiSeq 2500 System (Illumina) with the 50 bp Single-End read protocol.

### RNA sequencing analyses

Before alignment, ribosome footprint and total mRNA libraries were processed to remove cloning artifacts. Processed reads were then aligned to a database of human rRNA sequences using the bowtie2 short-read alignment program (version 2.1.0 with parameters -k 1 -N 1 --local) to remove reads from ribosomal RNA. A very small percentage of reads aligned to rRNA sequences in most cases, ranging from 0.2 to 12 %. The remaining reads were then aligned to the hg19 human genome using tophat (version 2.0.8 with parameters --segment-mismatches 1 -x1 -g1 --no-coverage-search). Translational efficiency was calculated as footprint RPKM/mRNA RPKM. Values from biological replicates were averaged together.

### Gene ontology analysis

To determine enrichment for gene ontology categories, differentially expressed or translated genes were analyzed using the GeneAnswers package in R (version 3.0.2). Representative gene ontology categories from each contrast with a *P* value < 0.05 were selected.

### Cellular ATP assay in RBS cells

Cells were cultured as for ribosome profiling. Samples were prepared according to the instructions provided by the ATP Colorimetric/Fluorometric Assay Kit of the BioVision company. Cells (1 × 10^6^) were lysed in 100 μl of ATP Assay Buffer and centrifuged at 4 °C at 15,000 g for 2 min to pellet insoluble material. 2–50 μl supernatant was added to a well in a 96-well plate, and the final volume was adjusted to 50 μl/well with ATP Assay Buffer. Samples were tested at several doses to make sure the readings were within the standard curve range.

### Mitochondria isolation for cultured cells and cytochrome c oxidase assay

Cells were cultured as for ribosome profiling. Mitochondria were isolated according to the instructions provided by the Mitochondria isolation kit from the Thermo Scientific company. For the cytochrome c oxidase assay, we used the Cytochrome c Oxidase Assay Kit from Sigma-Aldrich. The reaction was started by the addition of 50 ml of Ferrocytochrome c Substrate Solution and mixed by inversion. Absorbance was read at A_550_/min immediately due to the rapid reaction rate of this enzyme. Background values were between 0.001 and 0.005 A_550_/min.

### Real-Time quantitative PCR of microRNAs in RBS cells

Total RNA (50–200 ng/μl) was extracted from *ESCO2*-mutant RBS cells or corrected RBS cells with L-Leu or D-Leu treatment. The RNA concentration was measured by Bioanalyzer RNA Nano chip, and normalized for reverse transcription (RT). For each 15 μl RT reaction, total RNA (10 ng of total RNA per 15 μl reaction) was combined with the RT master mix (TaqMan® MicroRNA Reverse Transcription Kit, PN4366596, Applied Biosystems). 3 μl of the RT primers was transferred to the appropriate tubes, and reactions were subjected to thermal cycling. TaqMan gene expression assays (Applied Biosystems) were performed on triplicate samples with a 7500 Real-Time cycler (Applied Biosystems). U6 snRNA serves as a reference control. The TaqMan gene expression assays were performed according to the manufacturer’s instructions. All qRT-PCR was performed using TaqMan probes.

### Statistical analysis

The results are reported as mean values ± standard error (mean ± s.e.). Statistical analysis was performed by Student's *t*-test with SigmaPlot-Systat Software (Sigmaplot Software Inc). An ANOVA two-way model was used to compare continuous variables. A *P* value <0.05 was considered statistically significant.

### Description of additional data

Additional data include seven figures and thirteen tables.

### Accession numbers

The data set supporting the results of this article is available in the Gene Expression Omnibus repository, [#GSE64962].

### Data availability

Original data underlying this manuscript can be accessed from the Stowers Original Data Repository at http://www.stowers.org/research/publications/libpb-1023.

## Additional files

Additional file 1: Figure S1. Quantitation of Western blotting in Fig. 1a and b. **Figure S2.** Gene transcription and translation pattern of WT cells and ESCO2-Corrected cells. **Figure S3.** The boxplots display the show expression of mRNAs with 5’TOP sequences (a), PRTE sequences (b), and Babel genes (c). **Figure S4.** Motif and GO terms associated with genes with increased translational efficiency upon L-leucine treatment in RBS cells. **Figure S5.** mTORC2 show regulated genes do not show a coherent response to L-leucine. **Figure S6.** GTL2-regulated miRNAs are increased in RBS cells independent of L-Leu. **Figure S7.** Homeobox (HOX) gene expression is reduced in RBS cells. (ZIP 4.64 MB) Additional file 2: Table S1. Summary table of Babel analysis. **Table S2.** GO term analysis of genes that show improved translational efficiency in RBS cells with L-leucine. **Table S3.** List for genes with fold change in translational efficiency greater than two and a total number of reads over 20 counts at 3 h. **Table S4.** List for genes with fold change in translational efficiency greater than two and a total number of reads over 20 counts at 24 h. **Table S5.** GO term analysis for genes with the new motif. **Table S6.** List of genes that have the new motif. **Table S7.** Summary table of data in boxplot of mTORC2-regulated gene show transcription and translation in Figure S5. **Table S8.** Summary table of data in heatmap for 868 mitochondrial genes—expression (Fig. 3) and translation. **Table S9.** GO term analysis for mitochondrial genes that show improve transcription with L-leucine. **Table S10.** GO term analysis for mitochondrial genes that improve translation with L-leucine. **Table S11.** Summary table of data in boxplot of snoRNAs expression in Fig. 4. **Table S12.** GO term analysis of differentially expressed genes in RBS cells. **Table S13.** List of genes in NADs and their expression, from Fig. 6. (ZIP 1.78 MB)

## Abbreviations

4EBP1: Eukaryotic translation initiation factor 4E-binding protein 1
5’TOP: 5'terminal oligopyrimidine
AMPK: AMP-activated protein kinase
CdLS: Cornelia de Lange syndrome
D-Leu: D-leucine
DMR: Differentially methylated regions
eIF4E: eukaryotic translation initiation factor 4E
GO: Gene ontology
*HOX*: Homeobox
IGF-1R: Insulin-like growth factor 1 receptor
IGF2: Insulin growth factor 2
L-Leu: L-leucine
NAD: Nucleolar associated domains
PRTE: Pyrimidine-rich translational element
RBS: Roberts syndrome
rDNA: ribosomal DNA
rRNA: ribosomal RNA
ribosome footprint: ribosome-protected mRNA fragments
RPKM: Gene-specific reads per million total exon-mapped reads.
