# Supplementary material for: Improved transcription and translation with L-leucine stimulation of mTORC1 in Roberts syndrome
Source: BMC Genomics. 2016 Jan 5;17:25. doi: 10.1186/s12864-015-2354-y (PMC4700579; doi:10.1186/s12864-015-2354-y)
Supplement: Additional file 1: Figure S1. — Quantitation of Western blotting in Fig. 1a and b. Figure S2. Gene transcription and translation pattern of WT cells and ESCO2-Corrected cells. Figure S3. The boxplots display the show expression of mRNAs with 5’TOP sequences (a), PRTE sequences (b), and Babel genes (c). Figure S4. Motif and GO terms associated with genes with increased translational efficiency upon L-leucine treatment in RBS cells. Figure S5. mTORC2 show regulated genes do not show a coherent response to L-leucine. Figure S6. GTL2-regulated miRNAs are increased in RBS cells independent of L-Leu. Figure S7. Homeobox (HOX) gene expression is reduced in RBS cells. (ZIP 4.64 MB) [file 12864_2015_2354_MOESM1_ESM.zip › Figure S1leg.pdf]

Figure S1

a

Relative percent of protein expression (%)

ESCO2	WT	Mut	Cor	
	78.4	44.9	81.4	RPL5
	61.7	19.1	35.6	RPL23
	117.4	42.5	90.1	RPL24
	15.3	9.4	17.5	RPS7
	65.4	33.7	49.7	RPS19
	100	100	100	α-Tubulin

b

Relative percent of protein expression (%)

	D-Leu		L-Leu		
ESCO2	Cor	Mut	Cor	Mut	
	1.3	9.5	0.9	2.8	p-eIF2α
	98.5	92.1	110.7	157.5	eIF2α
	137.2	68.9	188.1	171.4	RPS7
	106.1	54.5	135.3	127.6	RPSL24
	100	100	100	100	α-Tubulin

ImageQuant TL quantitation of Western blotting in Figure 1a and b. (a) Relative percentages of protein expression in Fig. 1a, (b) Relative percentages of protein expression in Fig. 1b. The α-tubulin level was used as the reference.
