# Supplementary material for: Improved transcription and translation with L-leucine stimulation of mTORC1 in Roberts syndrome
Source: BMC Genomics. 2016 Jan 5;17:25. doi: 10.1186/s12864-015-2354-y (PMC4700579; doi:10.1186/s12864-015-2354-y)
Supplement: Additional file 1: Figure S1. — Quantitation of Western blotting in Fig. 1a and b. Figure S2. Gene transcription and translation pattern of WT cells and ESCO2-Corrected cells. Figure S3. The boxplots display the show expression of mRNAs with 5’TOP sequences (a), PRTE sequences (b), and Babel genes (c). Figure S4. Motif and GO terms associated with genes with increased translational efficiency upon L-leucine treatment in RBS cells. Figure S5. mTORC2 show regulated genes do not show a coherent response to L-leucine. Figure S6. GTL2-regulated miRNAs are increased in RBS cells independent of L-Leu. Figure S7. Homeobox (HOX) gene expression is reduced in RBS cells. (ZIP 4.64 MB) [file 12864_2015_2354_MOESM1_ESM.zip › Figure S6leg.pdf]

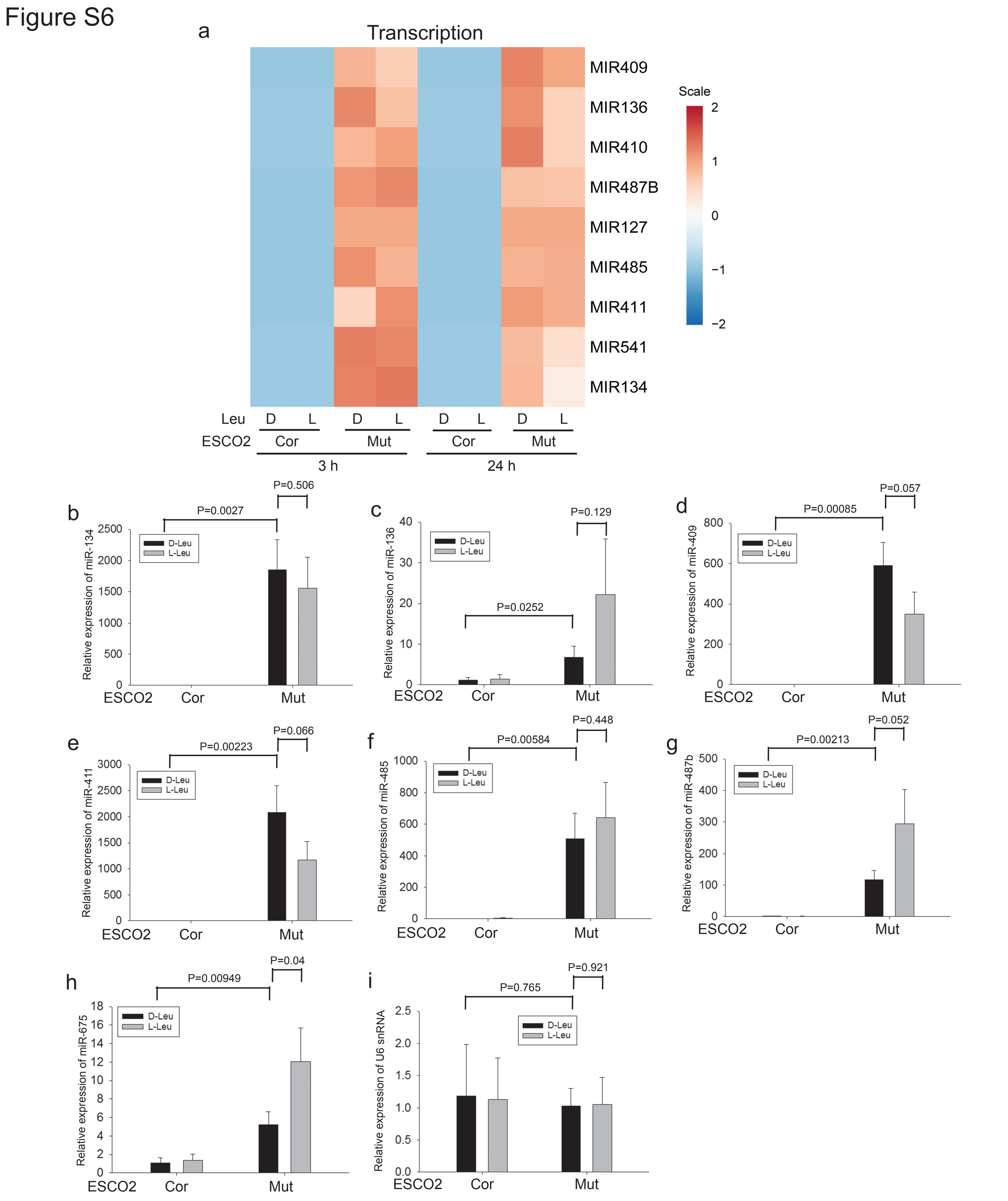

GTL2-regulated miRNAs are increased in RBS cells independent of L-Leu treatment (a) Heatmap of GTL2-regulated miRNA expression by RNA seq analysis. In Corrected cells, no reads were detected. (b-i) GTL2-regulated miRNAs are increased in RBS cells independent of L-Leu treatment as shown by quantitative real-time PCR. The miR-134 and miR-411 levels were elevated several thousand-fold in the RBS cells with or without L-Leu treatment (b, e) The miR-409, miR-485, and miR-487b levels were upregulated several hundred-fold with the ESCO2 mutation (d, f, g) The miR-136 and miR-675 expression increased 5–20 fold in the mutant cells (c, h) L-leucine supplement does not seem to reverse the miRNAs elevation in the mutant cells. The internal control U6 snRNA expression was nearly identical in all samples (i) (b) miR-134, P = 0.0027: Mutant with D-Leu versus Corrected with D-Leu, P = 0.506: Mutant with L-Leu versus Mutant with D-Leu. (c) miR-136, P = 0.0252: Mutant with D-Leu versus Corrected with D-Leu, P = 0.129: Mutant with L-Leu versus Mutant with D-Leu. (d) miR-409, P = 0.00085: Mutant with D-Leu versus Corrected with D-Leu, P = 0.057: Mutant with L-Leu versus Mutant with D-Leu. (e) miR-411, P = 0.00223: Mutant with D-Leu versus Corrected with D-Leu, P = 0.066: Mutant with L-Leu versus Mutant with D-Leu. (f) miR-485, P = 0.00584: Mutant with D-Leu versus Corrected with D-Leu, P = 0.448: Mutant with L-Leu versus Mutant with D-Leu. (g) miR-487b, P = 0.00213: Mutant with D-Leu versus Corrected with D-Leu, P = 0.052: Mutant with L-Leu versus Mutant with D-Leu. (h) miR-675, P = 0.00949: Mutant with D-Leu versus Corrected with D-Leu, P = 0.04: Mutant with L-Leu versus Mutant with D-Leu. (I) U6 snRNA, P = 0.765: Mutant with D-Leu versus Corrected with D-Leu, P = 0.921: Mutant with L-Leu versus Mutant with D-Leu.
