# Supplementary material for: Improved transcription and translation with L-leucine stimulation of mTORC1 in Roberts syndrome
Source: BMC Genomics. 2016 Jan 5;17:25. doi: 10.1186/s12864-015-2354-y (PMC4700579; doi:10.1186/s12864-015-2354-y)
Supplement: Additional file 1: Figure S1. — Quantitation of Western blotting in Fig. 1a and b. Figure S2. Gene transcription and translation pattern of WT cells and ESCO2-Corrected cells. Figure S3. The boxplots display the show expression of mRNAs with 5’TOP sequences (a), PRTE sequences (b), and Babel genes (c). Figure S4. Motif and GO terms associated with genes with increased translational efficiency upon L-leucine treatment in RBS cells. Figure S5. mTORC2 show regulated genes do not show a coherent response to L-leucine. Figure S6. GTL2-regulated miRNAs are increased in RBS cells independent of L-Leu. Figure S7. Homeobox (HOX) gene expression is reduced in RBS cells. (ZIP 4.64 MB) [file 12864_2015_2354_MOESM1_ESM.zip › Figure S7leg.pdf]

Figure S7

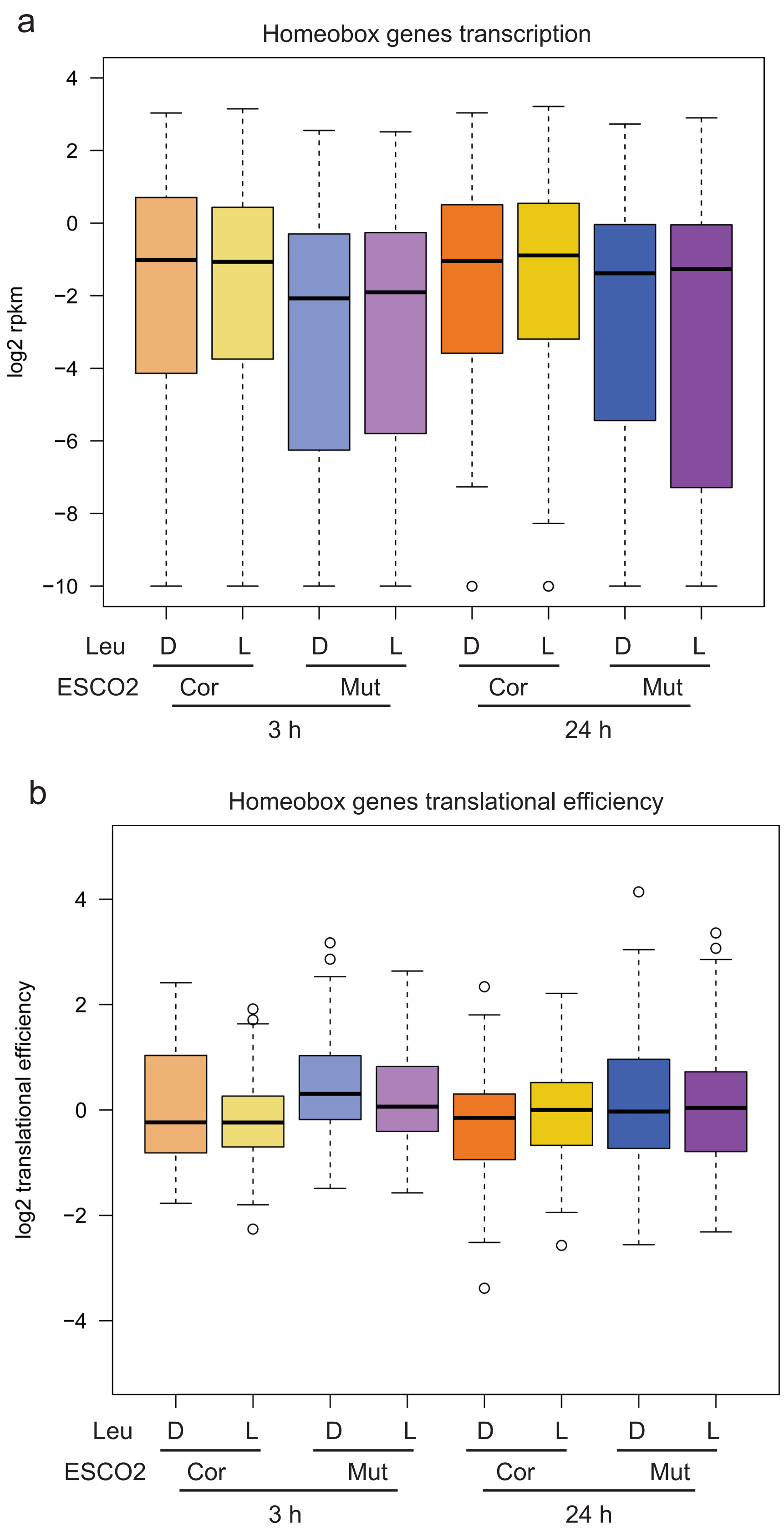

Homeobox (HOX) gene expression is reduced in RBS cells. The boxplots show HOX gene expression (a) and translational efficiency (b) in RBS cells. We performed statistical analysis for these boxplots. (a) Corrected cells with D-Leu 24 h versus Mutant cells with D-Leu 24 h, $P = 0.33$; Mutant cells with L-Leu 24 h versus Mutant cells with D-Leu 24 h, $P = 0.54$; Corrected cells with D-Leu 3 h versus Mutant cells with D-Leu 3 h, $P = 0.16$; Mutant cells with L-Leu 3 h versus Mutant cells with D-Leu 3 h, $P = 0.83$. (b) Corrected cells with D-Leu 24 h versus Mutant cells with D-Leu 24 h, $P = 0.51$; Mutant cells with L-Leu 24 h versus Mutant cells with D-Leu 24 h, $P = 0.69$; Corrected cells with D-Leu 3 h versus Mutant cells with D-Leu 3 h, $P = 0.07$; Mutant cells with L-Leu 3 h versus Mutant cells with D-Leu 3 h, $P = 0.93$.
