# Supplementary material for: Improved transcription and translation with L-leucine stimulation of mTORC1 in Roberts syndrome
Source: BMC Genomics. 2016 Jan 5;17:25. doi: 10.1186/s12864-015-2354-y (PMC4700579; doi:10.1186/s12864-015-2354-y)
Supplement: Additional file 2: Table S1. — Summary table of Babel analysis. Table S2. GO term analysis of genes that show improved translational efficiency in RBS cells with L-leucine. Table S3. List for genes with fold change in translational efficiency greater than two and a total number of reads over 20 counts at 3 h. Table S4. List for genes with fold change in translational efficiency greater than two and a total number of reads over 20 counts at 24 h. Table S5. GO term analysis for genes with the new motif. Table S6. List of genes that have the new motif. Table S7. Summary table of data in boxplot of mTORC2-regulated gene show transcription and translation in Figure S5. Table S8. Summary table of data in heatmap for 868 mitochondrial genes—expression (Fig. 3) and translation. Table S9. GO term analysis for mitochondrial genes that show improve transcription with L-leucine. Table S10. GO term analysis for mitochondrial genes that improve translation with L-leucine. Table S11. Summary table of data in boxplot of snoRNAs expression in Fig. 4. Table S12. GO term analysis of differentially expressed genes in RBS cells. Table S13. List of genes in NADs and their expression, from Fig. 6. (ZIP 1.78 MB) [file 12864_2015_2354_MOESM2_ESM.zip › Table S9-go_barplots_gomito_cluster1transcription.pdf]

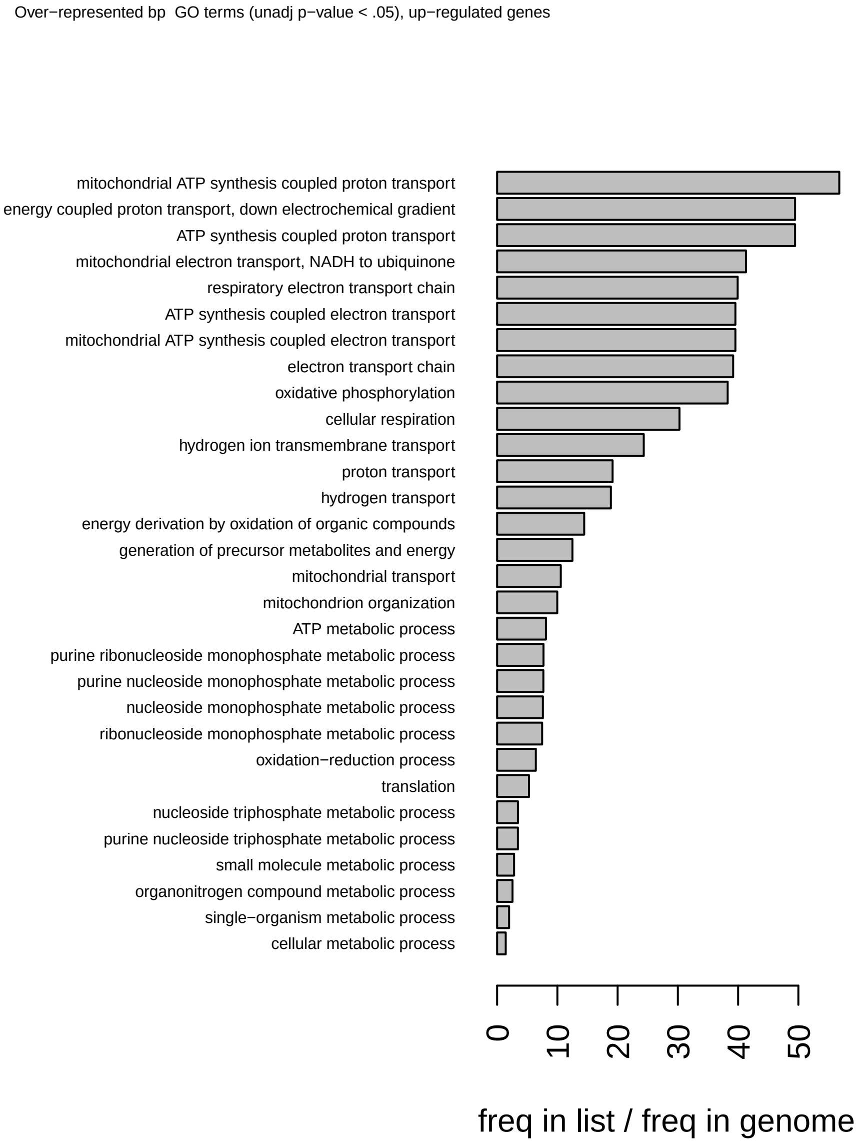

Over−represented bp GO terms (unadj p−value < .05), up−regulated genes
mitochondrial ATP synthesis coupled proton transport
energy coupled proton transport, down electrochemical gradient
ATP synthesis coupled proton transport
mitochondrial electron transport, NADH to ubiquinone
respiratory electron transport chain
ATP synthesis coupled electron transport
mitochondrial ATP synthesis coupled electron transport
electron transport chain
oxidative phosphorylation
cellular respiration
hydrogen ion transmembrane transport
proton transport
hydrogen transport
energy derivation by oxidation of organic compounds
generation of precursor metabolites and energy
mitochondrial transport
mitochondrion organization
ATP metabolic process
purine ribonucleoside monophosphate metabolic process
purine nucleoside monophosphate metabolic process
nucleoside monophosphate metabolic process
ribonucleoside monophosphate metabolic process
oxidation−reduction process
translation
nucleoside triphosphate metabolic process
purine nucleoside triphosphate metabolic process
small molecule metabolic process
organonitrogen compound metabolic process
single−organism metabolic process
cellular metabolic process
0
10
20
30
40
50
freq in list / freq in genome

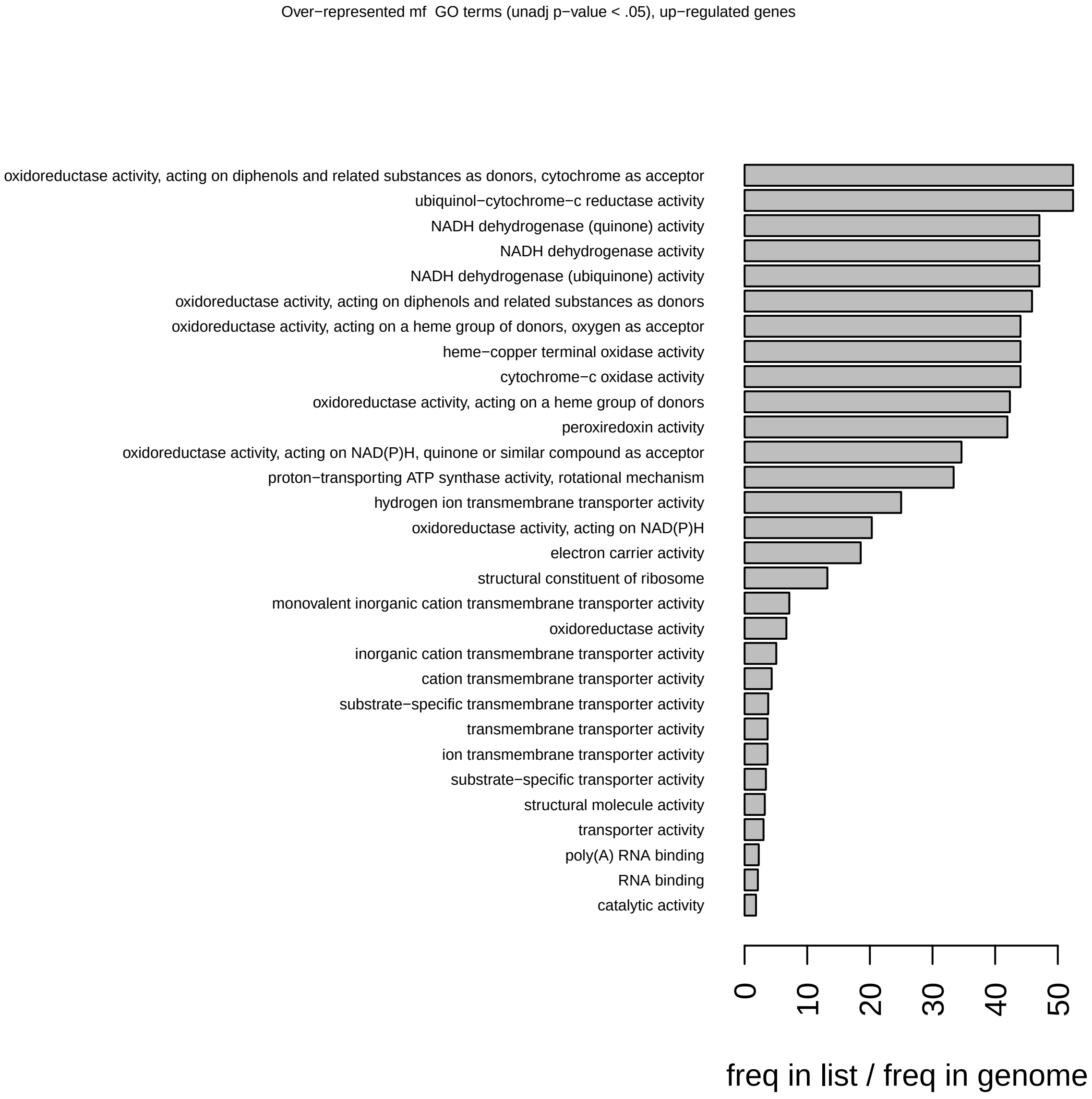
Over−represented mf GO terms (unadj p−value < .05), up−regulated genes
oxidoreductase activity, acting on diphenols and related substances as donors, cytochrome as acceptor
ubiquinol−cytochrome−c reductase activity
NADH dehydrogenase (quinone) activity
NADH dehydrogenase activity
NADH dehydrogenase (ubiquinone) activity
oxidoreductase activity, acting on diphenols and related substances as donors
oxidoreductase activity, acting on a heme group of donors, oxygen as acceptor
heme−copper terminal oxidase activity
cytochrome−c oxidase activity
oxidoreductase activity, acting on a heme group of donors
peroxiredoxin activity
oxidoreductase activity, acting on NAD(P)H, quinone or similar compound as acceptor
proton−transporting ATP synthase activity, rotational mechanism
hydrogen ion transmembrane transporter activity
oxidoreductase activity, acting on NAD(P)H
electron carrier activity
structural constituent of ribosome
monovalent inorganic cation transmembrane transporter activity
oxidoreductase activity
inorganic cation transmembrane transporter activity
cation transmembrane transporter activity
substrate−specific transmembrane transporter activity
transmembrane transporter activity
ion transmembrane transporter activity
substrate−specific transporter activity
structural molecule activity
transporter activity
poly(A) RNA binding
RNA binding
catalytic activity
0
10
20
30
40
50
freq in list / freq in genome

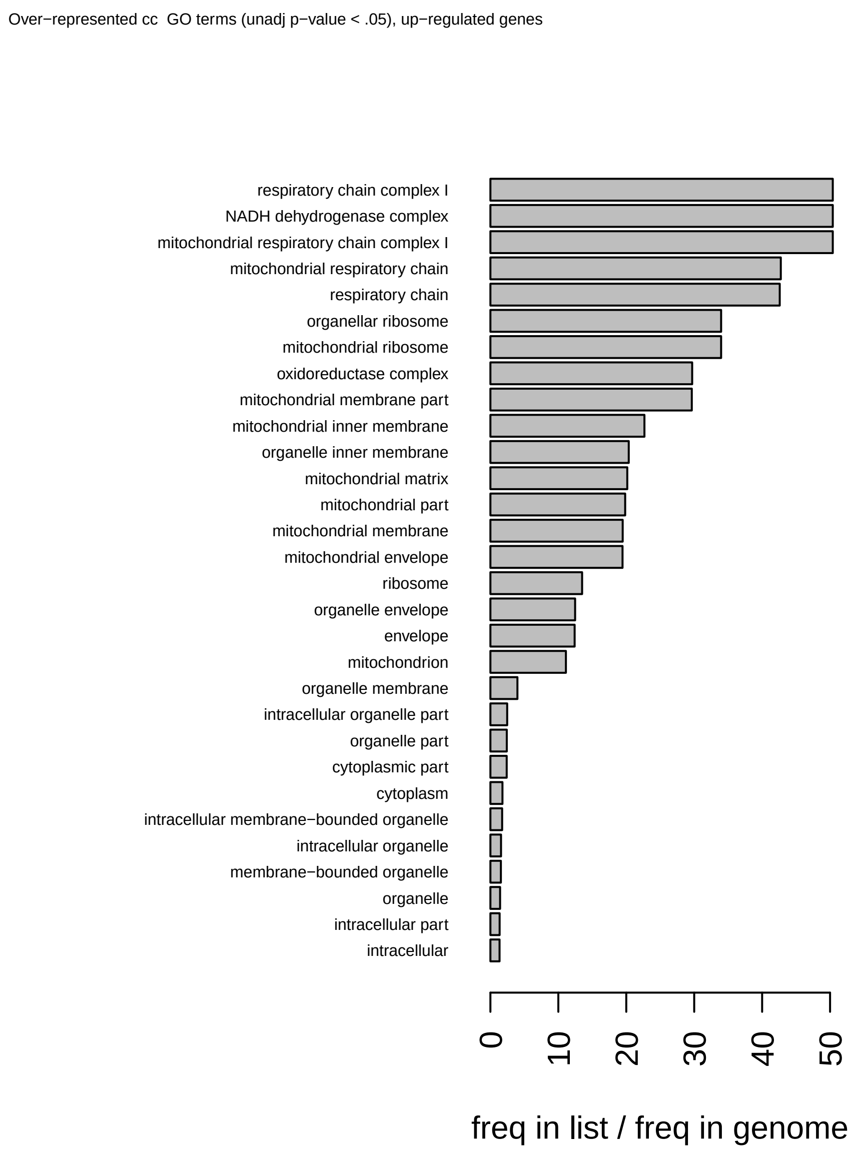

Over−represented cc GO terms (unadj p−value < .05), up−regulated genes
respiratory chain complex I
NADH dehydrogenase complex
mitochondrial respiratory chain complex I
mitochondrial respiratory chain
respiratory chain
organellar ribosome
mitochondrial ribosome
oxidoreductase complex
mitochondrial membrane part
mitochondrial inner membrane
organelle inner membrane
mitochondrial matrix
mitochondrial part
mitochondrial membrane
mitochondrial envelope
ribosome
organelle envelope
envelope
mitochondrion
organelle membrane
intracellular organelle part
organelle part
cytoplasmic part
cytoplasm
intracellular membrane−bounded organelle
intracellular organelle
membrane−bounded organelle
organelle
intracellular part
intracellular
0
10
20
30
40
50
freq in list / freq in genome
