# Supplementary material for: Improved transcription and translation with L-leucine stimulation of mTORC1 in Roberts syndrome
Source: BMC Genomics. 2016 Jan 5;17:25. doi: 10.1186/s12864-015-2354-y (PMC4700579; doi:10.1186/s12864-015-2354-y)
Supplement: Additional file 2: Table S1. — Summary table of Babel analysis. Table S2. GO term analysis of genes that show improved translational efficiency in RBS cells with L-leucine. Table S3. List for genes with fold change in translational efficiency greater than two and a total number of reads over 20 counts at 3 h. Table S4. List for genes with fold change in translational efficiency greater than two and a total number of reads over 20 counts at 24 h. Table S5. GO term analysis for genes with the new motif. Table S6. List of genes that have the new motif. Table S7. Summary table of data in boxplot of mTORC2-regulated gene show transcription and translation in Figure S5. Table S8. Summary table of data in heatmap for 868 mitochondrial genes—expression (Fig. 3) and translation. Table S9. GO term analysis for mitochondrial genes that show improve transcription with L-leucine. Table S10. GO term analysis for mitochondrial genes that improve translation with L-leucine. Table S11. Summary table of data in boxplot of snoRNAs expression in Fig. 4. Table S12. GO term analysis of differentially expressed genes in RBS cells. Table S13. List of genes in NADs and their expression, from Fig. 6. (ZIP 1.78 MB) [file 12864_2015_2354_MOESM2_ESM.zip › Table S6-Gene list of genes that have motif 5 from 3 hrs set.docx]

"TNFRSF25"

"PLOD1"

"C1orf64"

"MRPL37"

"BTBD8"

"AMY2B"

"GNRHR2"

"AL138795.1"

"LCE2A"

"LCE1E"

"RPS27"

"TSACC"

"SERTAD4-AS1"

"RPS7"

"RPS27A"

"POTEJ"

"RPL37A"

"SPP2"

"FBLN2"

"RPL15"

"CDHR4"

"RPL29"

"TKT"

"CCNI"

"TRIM60"

"RPL37"

"NSA2"

"RPS23"

"CAMLG"

"TGFBI"

"RPS14"

"THG1L"

"HIST1H4G"

"OR2B6"

"RPS18"

"RPS10"

"PGK2"

"EEF1A1"

"RPS12"

"PCLO"

"SND1"

"RPL30"

"PABPC1"

"ZNF572"

"EEF1D"

"RPL8"

"C9orf38"

"RPS6"

"FAM221B"

"KRT8P11"

"NR5A1"

"RPL12"

"C9orf16"

"LCN15"

"AGAP4"

"PDLIM1"

"AL158835.1"

"PRAP1"

"IPO7"

"RP11-716H6.1"

"CRTAM"

"GAPDH"

"AC021066.1"

"KRT81"

"KRT85"

"KRT18"

"EIF4B"

"LTA4H"

"MVK"

"RPL6"

"RPLP0"

"PABPC3"

"TPT1"

"HNRNPA1L2"

"IPO5"

"POTEM"

"RNASE10"

"IPO4"

"RPL10L"

"TBPL2"

"RP11-1085N6.3"

"AC026150.9"

"RP13-395E19.1"

"ACTC1"

"CHP1"

"RPS17"

"RPS17L"

"IDH2"

"RSL1D1"

"RPS15A"

"MT1F"

"RP11-680G10.1"

"CDT1"

"CD68"

"RP11-849F2.7"

"RPL26"

"TMEM97"

"EVI2A"

"NLE1"

"RPL23"

"FKBP10"

"ZNF385C"

"NAGLU"

"COL1A1"

"RGS9"

"ACTG1"

"RPL17"

"HSBP1L1"

"CNN2"

"BTBD2"

"STAP2"

"RPL36"

"CLPP"

"CCDC151"

"MAN2B1"

"RTBDN"

"DAND5"

"PGLS"

"RAB3A"

"RPSAP58"

"RPS4XP21"

"GPI"

"HSPB6"

"PPP1R14A"

"CLPTM1"

"GLTSCR2"

"KLK7"

"RPS9"

"LILRA5"

"NLRP2"

"EEF1A2"

"SIM2"

"RAC2"

"EIF3L"

"RPL3"

"PRPS2"

"RPS4X"

"RPL36A"

"RP1-164F3.9"

"GPRASP1"
