# Supplementary material for: Improved transcription and translation with L-leucine stimulation of mTORC1 in Roberts syndrome
Source: BMC Genomics. 2016 Jan 5;17:25. doi: 10.1186/s12864-015-2354-y (PMC4700579; doi:10.1186/s12864-015-2354-y)
Supplement: Additional file 2: Table S1. — Summary table of Babel analysis. Table S2. GO term analysis of genes that show improved translational efficiency in RBS cells with L-leucine. Table S3. List for genes with fold change in translational efficiency greater than two and a total number of reads over 20 counts at 3 h. Table S4. List for genes with fold change in translational efficiency greater than two and a total number of reads over 20 counts at 24 h. Table S5. GO term analysis for genes with the new motif. Table S6. List of genes that have the new motif. Table S7. Summary table of data in boxplot of mTORC2-regulated gene show transcription and translation in Figure S5. Table S8. Summary table of data in heatmap for 868 mitochondrial genes—expression (Fig. 3) and translation. Table S9. GO term analysis for mitochondrial genes that show improve transcription with L-leucine. Table S10. GO term analysis for mitochondrial genes that improve translation with L-leucine. Table S11. Summary table of data in boxplot of snoRNAs expression in Fig. 4. Table S12. GO term analysis of differentially expressed genes in RBS cells. Table S13. List of genes in NADs and their expression, from Fig. 6. (ZIP 1.78 MB) [file 12864_2015_2354_MOESM2_ESM.zip › Table S5-Go_barplots_motif_5.pdf]

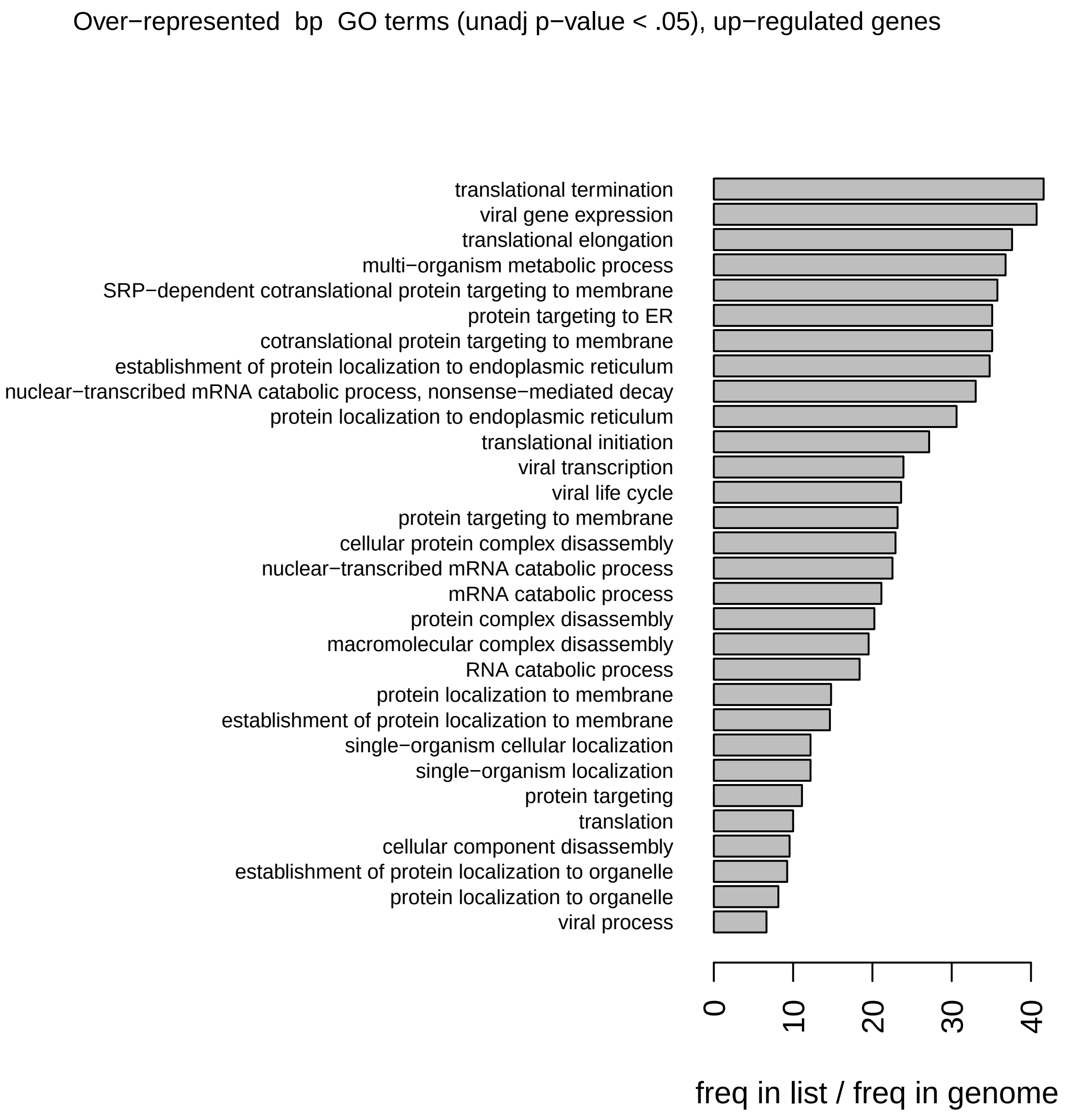
Over−represented bp GO terms (unadj p−value < .05), up−regulated genes
translational termination
viral gene expression
translational elongation
multi−organism metabolic process
SRP−dependent cotranslational protein targeting to membrane
protein targeting to ER
cotranslational protein targeting to membrane
establishment of protein localization to endoplasmic reticulum
nuclear−transcribed mRNA catabolic process, nonsense−mediated decay
protein localization to endoplasmic reticulum
translational initiation
viral transcription
viral life cycle
protein targeting to membrane
cellular protein complex disassembly
nuclear−transcribed mRNA catabolic process
mRNA catabolic process
protein complex disassembly
macromolecular complex disassembly
RNA catabolic process
protein localization to membrane
establishment of protein localization to membrane
single−organism cellular localization
single−organism localization
protein targeting
translation
cellular component disassembly
establishment of protein localization to organelle
protein localization to organelle
viral process
0
10
20
30
40
freq in list / freq in genome

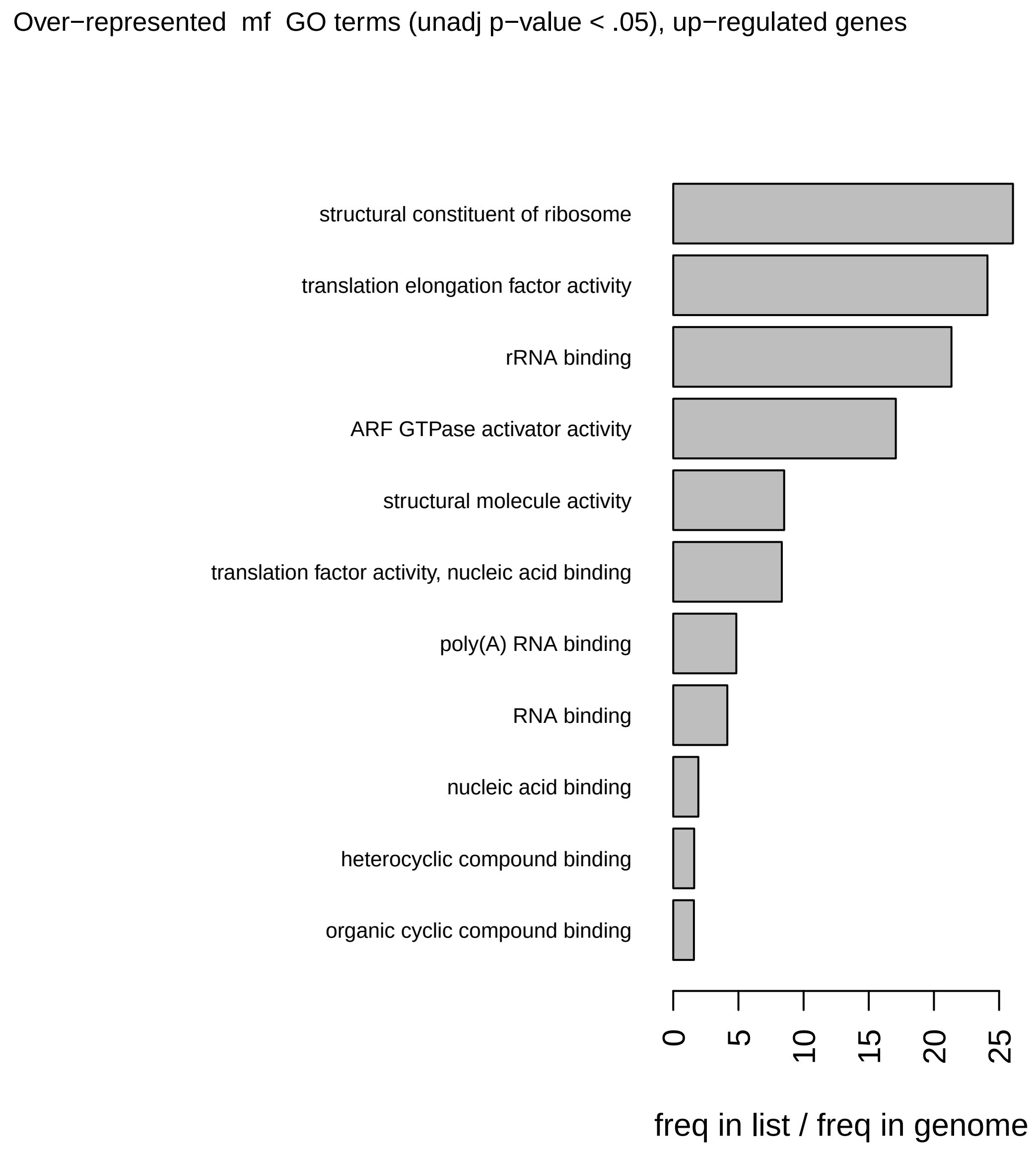
Over−represented mf GO terms (unadj p−value < .05), up−regulated genes
structural constituent of ribosome
translation elongation factor activity
rRNA binding
ARF GTPase activator activity
structural molecule activity
translation factor activity, nucleic acid binding
poly(A) RNA binding
RNA binding
nucleic acid binding
heterocyclic compound binding
organic cyclic compound binding
0
5
10
15
20
25
freq in list / freq in genome

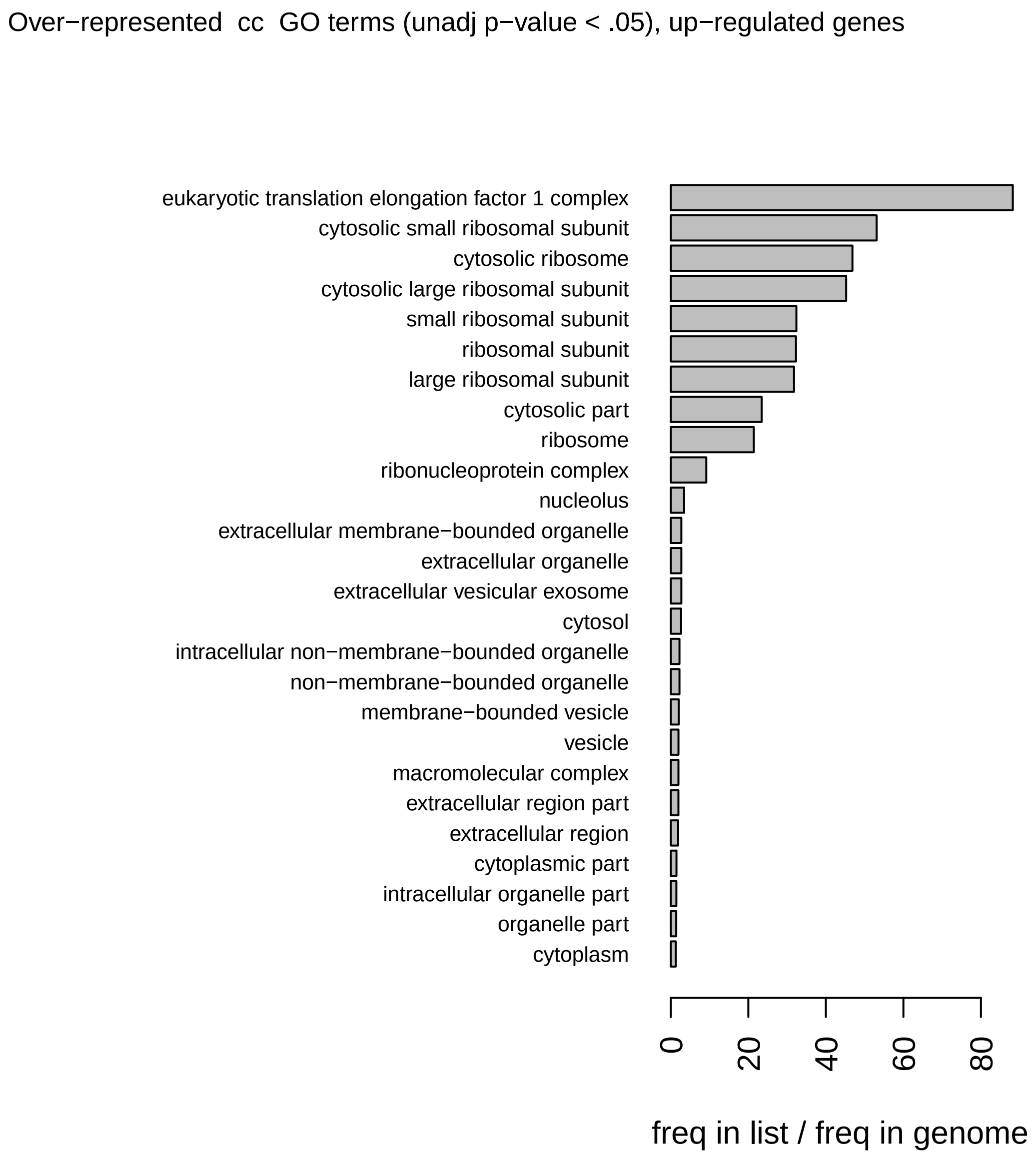

Over−represented cc GO terms (unadj p−value < .05), up−regulated genes
eukaryotic translation elongation factor 1 complex
cytosolic small ribosomal subunit
cytosolic ribosome
cytosolic large ribosomal subunit
small ribosomal subunit
ribosomal subunit
large ribosomal subunit
cytosolic part
ribosome
ribonucleoprotein complex
nucleolus
extracellular membrane−bounded organelle
extracellular organelle
extracellular vesicular exosome
cytosol
intracellular non−membrane−bounded organelle
non−membrane−bounded organelle
membrane−bounded vesicle
vesicle
macromolecular complex
extracellular region part
extracellular region
cytoplasmic part
intracellular organelle part
organelle part
cytoplasm
0
20
40
60
80
freq in list / freq in genome
