# Supplementary material for: Improved transcription and translation with L-leucine stimulation of mTORC1 in Roberts syndrome
Source: BMC Genomics. 2016 Jan 5;17:25. doi: 10.1186/s12864-015-2354-y (PMC4700579; doi:10.1186/s12864-015-2354-y)
Supplement: Additional file 2: Table S1. — Summary table of Babel analysis. Table S2. GO term analysis of genes that show improved translational efficiency in RBS cells with L-leucine. Table S3. List for genes with fold change in translational efficiency greater than two and a total number of reads over 20 counts at 3 h. Table S4. List for genes with fold change in translational efficiency greater than two and a total number of reads over 20 counts at 24 h. Table S5. GO term analysis for genes with the new motif. Table S6. List of genes that have the new motif. Table S7. Summary table of data in boxplot of mTORC2-regulated gene show transcription and translation in Figure S5. Table S8. Summary table of data in heatmap for 868 mitochondrial genes—expression (Fig. 3) and translation. Table S9. GO term analysis for mitochondrial genes that show improve transcription with L-leucine. Table S10. GO term analysis for mitochondrial genes that improve translation with L-leucine. Table S11. Summary table of data in boxplot of snoRNAs expression in Fig. 4. Table S12. GO term analysis of differentially expressed genes in RBS cells. Table S13. List of genes in NADs and their expression, from Fig. 6. (ZIP 1.78 MB) [file 12864_2015_2354_MOESM2_ESM.zip › Table S2-Go_barplots_translation effency for Mut-L vs Mut-D edg.pdf]

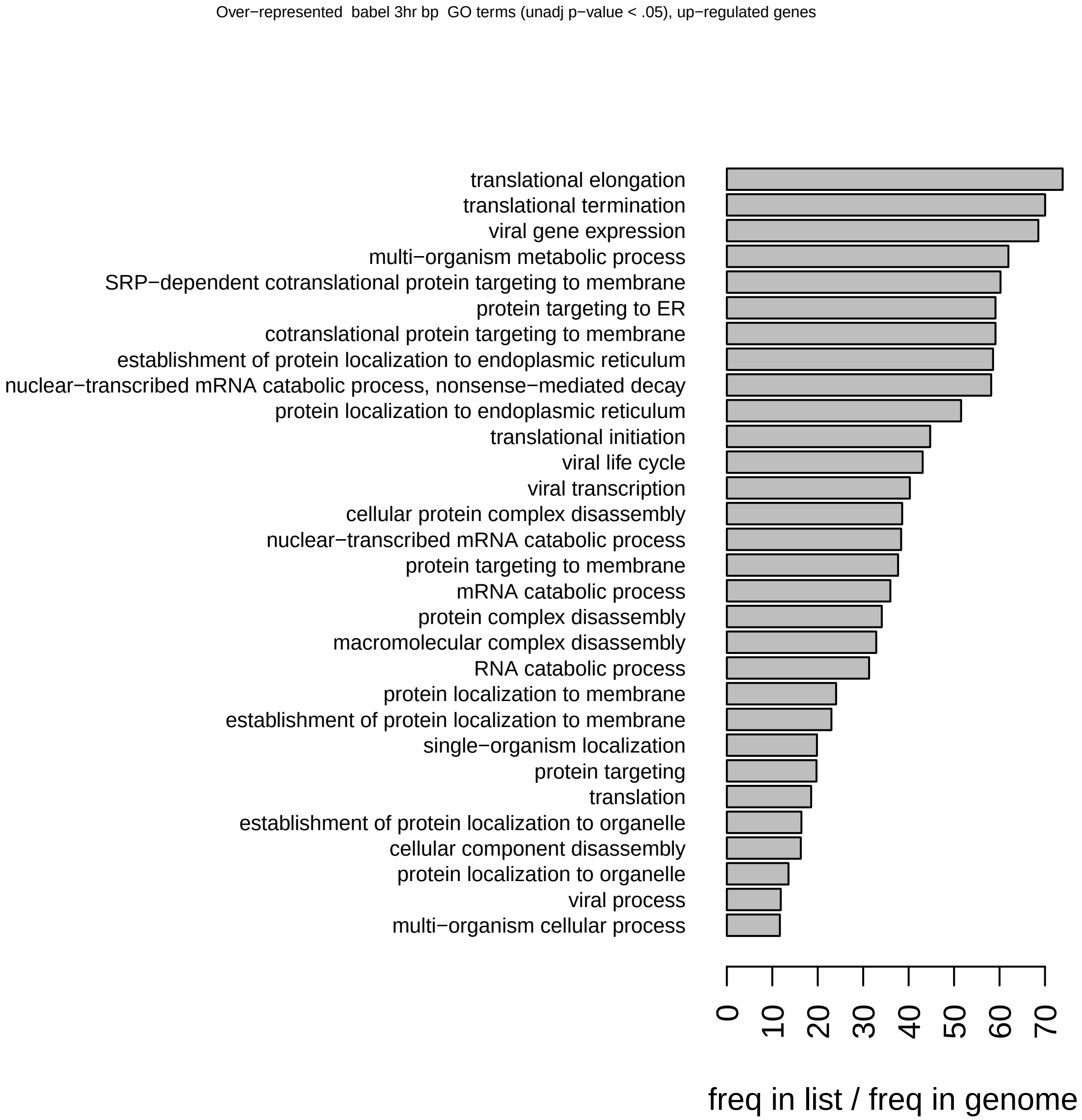
Over−represented babel 3hr bp GO terms (unadj p−value < .05), up−regulated genes
translational elongation
translational termination
viral gene expression
multi−organism metabolic process
SRP−dependent cotranslational protein targeting to membrane
protein targeting to ER
cotranslational protein targeting to membrane
establishment of protein localization to endoplasmic reticulum
nuclear−transcribed mRNA catabolic process, nonsense−mediated decay
protein localization to endoplasmic reticulum
translational initiation
viral life cycle
viral transcription
cellular protein complex disassembly
nuclear−transcribed mRNA catabolic process
protein targeting to membrane
mRNA catabolic process
protein complex disassembly
macromolecular complex disassembly
RNA catabolic process
protein localization to membrane
establishment of protein localization to membrane
single−organism localization
protein targeting
translation
establishment of protein localization to organelle
cellular component disassembly
protein localization to organelle
viral process
multi−organism cellular process
0
10
20
30
40
50
60
70
freq in list / freq in genome

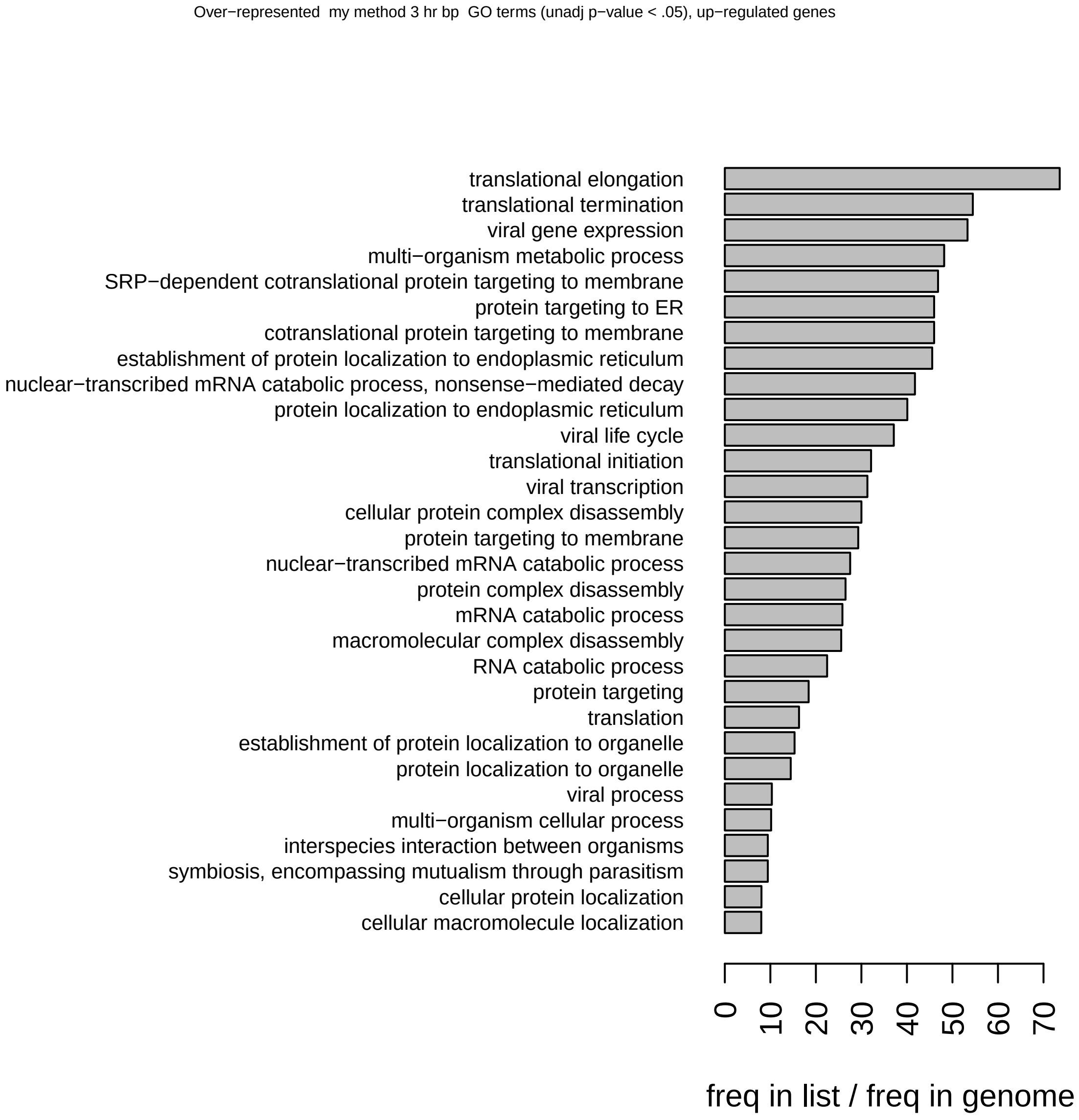
Over−represented my method 3 hr bp GO terms (unadj p−value < .05), up−regulated genes
translational elongation
translational termination
viral gene expression
multi−organism metabolic process
SRP−dependent cotranslational protein targeting to membrane
protein targeting to ER
cotranslational protein targeting to membrane
establishment of protein localization to endoplasmic reticulum
nuclear−transcribed mRNA catabolic process, nonsense−mediated decay
protein localization to endoplasmic reticulum
viral life cycle
translational initiation
viral transcription
cellular protein complex disassembly
protein targeting to membrane
nuclear−transcribed mRNA catabolic process
protein complex disassembly
mRNA catabolic process
macromolecular complex disassembly
RNA catabolic process
protein targeting
translation
establishment of protein localization to organelle
protein localization to organelle
viral process
multi−organism cellular process
interspecies interaction between organisms
symbiosis, encompassing mutualism through parasitism
cellular protein localization
cellular macromolecule localization
0
10
20
30
40
50
60
70
freq in list / freq in genome

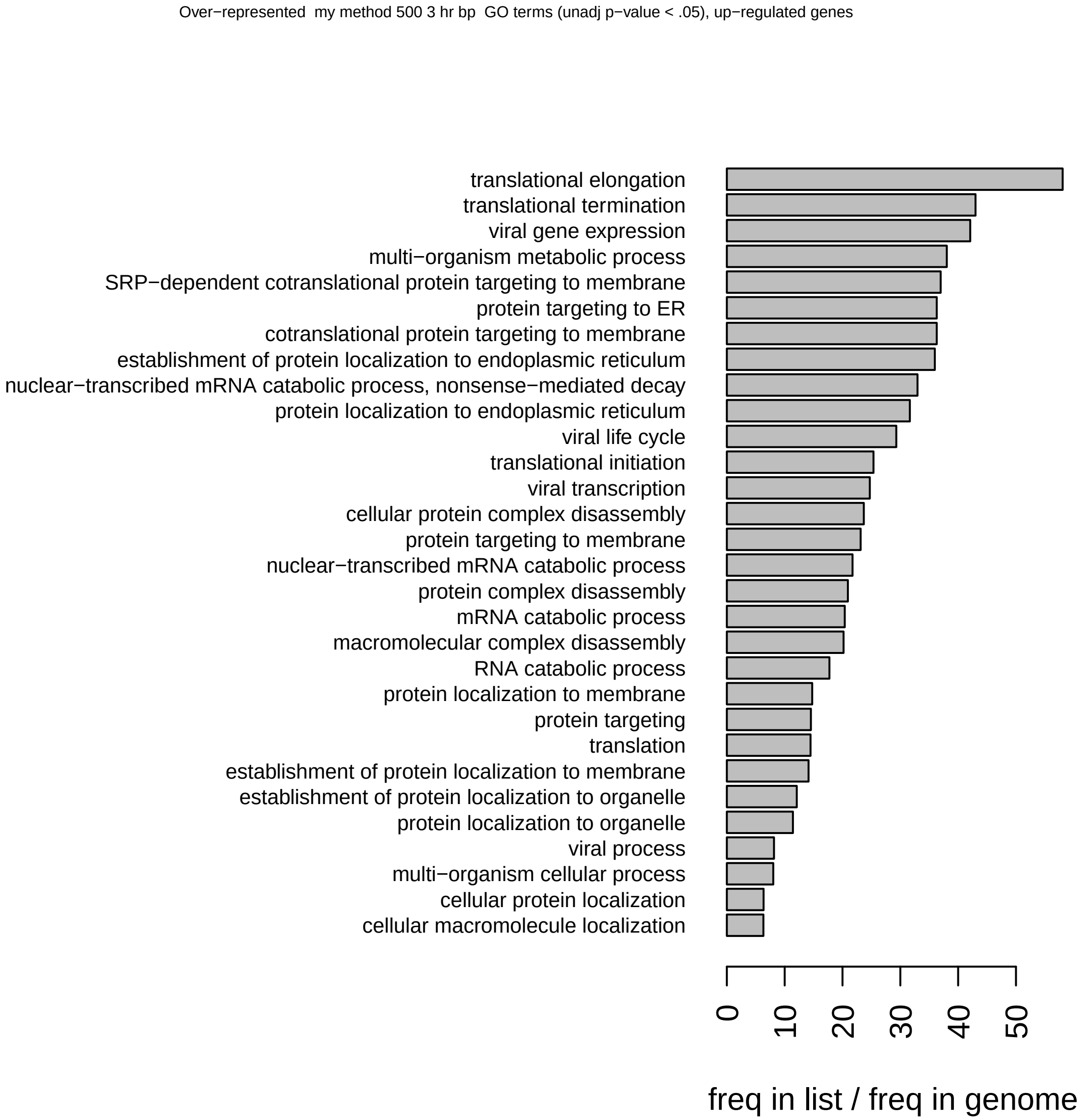

Over−represented my method 500 3 hr bp GO terms (unadj p−value < .05), up−regulated genes
translational elongation
translational termination
viral gene expression
multi−organism metabolic process
SRP−dependent cotranslational protein targeting to membrane
protein targeting to ER
cotranslational protein targeting to membrane
establishment of protein localization to endoplasmic reticulum
nuclear−transcribed mRNA catabolic process, nonsense−mediated decay
protein localization to endoplasmic reticulum
viral life cycle
translational initiation
viral transcription
cellular protein complex disassembly
protein targeting to membrane
nuclear−transcribed mRNA catabolic process
protein complex disassembly
mRNA catabolic process
macromolecular complex disassembly
RNA catabolic process
protein localization to membrane
protein targeting
translation
establishment of protein localization to membrane
establishment of protein localization to organelle
protein localization to organelle
viral process
multi−organism cellular process
cellular protein localization
cellular macromolecule localization
0
10
20
30
40
50
freq in list / freq in genome

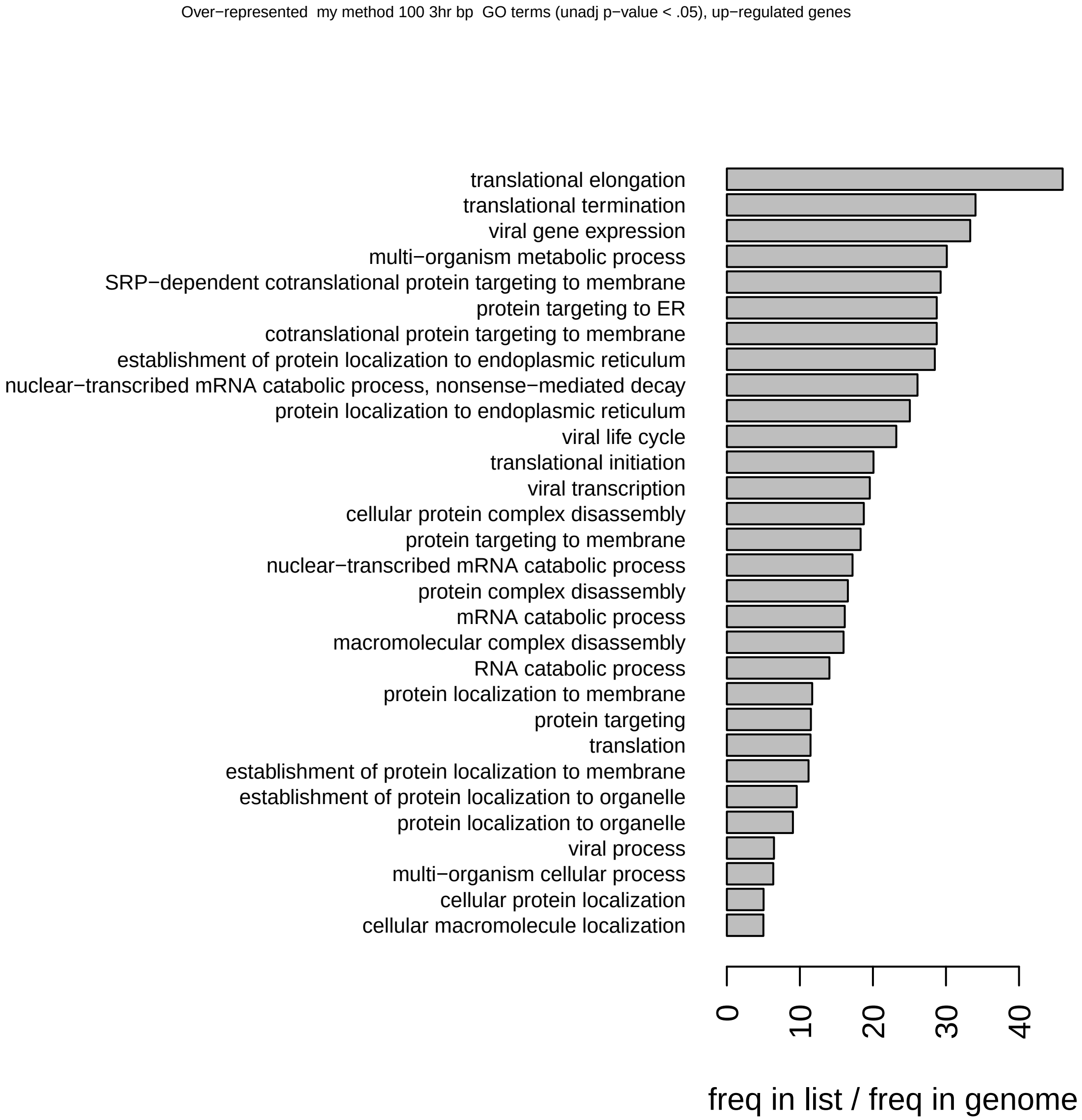
Over−represented my method 100 3hr bp GO terms (unadj p−value < .05), up−regulated genes
translational elongation
translational termination
viral gene expression
multi−organism metabolic process
SRP−dependent cotranslational protein targeting to membrane
protein targeting to ER
cotranslational protein targeting to membrane
establishment of protein localization to endoplasmic reticulum
nuclear−transcribed mRNA catabolic process, nonsense−mediated decay
protein localization to endoplasmic reticulum
viral life cycle
translational initiation
viral transcription
cellular protein complex disassembly
protein targeting to membrane
nuclear−transcribed mRNA catabolic process
protein complex disassembly
mRNA catabolic process
macromolecular complex disassembly
RNA catabolic process
protein localization to membrane
protein targeting
translation
establishment of protein localization to membrane
establishment of protein localization to organelle
protein localization to organelle
viral process
multi−organism cellular process
cellular protein localization
cellular macromolecule localization
0
10
20
30
40
freq in list / freq in genome

Over−represented my method 20 3hr bp GO terms (unadj p−value < .05), up−regulated genes

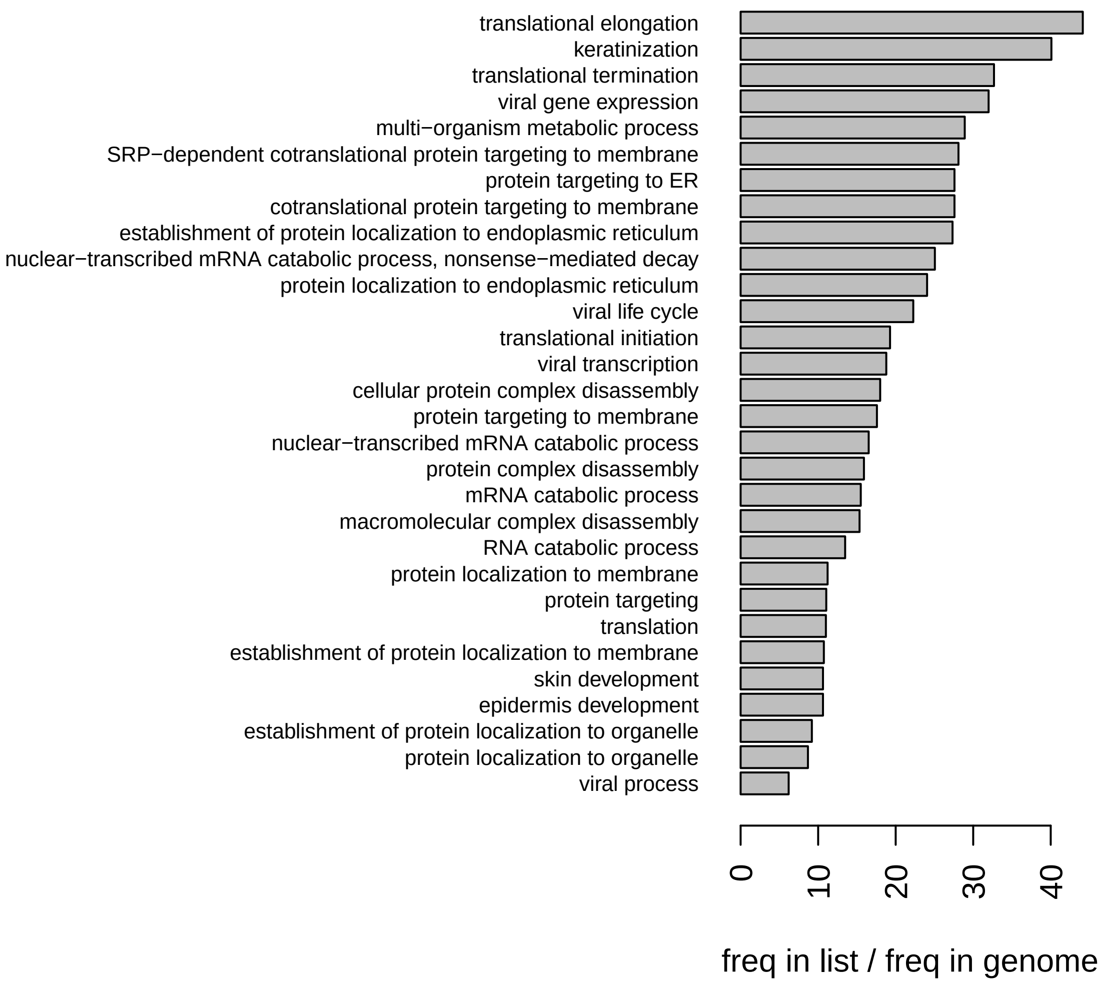

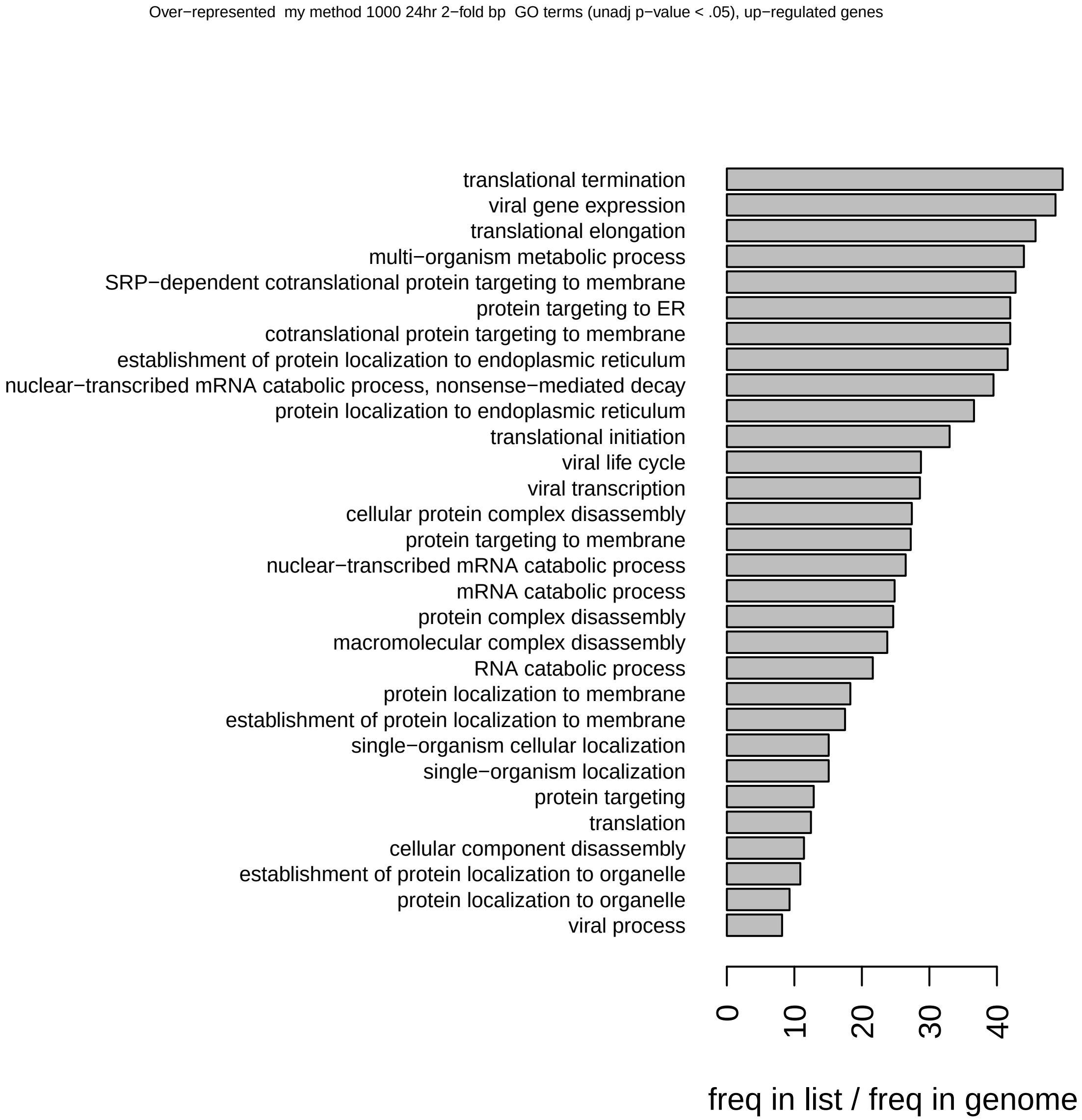
Over−represented my method 1000 24hr 2−fold bp GO terms (unadj p−value < .05), up−regulated genes
translational termination
viral gene expression
translational elongation
multi−organism metabolic process
SRP−dependent cotranslational protein targeting to membrane
protein targeting to ER
cotranslational protein targeting to membrane
establishment of protein localization to endoplasmic reticulum
nuclear−transcribed mRNA catabolic process, nonsense−mediated decay
protein localization to endoplasmic reticulum
translational initiation
viral life cycle
viral transcription
cellular protein complex disassembly
protein targeting to membrane
nuclear−transcribed mRNA catabolic process
mRNA catabolic process
protein complex disassembly
macromolecular complex disassembly
RNA catabolic process
protein localization to membrane
establishment of protein localization to membrane
single−organism cellular localization
single−organism localization
protein targeting
translation
cellular component disassembly
establishment of protein localization to organelle
protein localization to organelle
viral process
0
10
20
30
40
freq in list / freq in genome

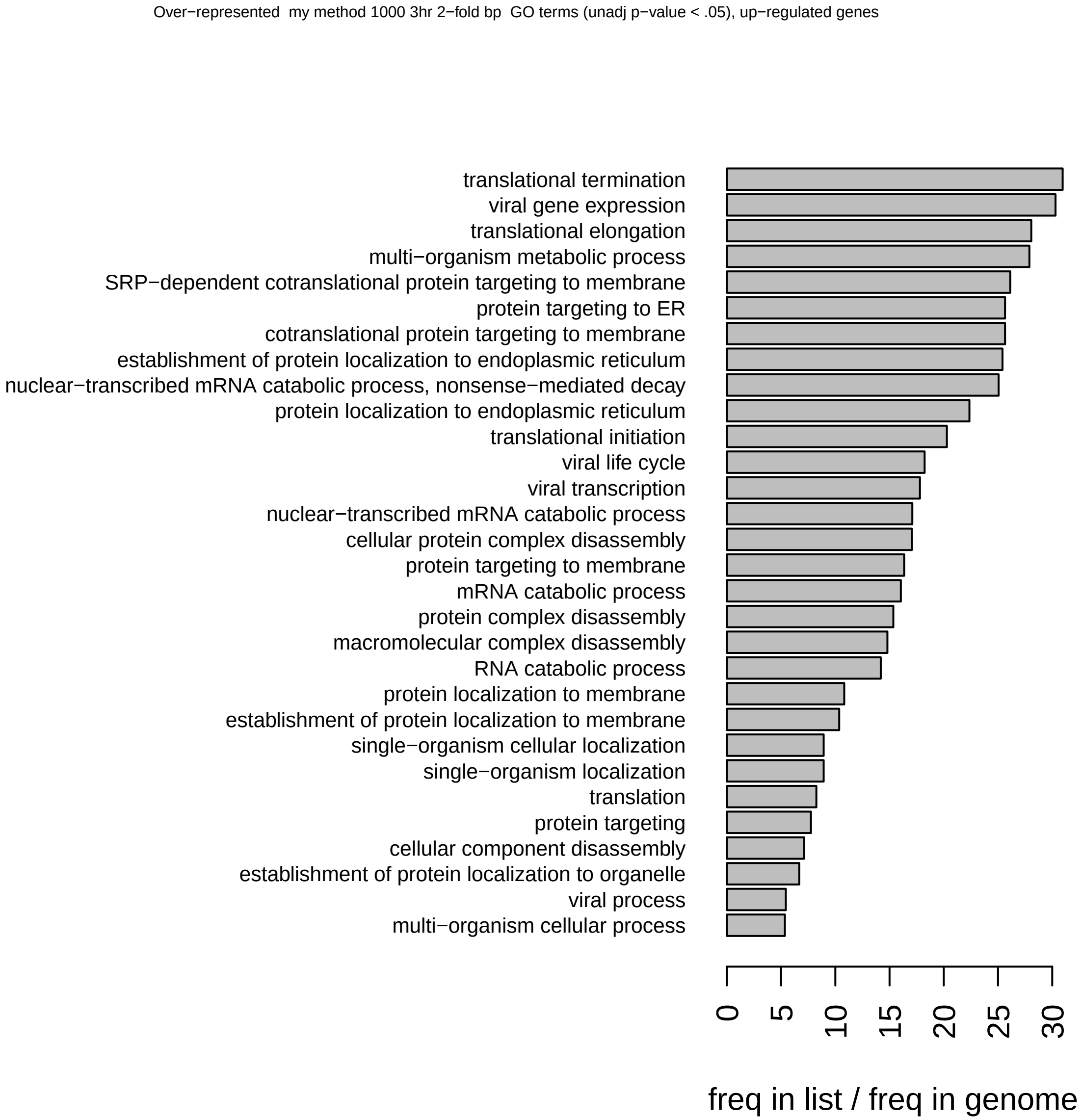
Over−represented my method 1000 3hr 2−fold bp GO terms (unadj p−value < .05), up−regulated genes
translational termination
viral gene expression
translational elongation
multi−organism metabolic process
SRP−dependent cotranslational protein targeting to membrane
protein targeting to ER
cotranslational protein targeting to membrane
establishment of protein localization to endoplasmic reticulum
nuclear−transcribed mRNA catabolic process, nonsense−mediated decay
protein localization to endoplasmic reticulum
translational initiation
viral life cycle
viral transcription
nuclear−transcribed mRNA catabolic process
cellular protein complex disassembly
protein targeting to membrane
mRNA catabolic process
protein complex disassembly
macromolecular complex disassembly
RNA catabolic process
protein localization to membrane
establishment of protein localization to membrane
single−organism cellular localization
single−organism localization
translation
protein targeting
cellular component disassembly
establishment of protein localization to organelle
viral process
multi−organism cellular process
0
5
10
15
20
25
30
freq in list / freq in genome

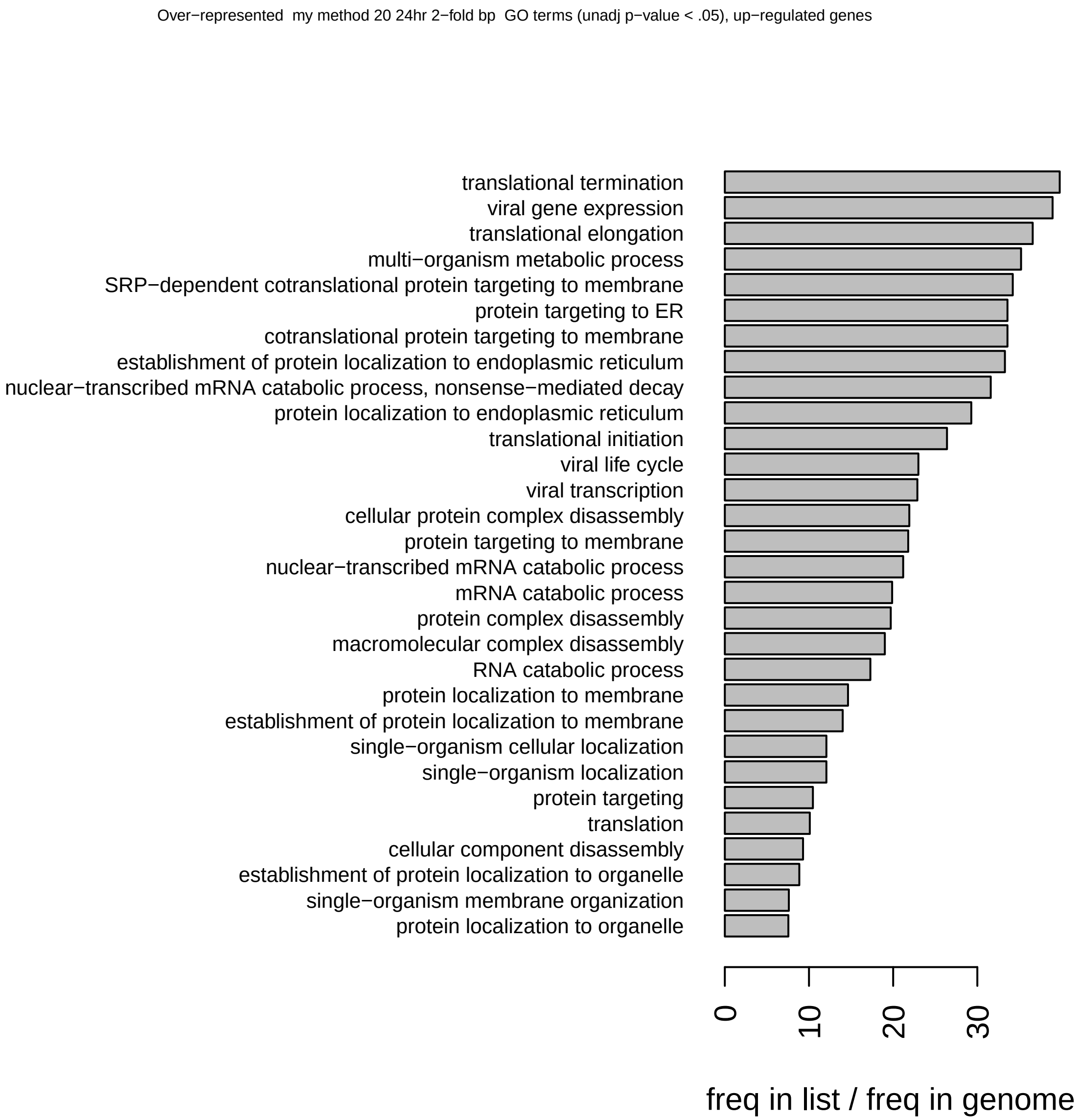
Over−represented my method 20 24hr 2−fold bp GO terms (unadj p−value < .05), up−regulated genes
translational termination
viral gene expression
translational elongation
multi−organism metabolic process
SRP−dependent cotranslational protein targeting to membrane
protein targeting to ER
cotranslational protein targeting to membrane
establishment of protein localization to endoplasmic reticulum
nuclear−transcribed mRNA catabolic process, nonsense−mediated decay
protein localization to endoplasmic reticulum
translational initiation
viral life cycle
viral transcription
cellular protein complex disassembly
protein targeting to membrane
nuclear−transcribed mRNA catabolic process
mRNA catabolic process
protein complex disassembly
macromolecular complex disassembly
RNA catabolic process
protein localization to membrane
establishment of protein localization to membrane
single−organism cellular localization
single−organism localization
protein targeting
translation
cellular component disassembly
establishment of protein localization to organelle
single−organism membrane organization
protein localization to organelle
0
10
20
30
freq in list / freq in genome

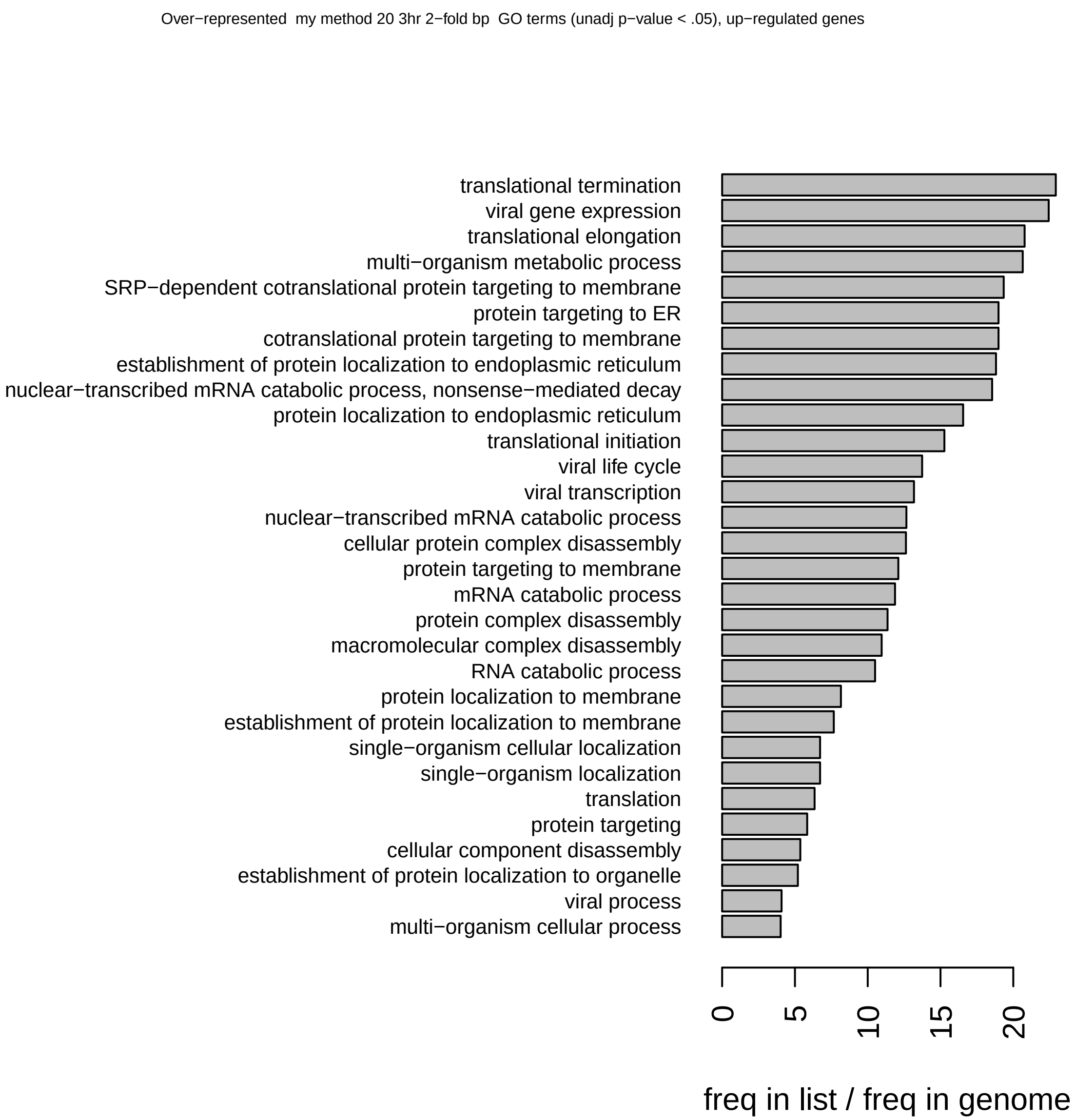
Over−represented my method 20 3hr 2−fold bp GO terms (unadj p−value < .05), up−regulated genes
translational termination
viral gene expression
translational elongation
multi−organism metabolic process
SRP−dependent cotranslational protein targeting to membrane
protein targeting to ER
cotranslational protein targeting to membrane
establishment of protein localization to endoplasmic reticulum
nuclear−transcribed mRNA catabolic process, nonsense−mediated decay
protein localization to endoplasmic reticulum
translational initiation
viral life cycle
viral transcription
nuclear−transcribed mRNA catabolic process
cellular protein complex disassembly
protein targeting to membrane
mRNA catabolic process
protein complex disassembly
macromolecular complex disassembly
RNA catabolic process
protein localization to membrane
establishment of protein localization to membrane
single−organism cellular localization
single−organism localization
translation
protein targeting
cellular component disassembly
establishment of protein localization to organelle
viral process
multi−organism cellular process
0
5
10
15
20
freq in list / freq in genome

Over−represented babel 3hr mf GO terms (unadj p−value < .05), up−regulated genes

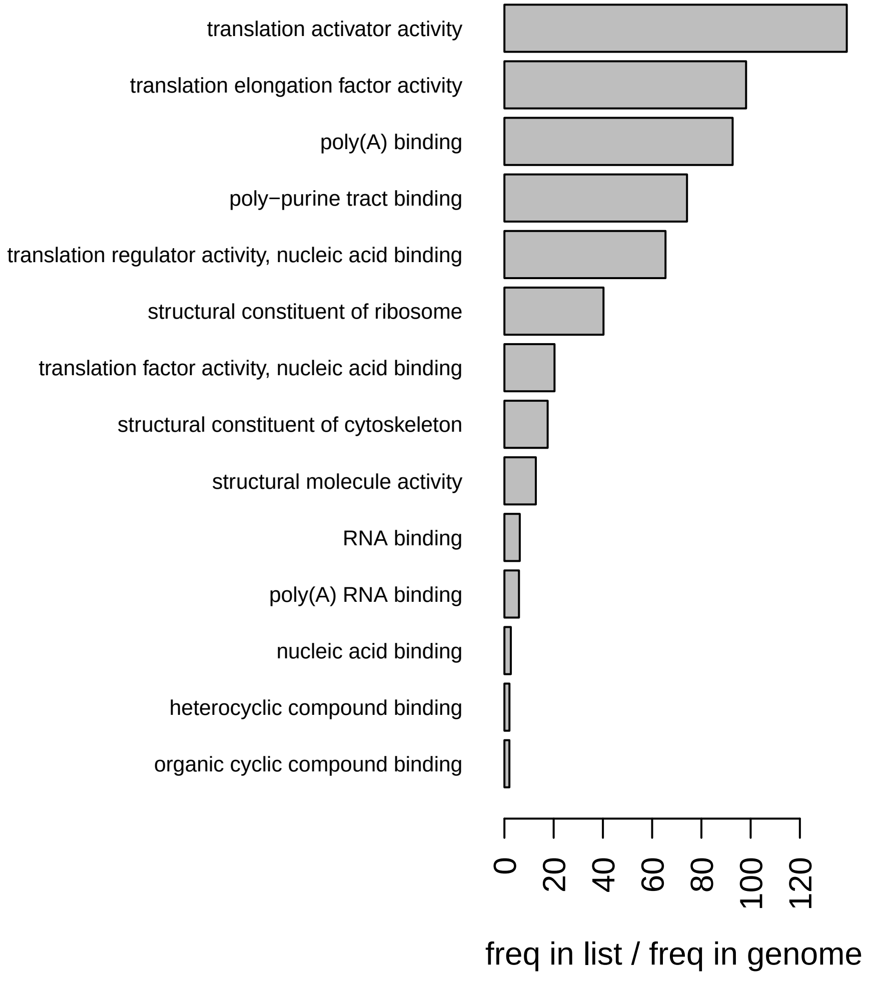

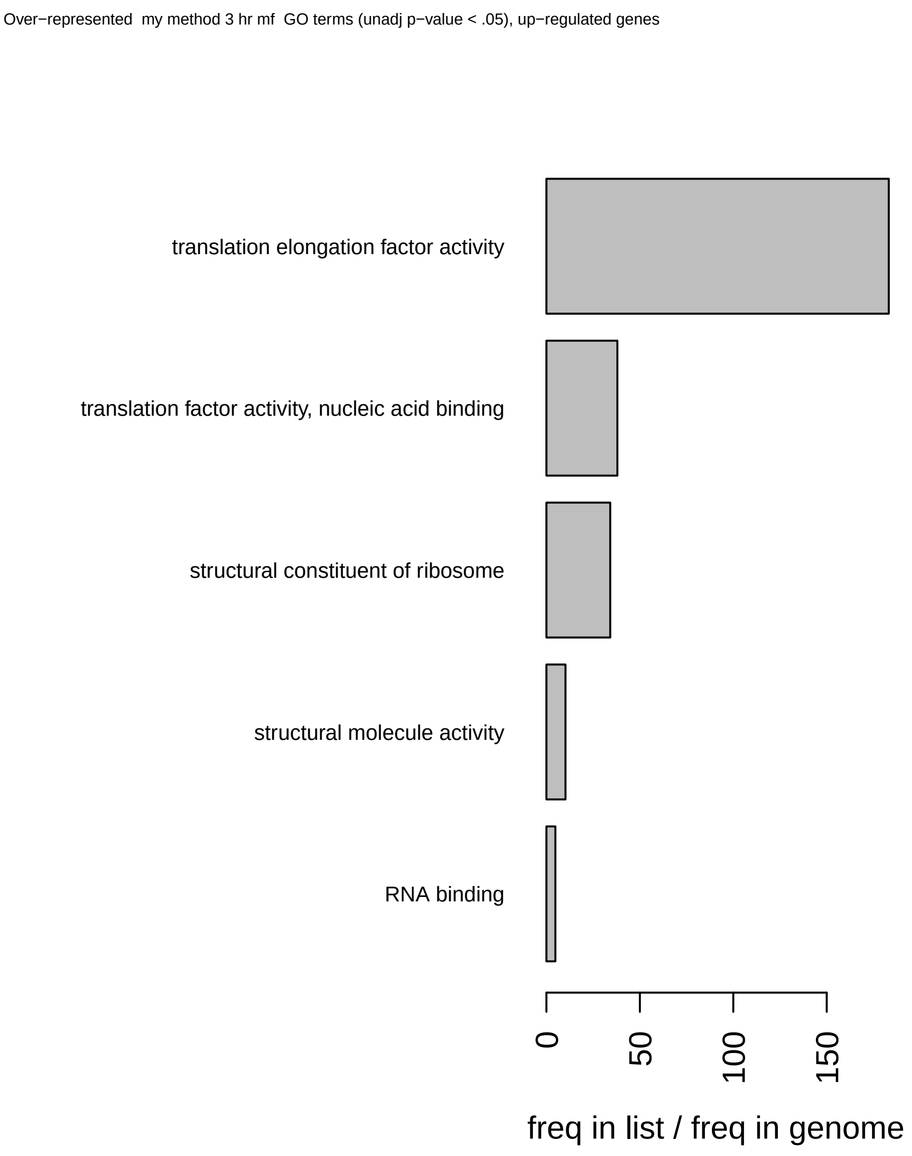
Over−represented my method 3 hr mf GO terms (unadj p−value < .05), up−regulated genes
translation elongation factor activity
translation factor activity, nucleic acid binding
structural constituent of ribosome
structural molecule activity
RNA binding
0
50
100
150
freq in list / freq in genome

Over−represented my method 500 3 hr mf GO terms (unadj p−value < .05), up−regulated genes

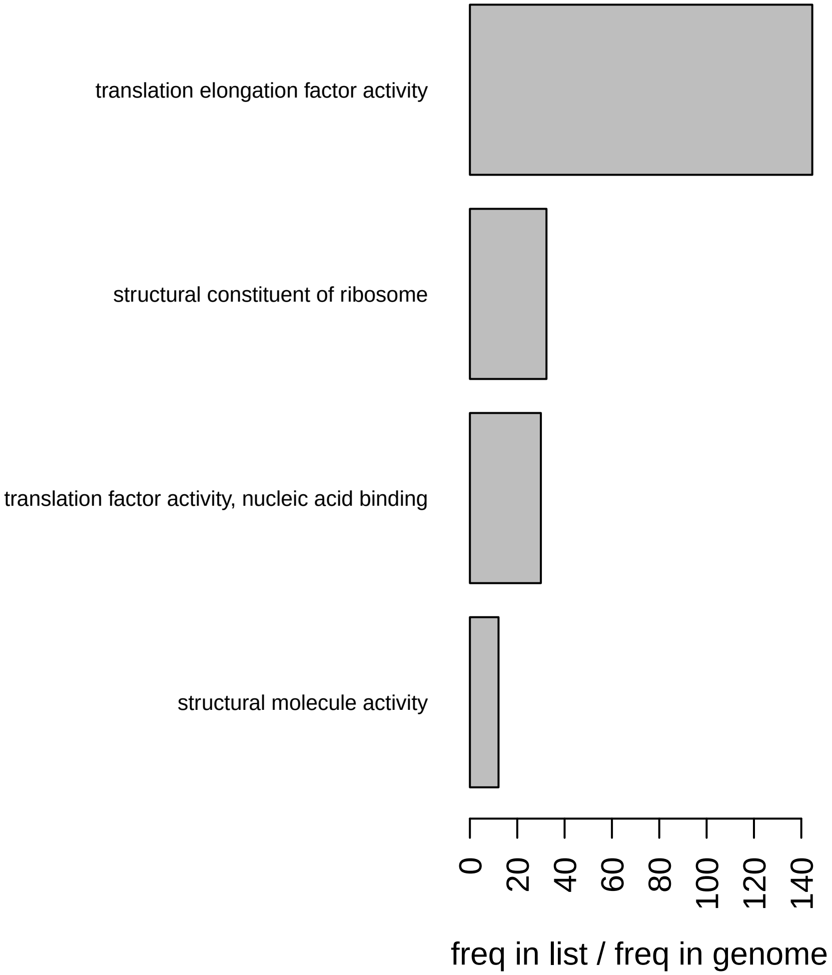

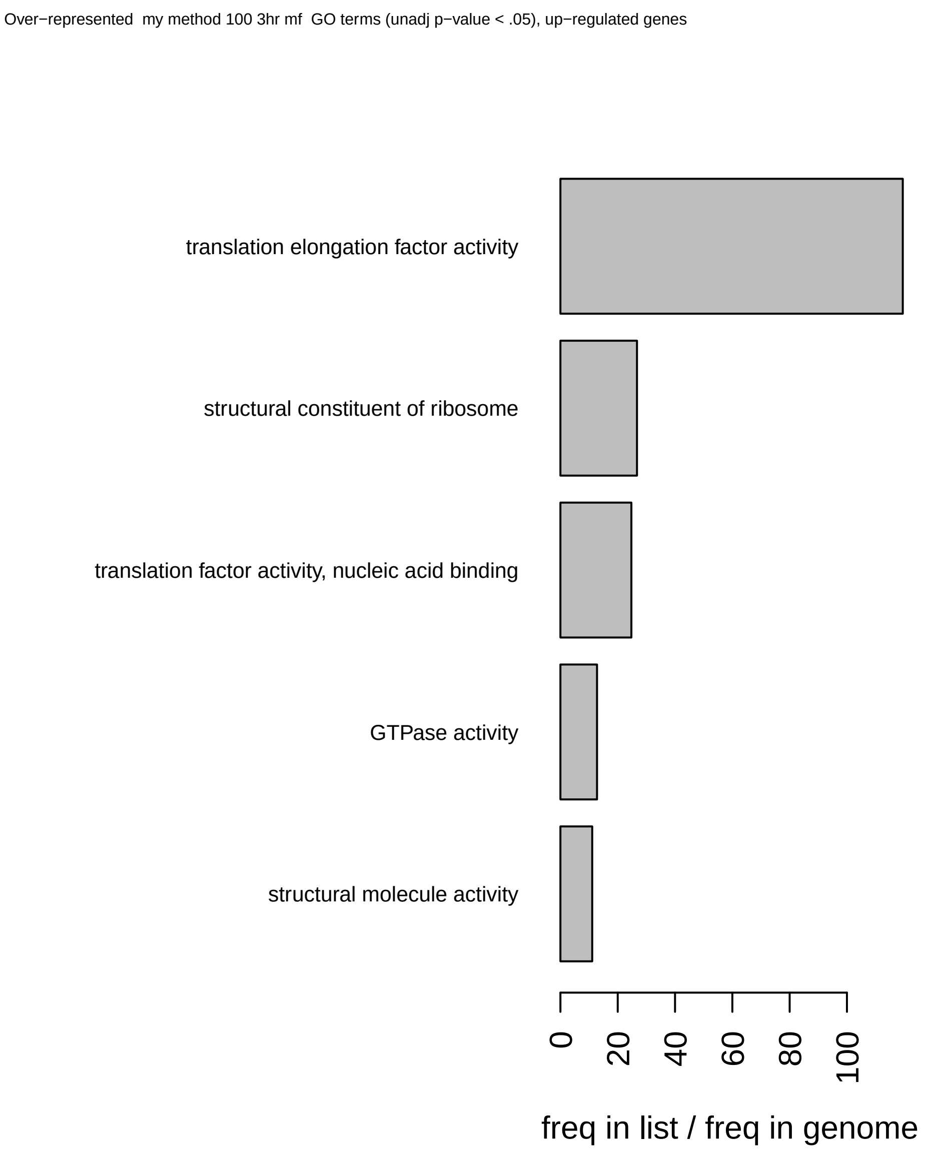
Over−represented my method 100 3hr mf GO terms (unadj p−value < .05), up−regulated genes
translation elongation factor activity
structural constituent of ribosome
translation factor activity, nucleic acid binding
GTPase activity
structural molecule activity
0
20
40
60
80
100
freq in list / freq in genome

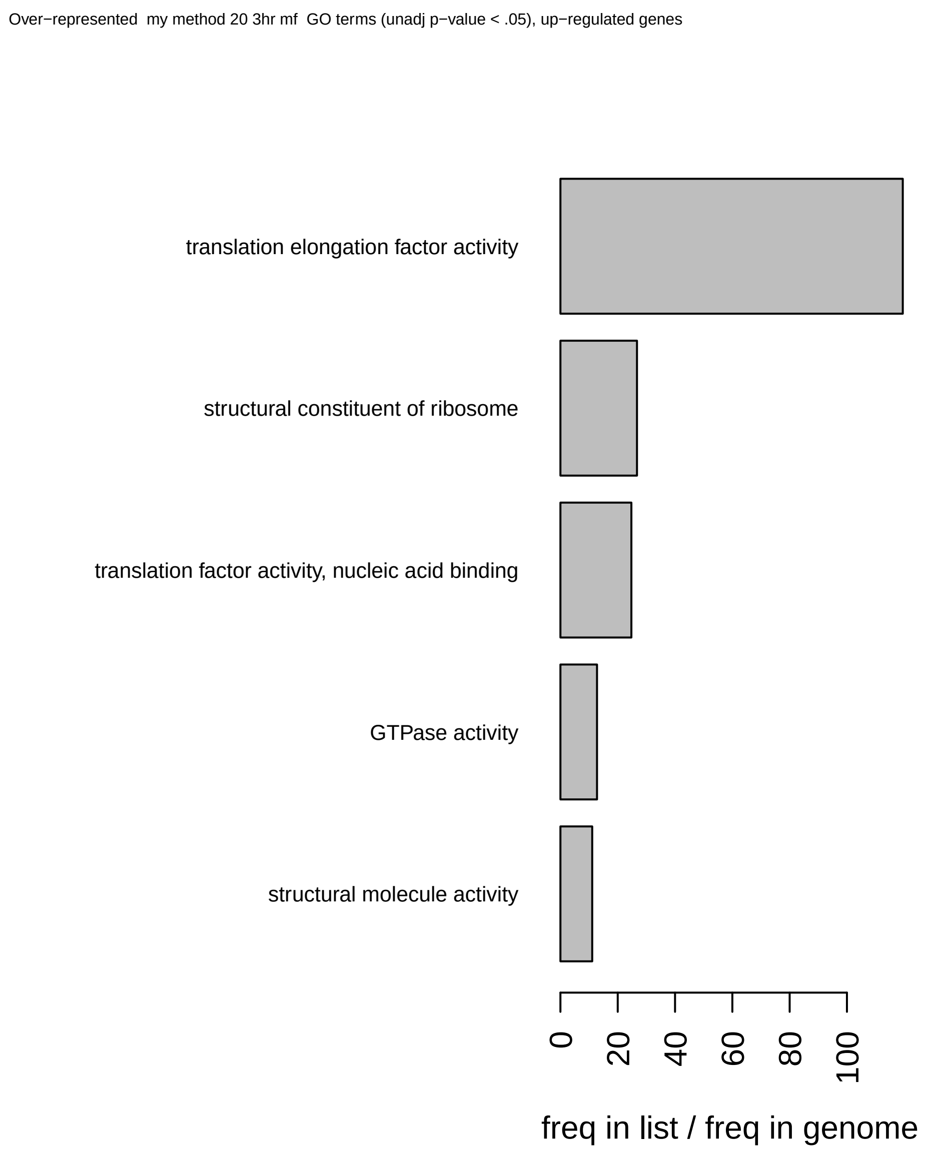

Over−represented my method 20 3hr mf GO terms (unadj p−value < .05), up−regulated genes
translation elongation factor activity
structural constituent of ribosome
translation factor activity, nucleic acid binding
GTPase activity
structural molecule activity
0
20
40
60
80
100
freq in list / freq in genome

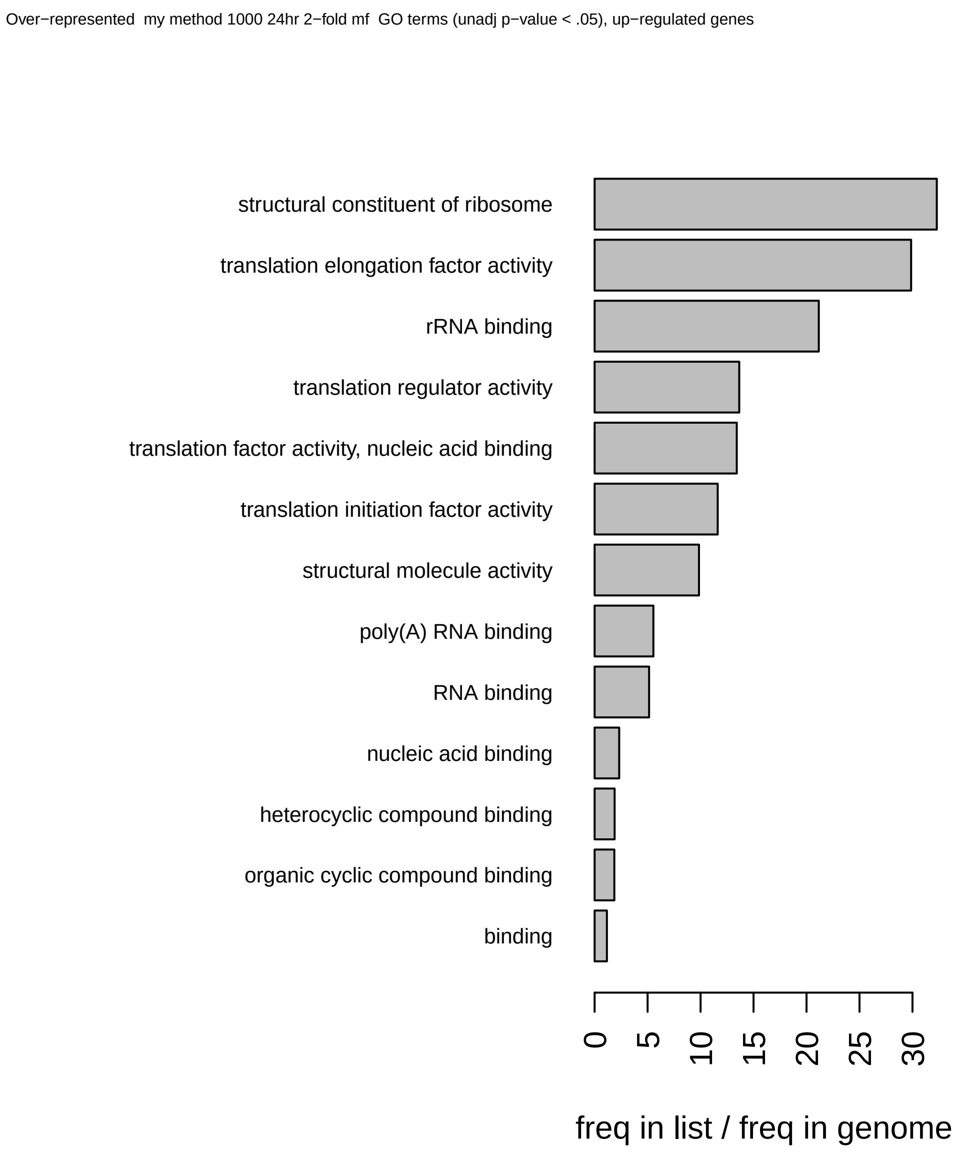
Over−represented my method 1000 24hr 2−fold mf GO terms (unadj p−value < .05), up−regulated genes
structural constituent of ribosome
translation elongation factor activity
rRNA binding
translation regulator activity
translation factor activity, nucleic acid binding
translation initiation factor activity
structural molecule activity
poly(A) RNA binding
RNA binding
nucleic acid binding
heterocyclic compound binding
organic cyclic compound binding
binding
0
5
10
15
20
25
30
freq in list / freq in genome

Over−represented my method 1000 3hr 2−fold mf GO terms (unadj p−value < .05), up−regulated genes

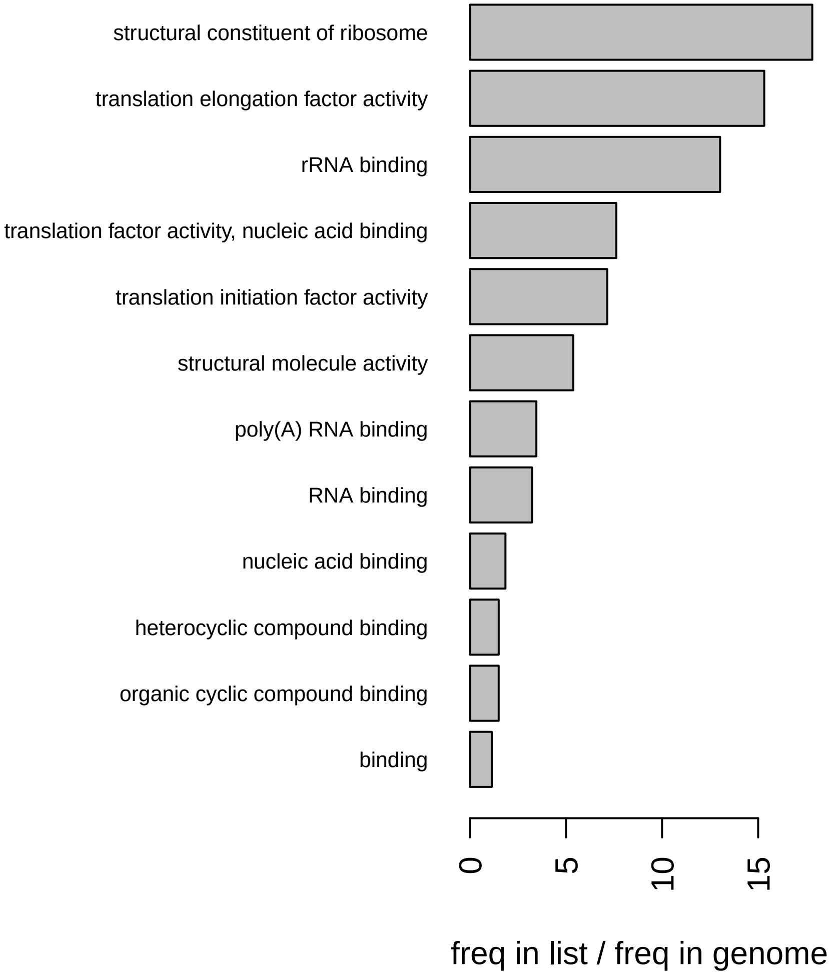

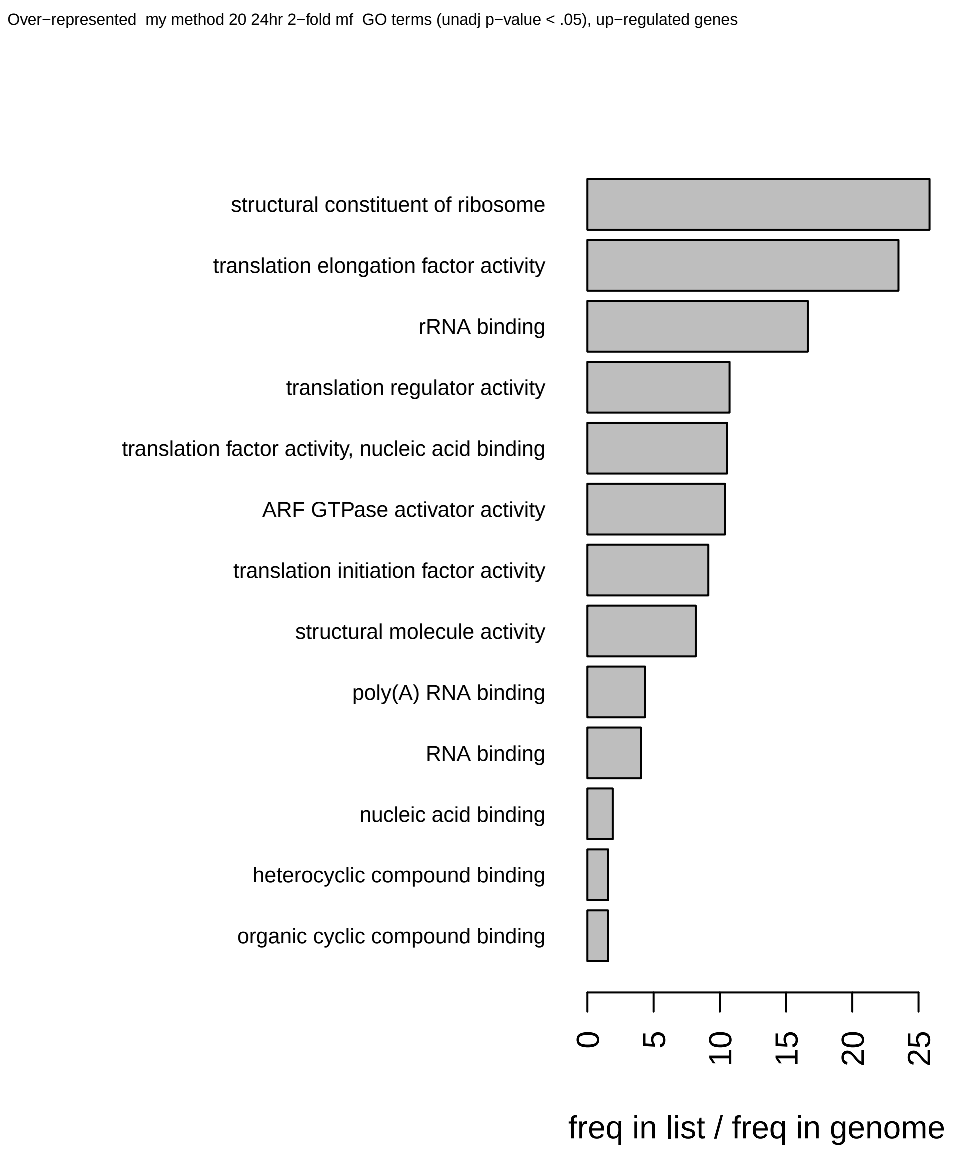
Over−represented my method 20 24hr 2−fold mf GO terms (unadj p−value < .05), up−regulated genes
structural constituent of ribosome
translation elongation factor activity
rRNA binding
translation regulator activity
translation factor activity, nucleic acid binding
ARF GTPase activator activity
translation initiation factor activity
structural molecule activity
poly(A) RNA binding
RNA binding
nucleic acid binding
heterocyclic compound binding
organic cyclic compound binding
0
5
10
15
20
25
freq in list / freq in genome

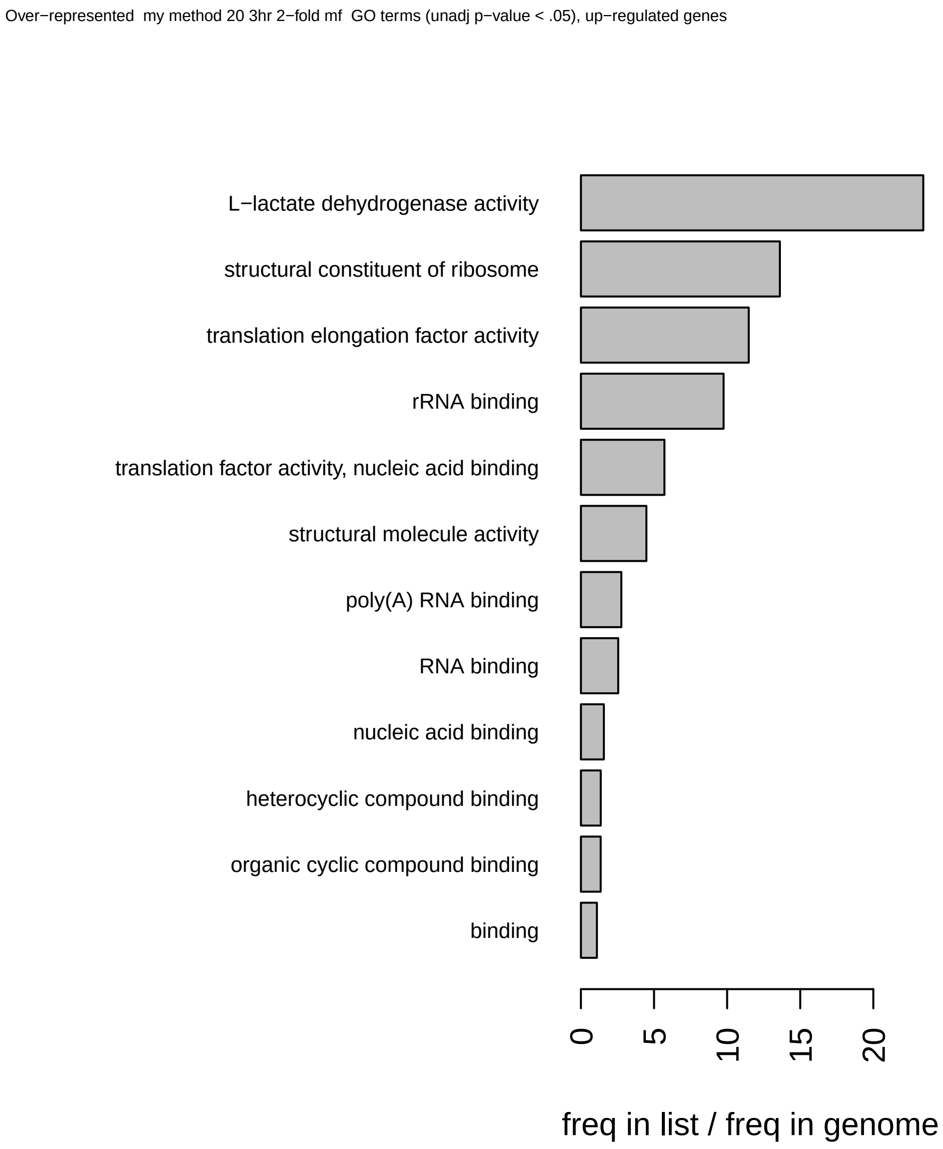
Over−represented my method 20 3hr 2−fold mf GO terms (unadj p−value < .05), up−regulated genes
L−lactate dehydrogenase activity
structural constituent of ribosome
translation elongation factor activity
rRNA binding
translation factor activity, nucleic acid binding
structural molecule activity
poly(A) RNA binding
RNA binding
nucleic acid binding
heterocyclic compound binding
organic cyclic compound binding
binding
0
5
10
15
20
freq in list / freq in genome

Over−represented babel 3hr cc GO terms (unadj p−value < .05), up−regulated genes

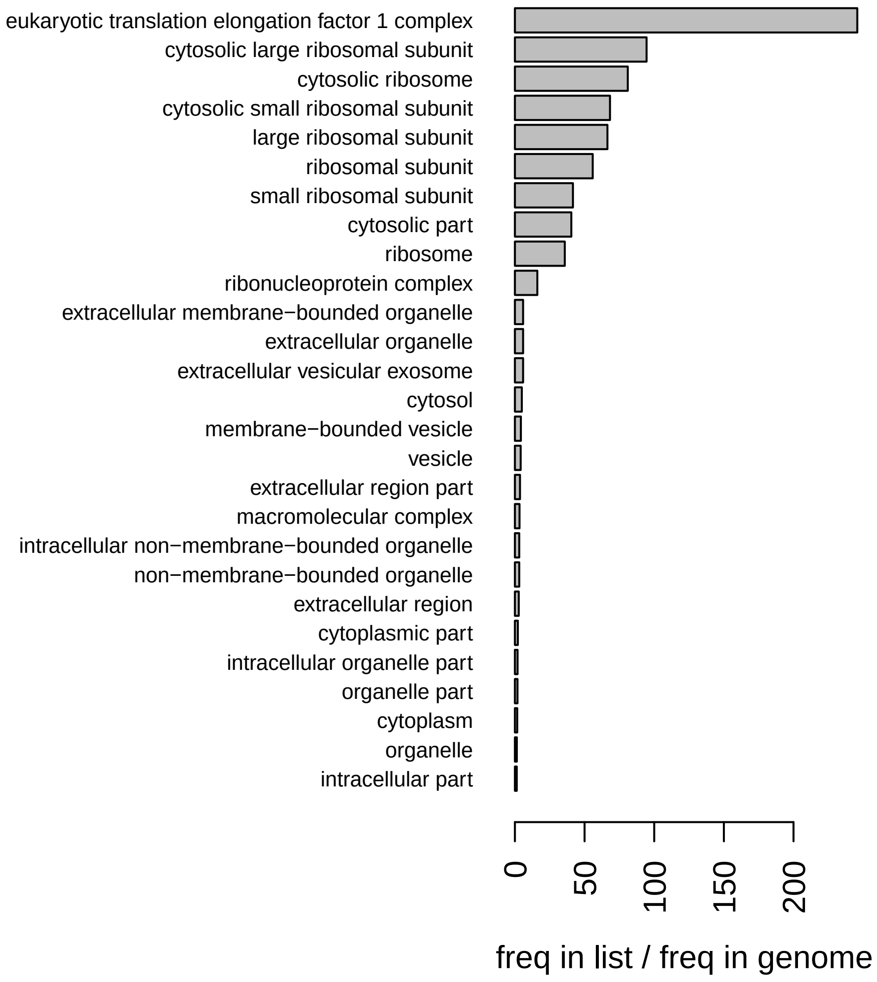

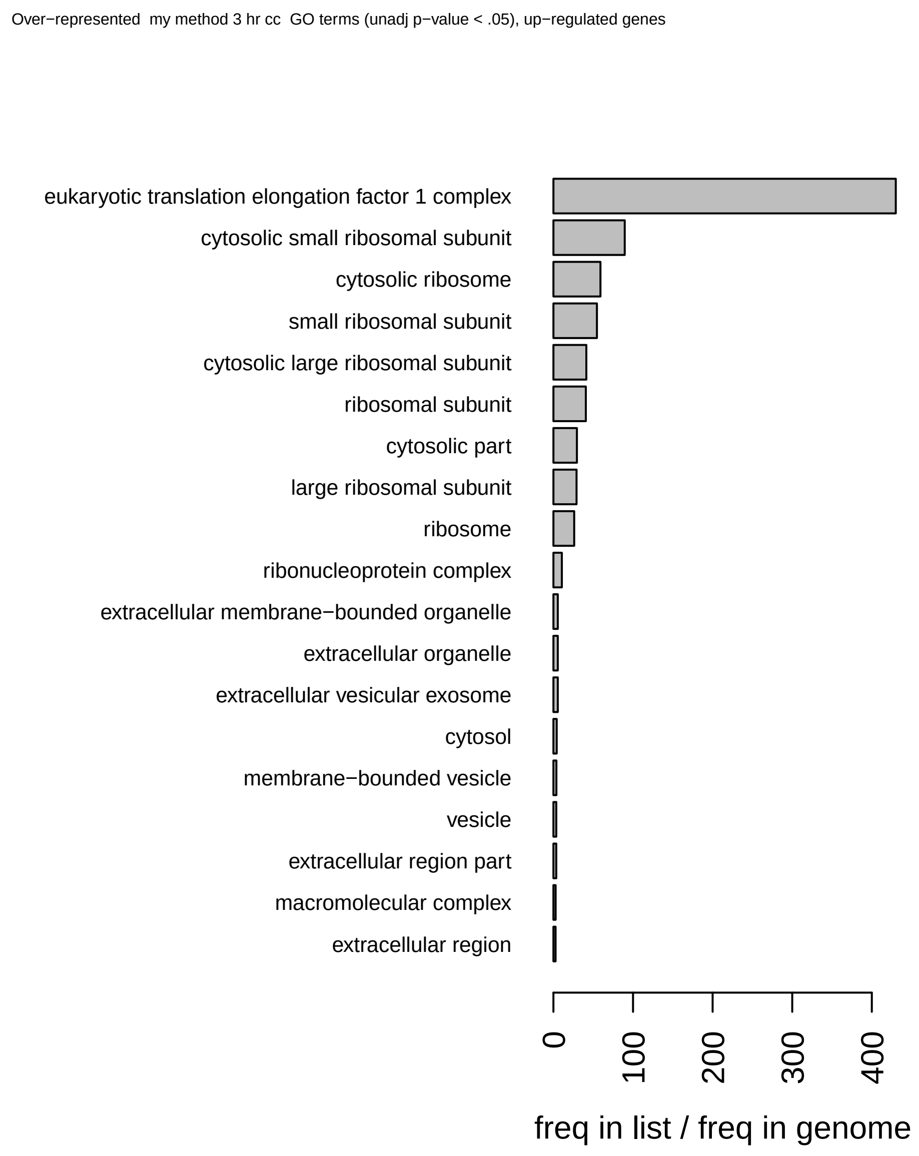
Over−represented my method 3 hr cc GO terms (unadj p−value < .05), up−regulated genes
eukaryotic translation elongation factor 1 complex
cytosolic small ribosomal subunit
cytosolic ribosome
small ribosomal subunit
cytosolic large ribosomal subunit
ribosomal subunit
cytosolic part
large ribosomal subunit
ribosome
ribonucleoprotein complex
extracellular membrane−bounded organelle
extracellular organelle
extracellular vesicular exosome
cytosol
membrane−bounded vesicle
vesicle
extracellular region part
macromolecular complex
extracellular region
0
100
200
300
400
freq in list / freq in genome

Over−represented my method 500 3 hr cc GO terms (unadj p−value < .05), up−regulated genes

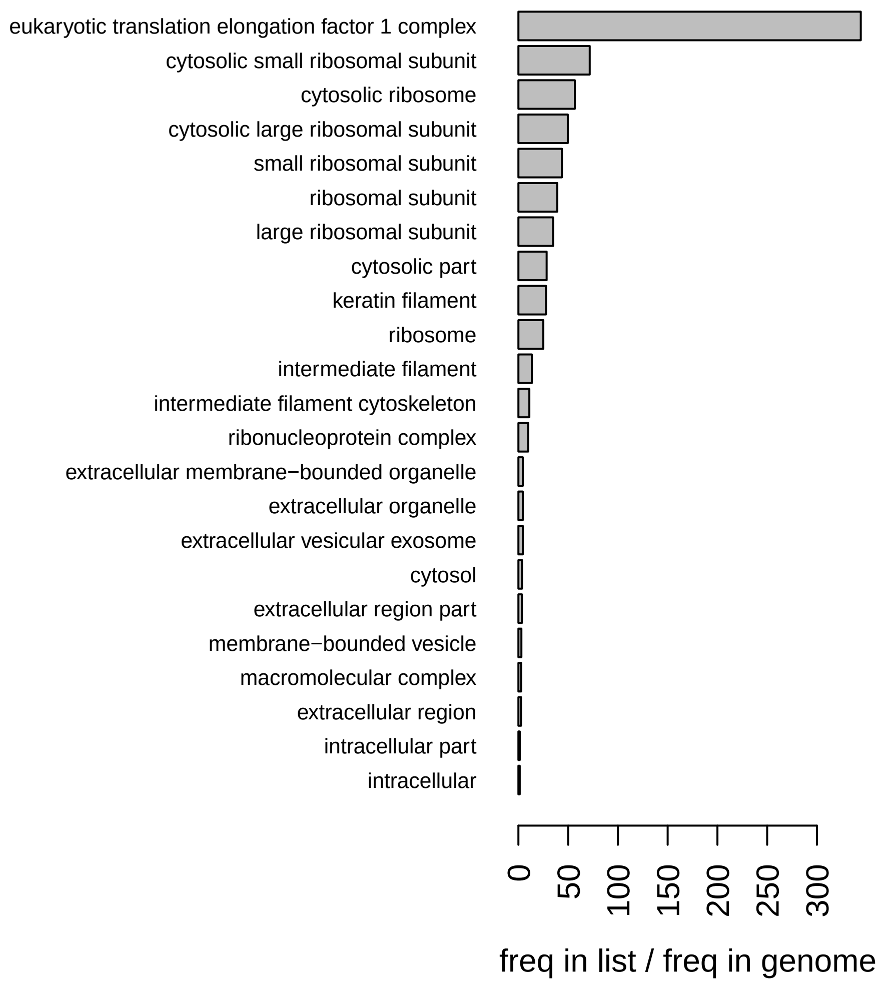

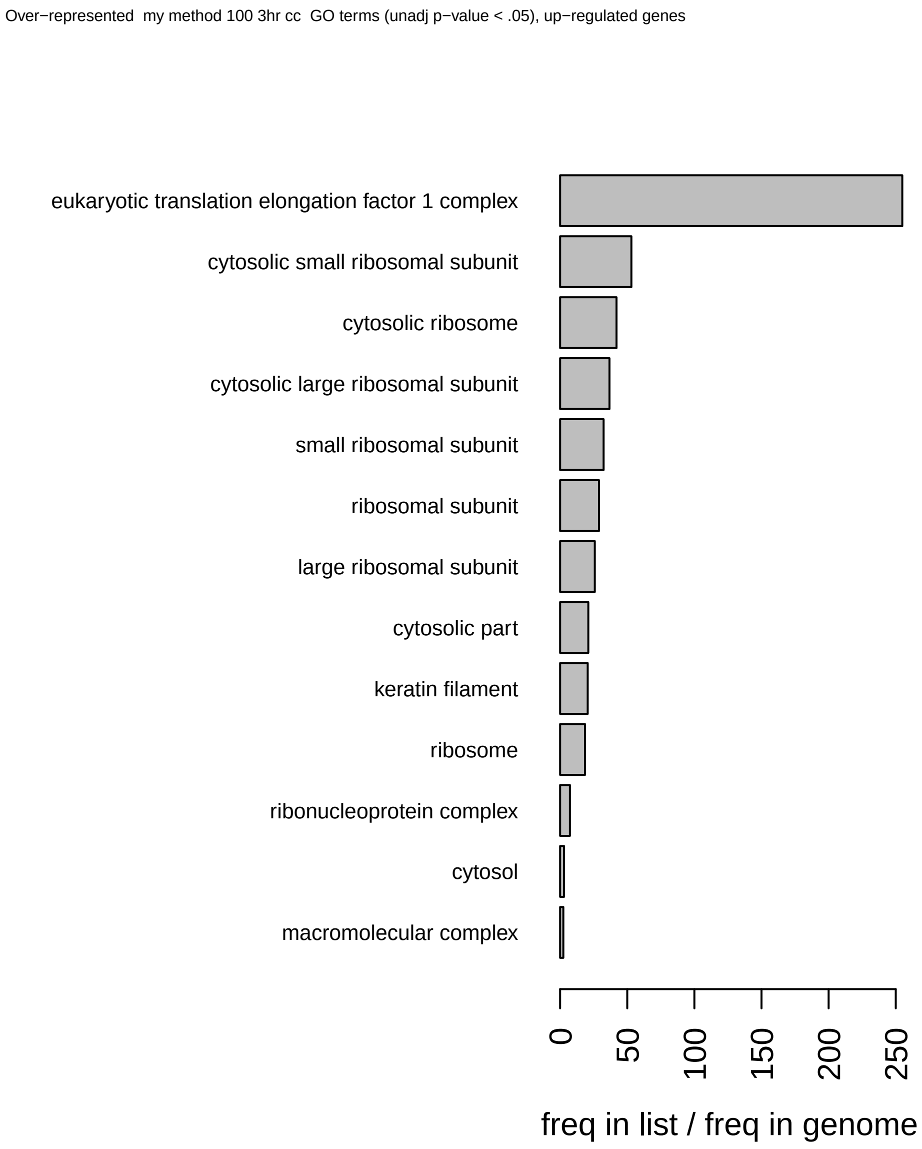
Over−represented my method 100 3hr cc GO terms (unadj p−value < .05), up−regulated genes
eukaryotic translation elongation factor 1 complex
cytosolic small ribosomal subunit
cytosolic ribosome
cytosolic large ribosomal subunit
small ribosomal subunit
ribosomal subunit
large ribosomal subunit
cytosolic part
keratin filament
ribosome
ribonucleoprotein complex
cytosol
macromolecular complex
0
50
100
150
200
250
freq in list / freq in genome

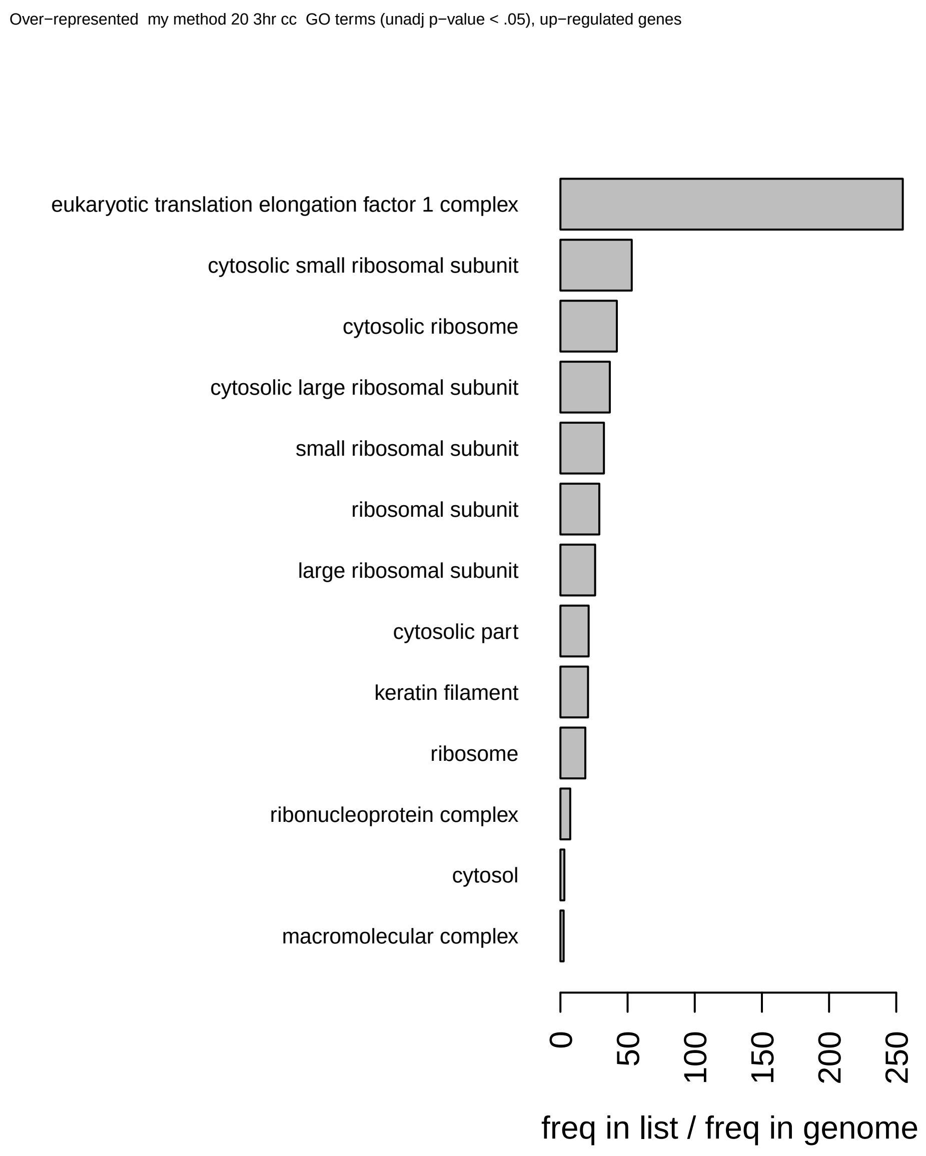
Over−represented my method 20 3hr cc GO terms (unadj p−value < .05), up−regulated genes
eukaryotic translation elongation factor 1 complex
cytosolic small ribosomal subunit
cytosolic ribosome
cytosolic large ribosomal subunit
small ribosomal subunit
ribosomal subunit
large ribosomal subunit
cytosolic part
keratin filament
ribosome
ribonucleoprotein complex
cytosol
macromolecular complex
0
50
100
150
200
250
freq in list / freq in genome

Over−represented my method 1000 24hr 2−fold cc GO terms (unadj p−value < .05), up−regulated genes

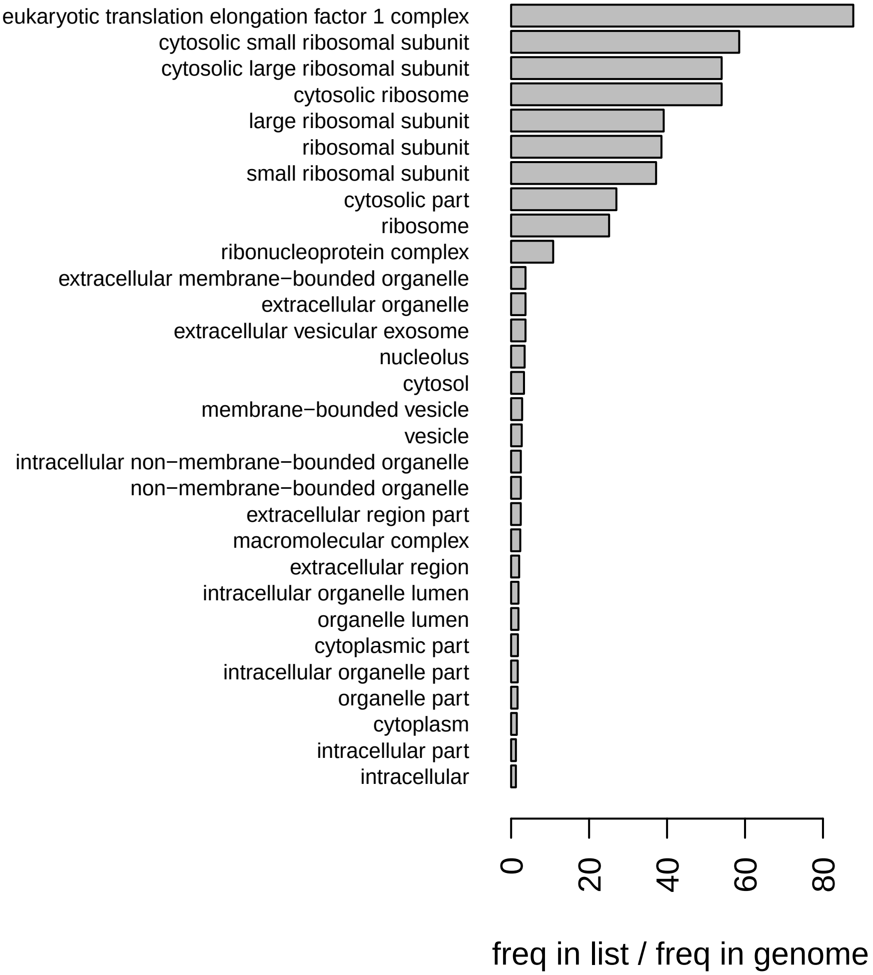

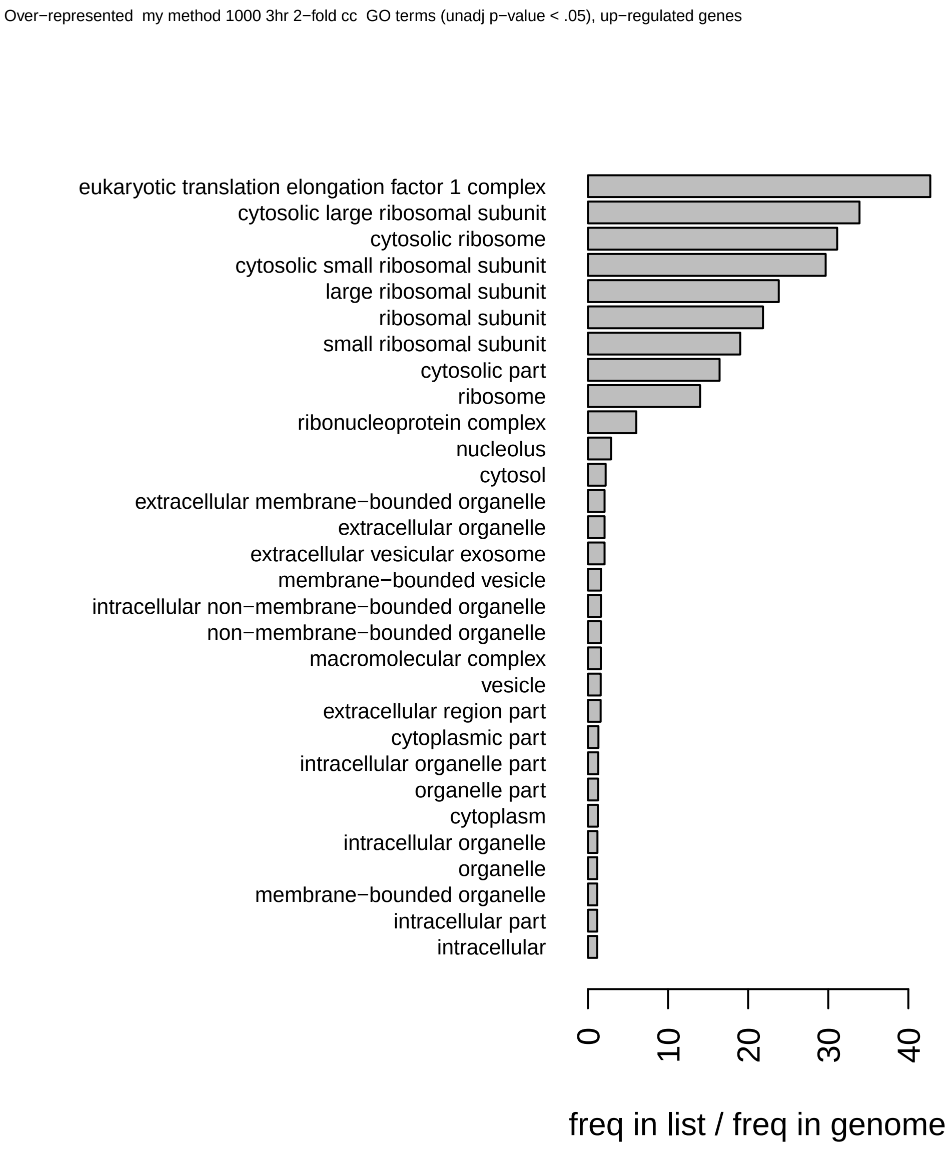
Over−represented my method 1000 3hr 2−fold cc GO terms (unadj p−value < .05), up−regulated genes
eukaryotic translation elongation factor 1 complex
cytosolic large ribosomal subunit
cytosolic ribosome
cytosolic small ribosomal subunit
large ribosomal subunit
ribosomal subunit
small ribosomal subunit
cytosolic part
ribosome
ribonucleoprotein complex
nucleolus
cytosol
extracellular membrane−bounded organelle
extracellular organelle
extracellular vesicular exosome
membrane−bounded vesicle
intracellular non−membrane−bounded organelle
non−membrane−bounded organelle
macromolecular complex
vesicle
extracellular region part
cytoplasmic part
intracellular organelle part
organelle part
cytoplasm
intracellular organelle
organelle
membrane−bounded organelle
intracellular part
intracellular
0
10
20
30
40
freq in list / freq in genome

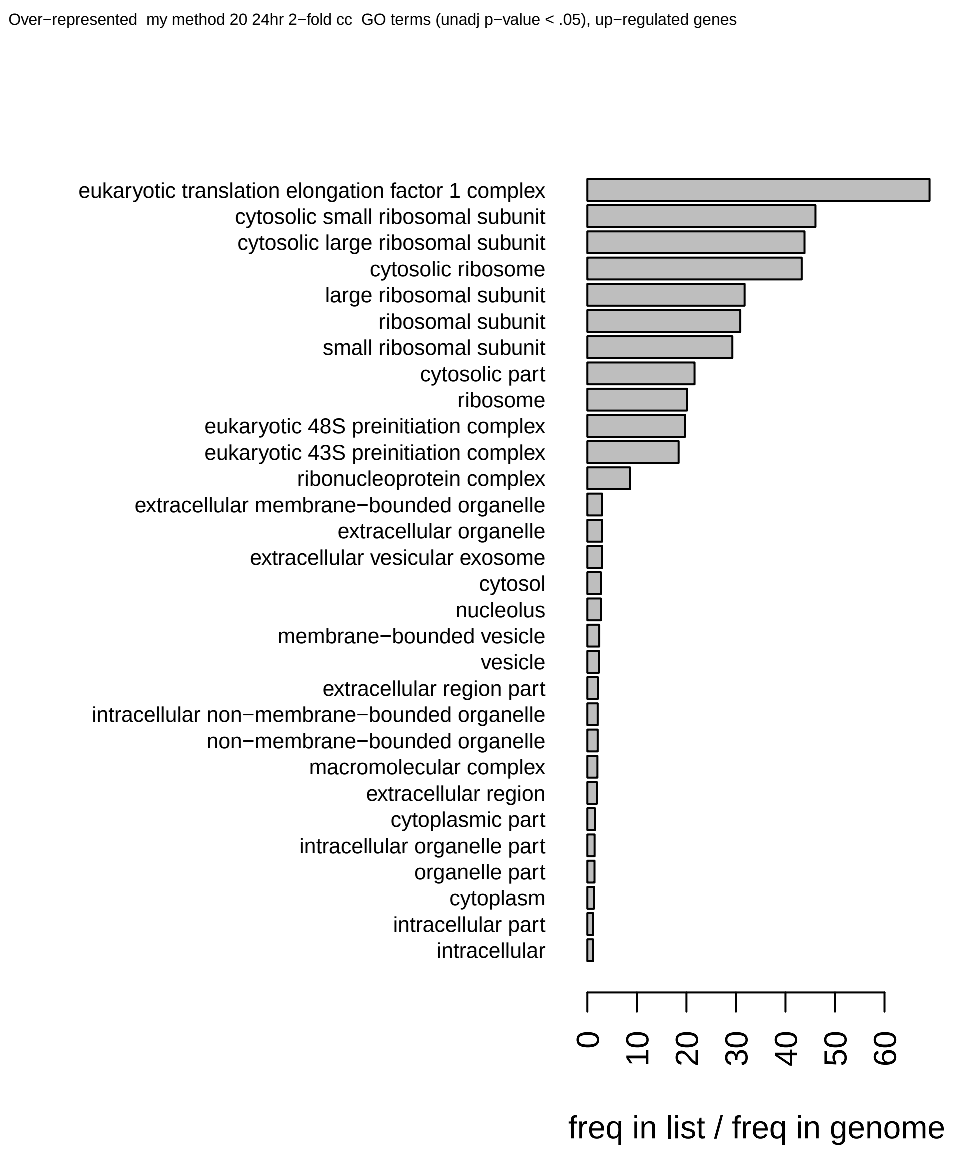
Over−represented my method 20 24hr 2−fold cc GO terms (unadj p−value < .05), up−regulated genes
eukaryotic translation elongation factor 1 complex
cytosolic small ribosomal subunit
cytosolic large ribosomal subunit
cytosolic ribosome
large ribosomal subunit
ribosomal subunit
small ribosomal subunit
cytosolic part
ribosome
eukaryotic 48S preinitiation complex
eukaryotic 43S preinitiation complex
ribonucleoprotein complex
extracellular membrane−bounded organelle
extracellular organelle
extracellular vesicular exosome
cytosol
nucleolus
membrane−bounded vesicle
vesicle
extracellular region part
intracellular non−membrane−bounded organelle
non−membrane−bounded organelle
macromolecular complex
extracellular region
cytoplasmic part
intracellular organelle part
organelle part
cytoplasm
intracellular part
intracellular
0
10
20
30
40
50
60
freq in list / freq in genome

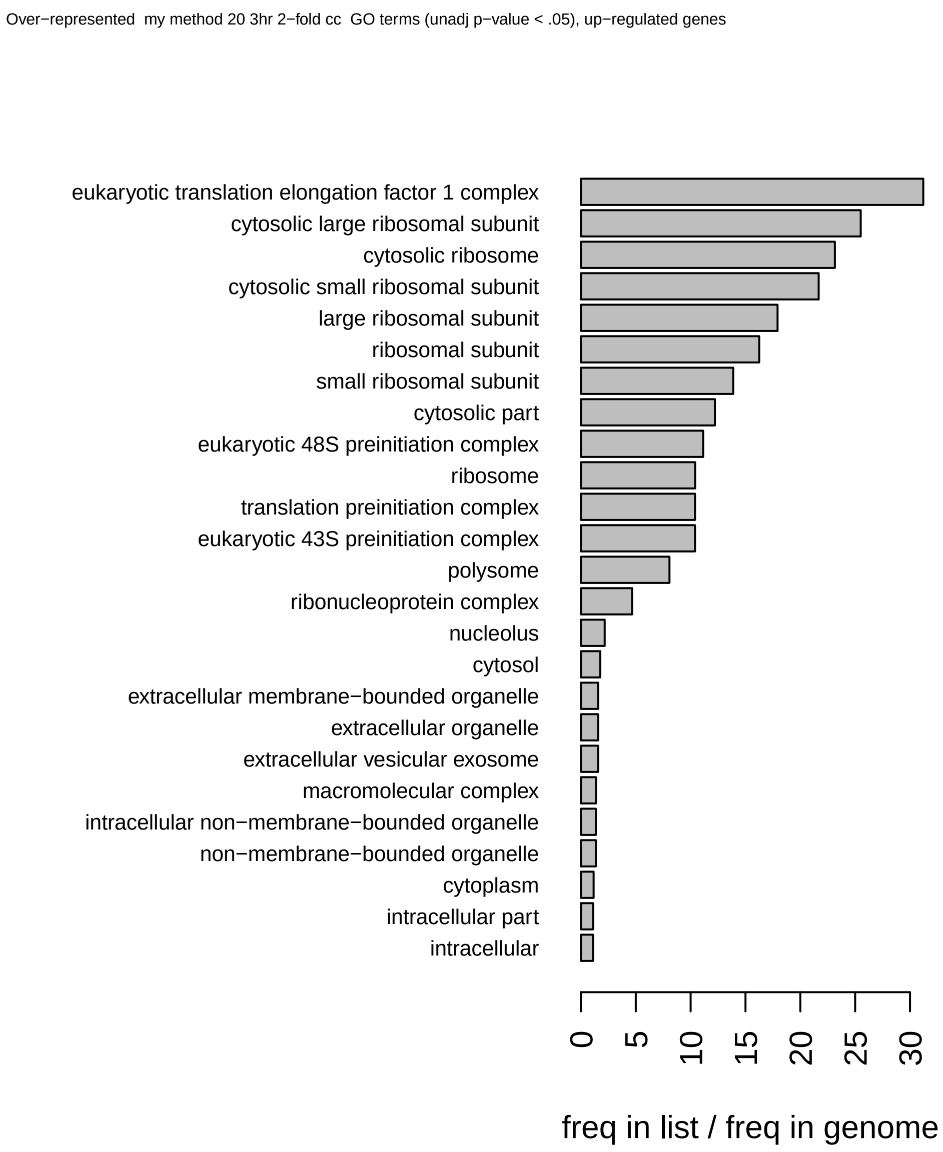
Over−represented my method 20 3hr 2−fold cc GO terms (unadj p−value < .05), up−regulated genes
eukaryotic translation elongation factor 1 complex
cytosolic large ribosomal subunit
cytosolic ribosome
cytosolic small ribosomal subunit
large ribosomal subunit
ribosomal subunit
small ribosomal subunit
cytosolic part
eukaryotic 48S preinitiation complex
ribosome
translation preinitiation complex
eukaryotic 43S preinitiation complex
polysome
ribonucleoprotein complex
nucleolus
cytosol
extracellular membrane−bounded organelle
extracellular organelle
extracellular vesicular exosome
macromolecular complex
intracellular non−membrane−bounded organelle
non−membrane−bounded organelle
cytoplasm
intracellular part
intracellular
0
5
10
15
20
25
30
freq in list / freq in genome
