# Supplementary material for: Improved transcription and translation with L-leucine stimulation of mTORC1 in Roberts syndrome
Source: BMC Genomics. 2016 Jan 5;17:25. doi: 10.1186/s12864-015-2354-y (PMC4700579; doi:10.1186/s12864-015-2354-y)
Supplement: Additional file 2: Table S1. — Summary table of Babel analysis. Table S2. GO term analysis of genes that show improved translational efficiency in RBS cells with L-leucine. Table S3. List for genes with fold change in translational efficiency greater than two and a total number of reads over 20 counts at 3 h. Table S4. List for genes with fold change in translational efficiency greater than two and a total number of reads over 20 counts at 24 h. Table S5. GO term analysis for genes with the new motif. Table S6. List of genes that have the new motif. Table S7. Summary table of data in boxplot of mTORC2-regulated gene show transcription and translation in Figure S5. Table S8. Summary table of data in heatmap for 868 mitochondrial genes—expression (Fig. 3) and translation. Table S9. GO term analysis for mitochondrial genes that show improve transcription with L-leucine. Table S10. GO term analysis for mitochondrial genes that improve translation with L-leucine. Table S11. Summary table of data in boxplot of snoRNAs expression in Fig. 4. Table S12. GO term analysis of differentially expressed genes in RBS cells. Table S13. List of genes in NADs and their expression, from Fig. 6. (ZIP 1.78 MB) [file 12864_2015_2354_MOESM2_ESM.zip › Table S12-Go_barplots_edg-differentially expressed genes.pdf]

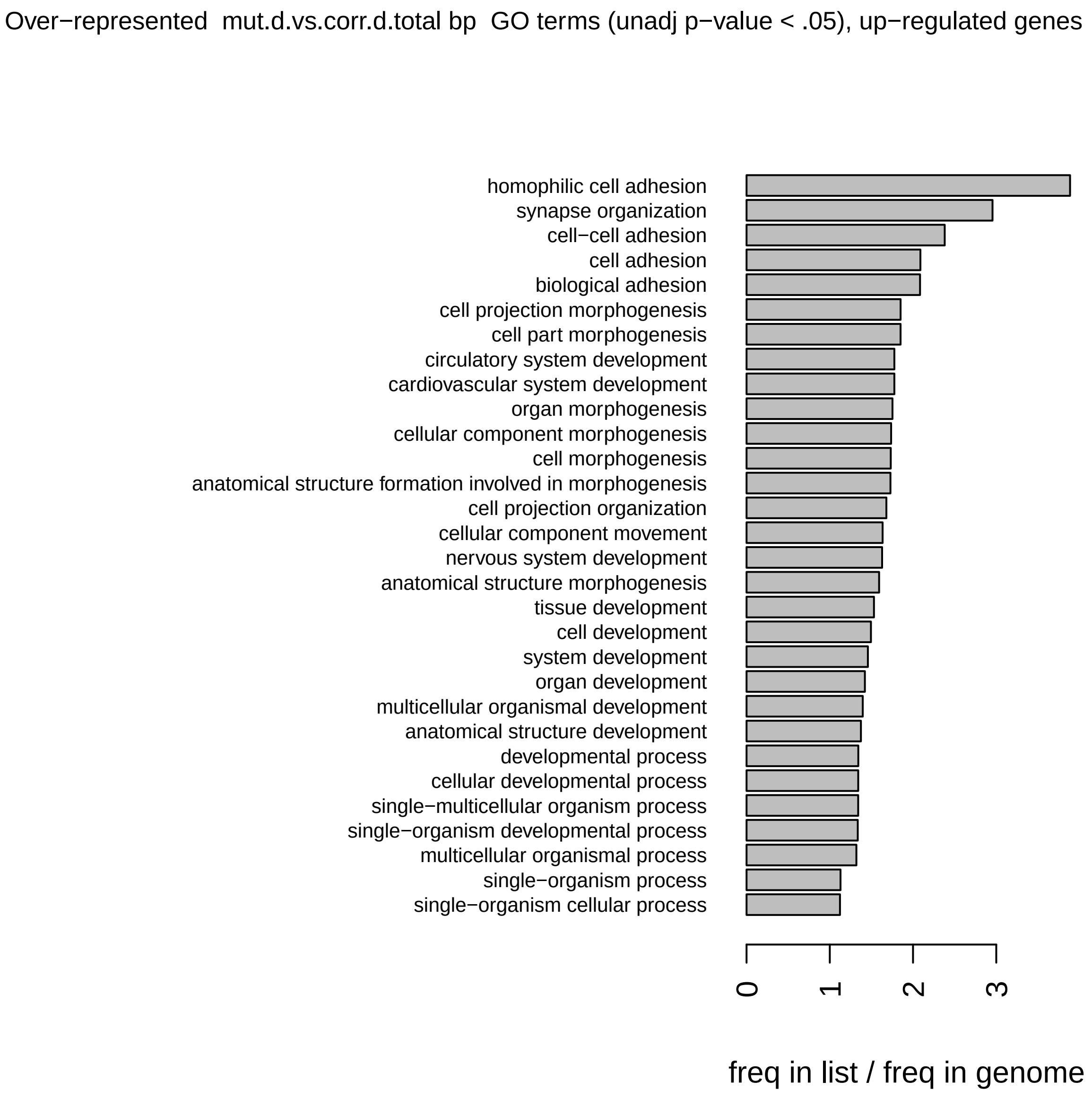

Over−represented mut.d.vs.corr.d.total bp GO terms (unadj p−value < .05), up−regulated genes
homophilic cell adhesion
synapse organization
cell−cell adhesion
cell adhesion
biological adhesion
cell projection morphogenesis
cell part morphogenesis
circulatory system development
cardiovascular system development
organ morphogenesis
cellular component morphogenesis
cell morphogenesis
anatomical structure formation involved in morphogenesis
cell projection organization
cellular component movement
nervous system development
anatomical structure morphogenesis
tissue development
cell development
system development
organ development
multicellular organismal development
anatomical structure development
developmental process
cellular developmental process
single−multicellular organism process
single−organism developmental process
multicellular organismal process
single−organism process
single−organism cellular process
0
1
2
3
freq in list / freq in genome

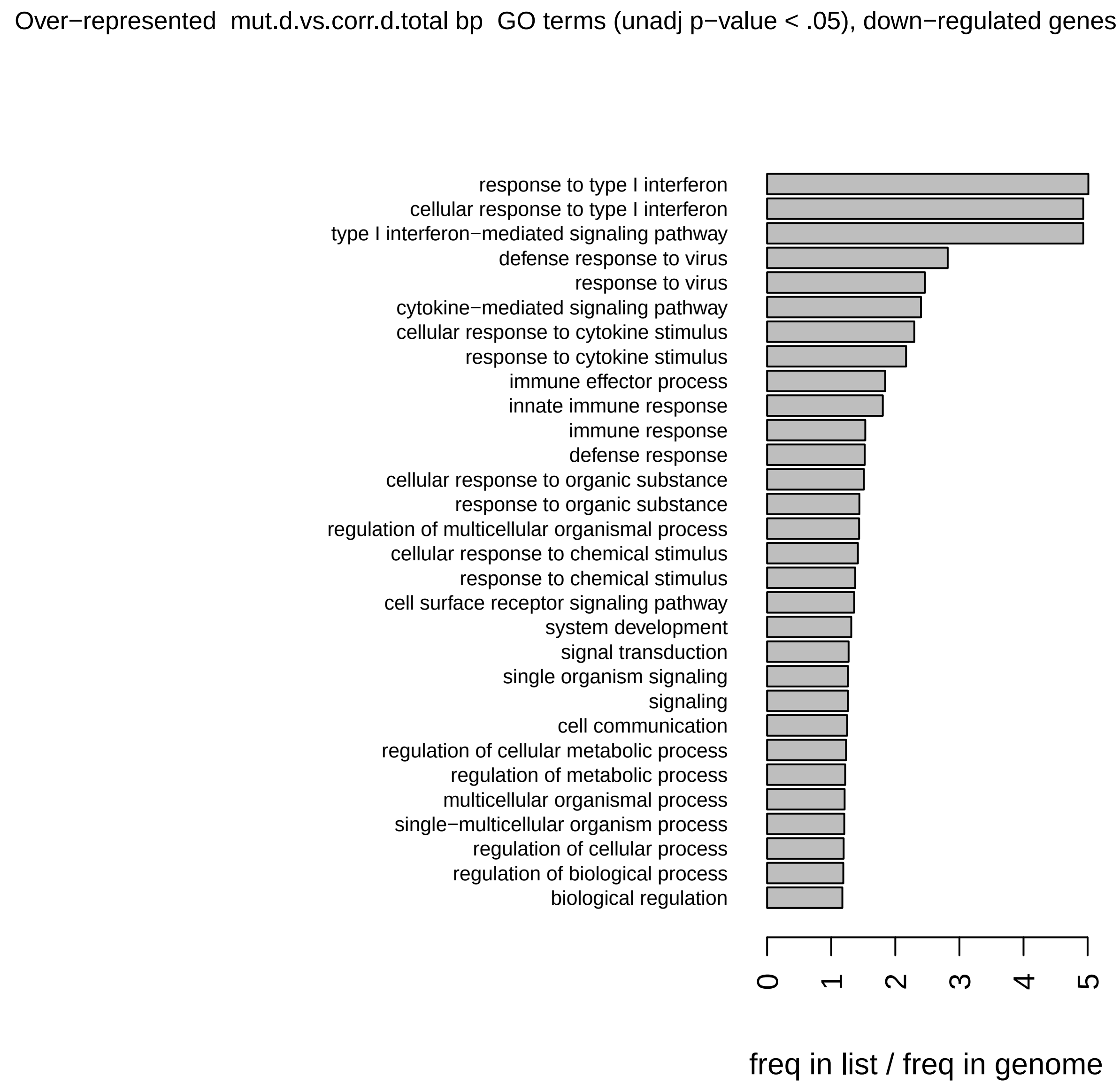

Over−represented mut.d.vs.corr.d.total bp GO terms (unadj p−value < .05), down−regulated genes
response to type I interferon
cellular response to type I interferon
type I interferon−mediated signaling pathway
defense response to virus
response to virus
cytokine−mediated signaling pathway
cellular response to cytokine stimulus
response to cytokine stimulus
immune effector process
innate immune response
immune response
defense response
cellular response to organic substance
response to organic substance
regulation of multicellular organismal process
cellular response to chemical stimulus
response to chemical stimulus
cell surface receptor signaling pathway
system development
signal transduction
single organism signaling
signaling
cell communication
regulation of cellular metabolic process
regulation of metabolic process
multicellular organismal process
single−multicellular organism process
regulation of cellular process
regulation of biological process
biological regulation
0
1
2
3
4
5
freq in list / freq in genome

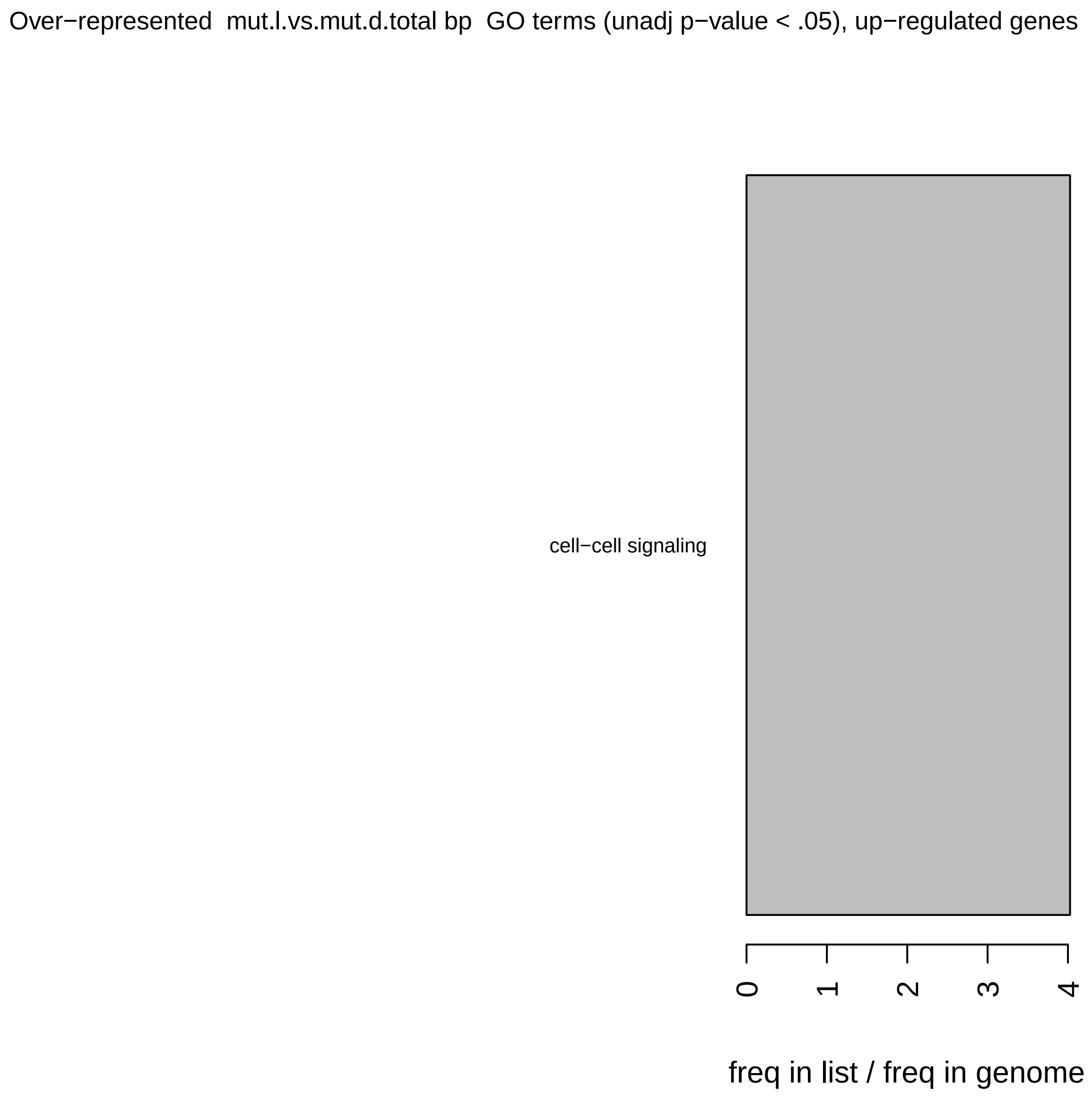
Over−represented mut.l.vs.mut.d.total bp GO terms (unadj p−value < .05), up−regulated genes
cell−cell signaling
0
1
2
3
4
freq in list / freq in genome

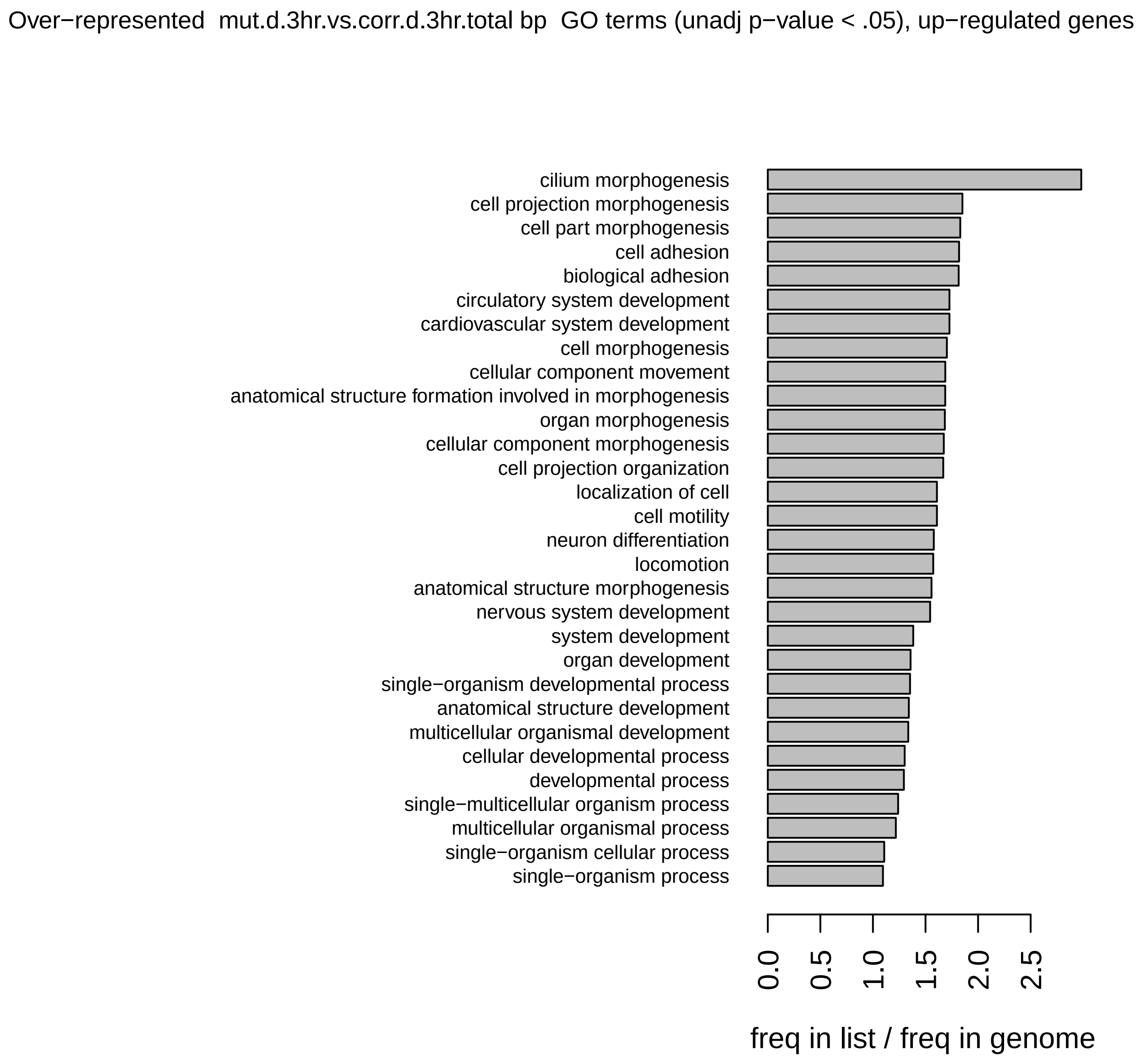
Over−represented mut.d.3hr.vs.corr.d.3hr.total bp GO terms (unadj p−value < .05), up−regulated genes
cilium morphogenesis
cell projection morphogenesis
cell part morphogenesis
cell adhesion
biological adhesion
circulatory system development
cardiovascular system development
cell morphogenesis
cellular component movement
anatomical structure formation involved in morphogenesis
organ morphogenesis
cellular component morphogenesis
cell projection organization
localization of cell
cell motility
neuron differentiation
locomotion
anatomical structure morphogenesis
nervous system development
system development
organ development
single−organism developmental process
anatomical structure development
multicellular organismal development
cellular developmental process
developmental process
single−multicellular organism process
multicellular organismal process
single−organism cellular process
single−organism process
0.0
0.5
1.0
1.5
2.0
2.5
freq in list / freq in genome

Over−represented mut.d.3hr.vs.corr.d.3hr.total bp GO terms (unadj p−value < .05), down−regulated genes

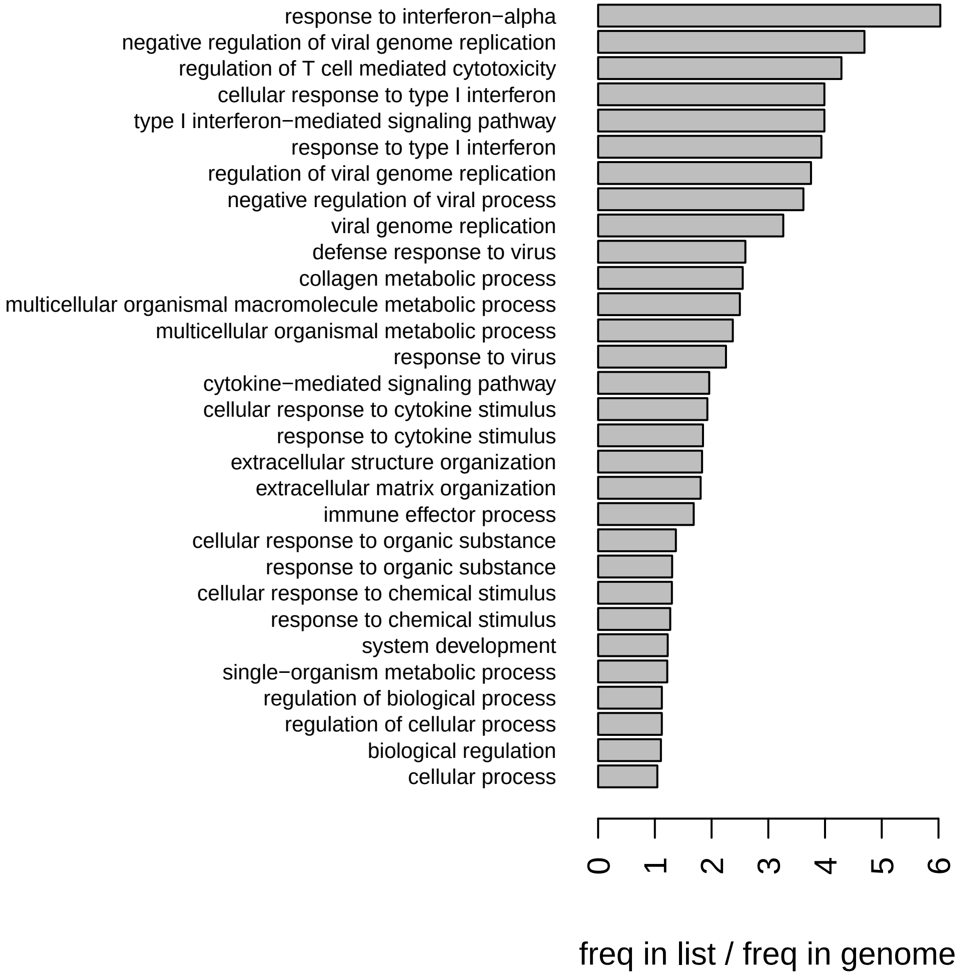

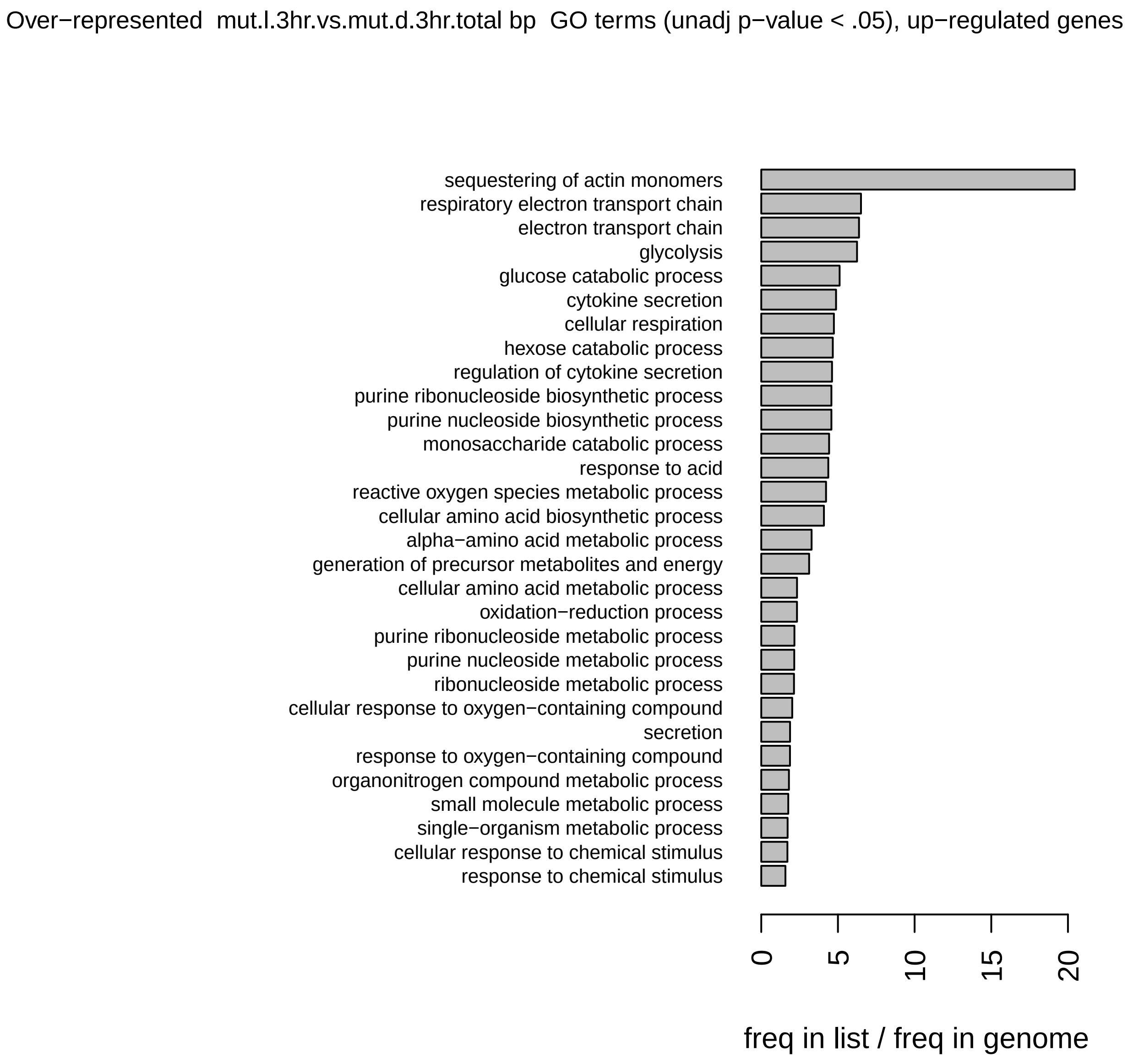

Over−represented mut.l.3hr.vs.mut.d.3hr.total bp GO terms (unadj p−value < .05), up−regulated genes
sequestering of actin monomers
respiratory electron transport chain
electron transport chain
glycolysis
glucose catabolic process
cytokine secretion
cellular respiration
hexose catabolic process
regulation of cytokine secretion
purine ribonucleoside biosynthetic process
purine nucleoside biosynthetic process
monosaccharide catabolic process
response to acid
reactive oxygen species metabolic process
cellular amino acid biosynthetic process
alpha−amino acid metabolic process
generation of precursor metabolites and energy
cellular amino acid metabolic process
oxidation−reduction process
purine ribonucleoside metabolic process
purine nucleoside metabolic process
ribonucleoside metabolic process
cellular response to oxygen−containing compound
secretion
response to oxygen−containing compound
organonitrogen compound metabolic process
small molecule metabolic process
single−organism metabolic process
cellular response to chemical stimulus
response to chemical stimulus
0
5
10
15
20
freq in list / freq in genome

Over−represented mut.l.3hr.vs.mut.d.3hr.total bp GO terms (unadj p−value < .05), down−regulated genes

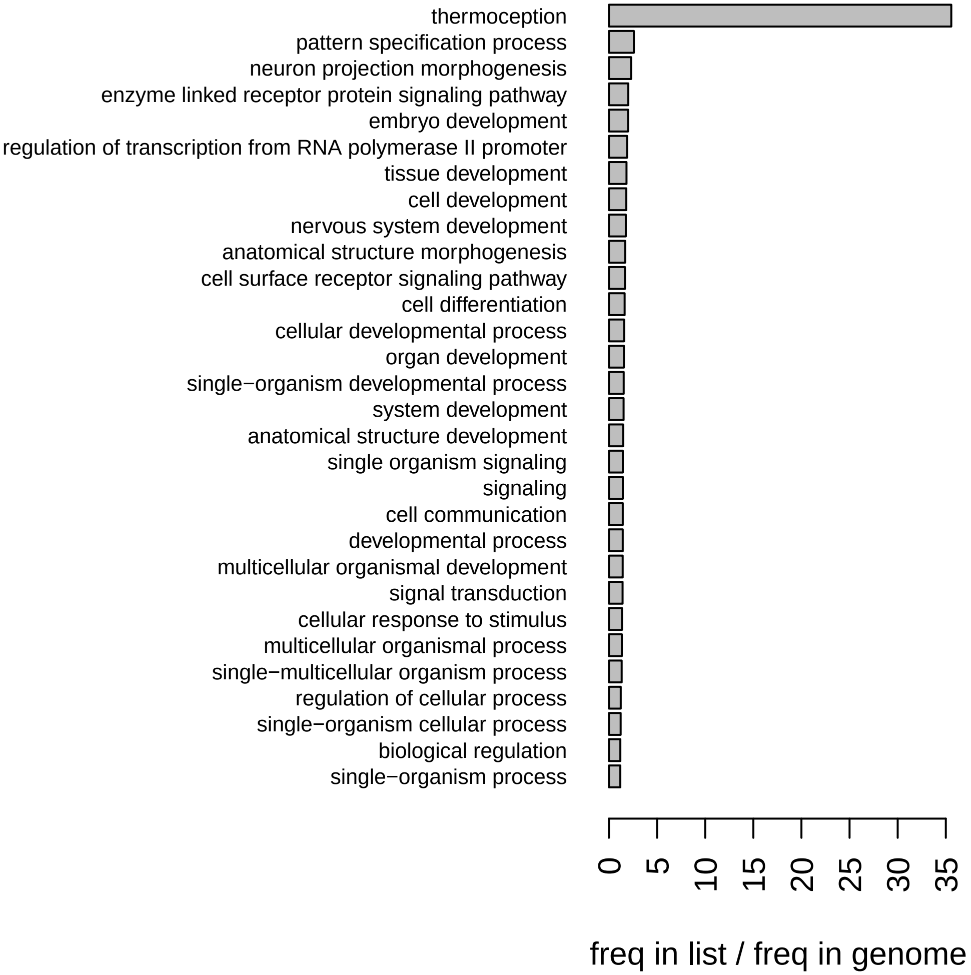

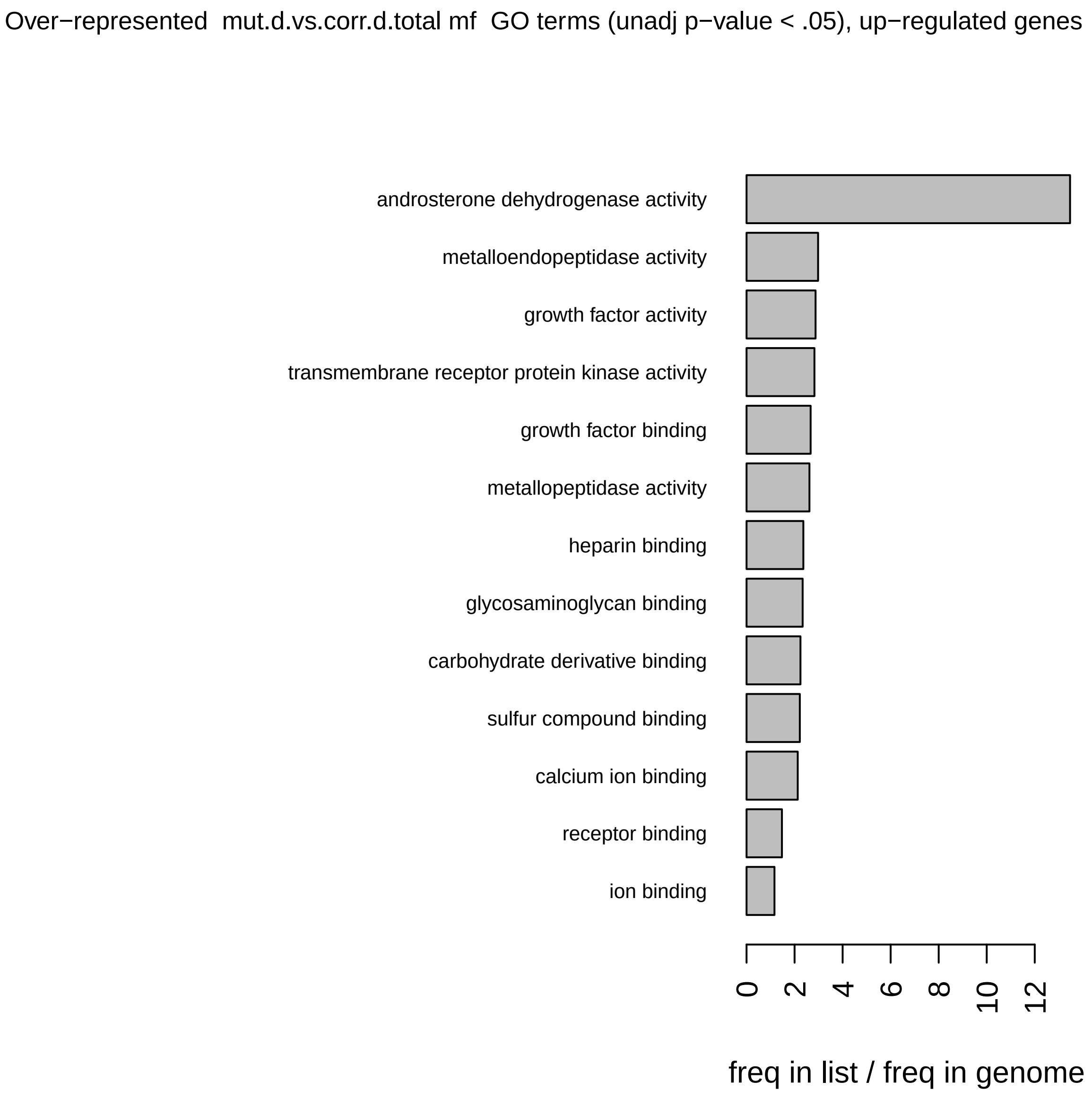
Over−represented mut.d.vs.corr.d.total mf GO terms (unadj p−value < .05), up−regulated genes
androsterone dehydrogenase activity
metalloendopeptidase activity
growth factor activity
transmembrane receptor protein kinase activity
growth factor binding
metallopeptidase activity
heparin binding
glycosaminoglycan binding
carbohydrate derivative binding
sulfur compound binding
calcium ion binding
receptor binding
ion binding
0
2
4
6
8
10
12
freq in list / freq in genome

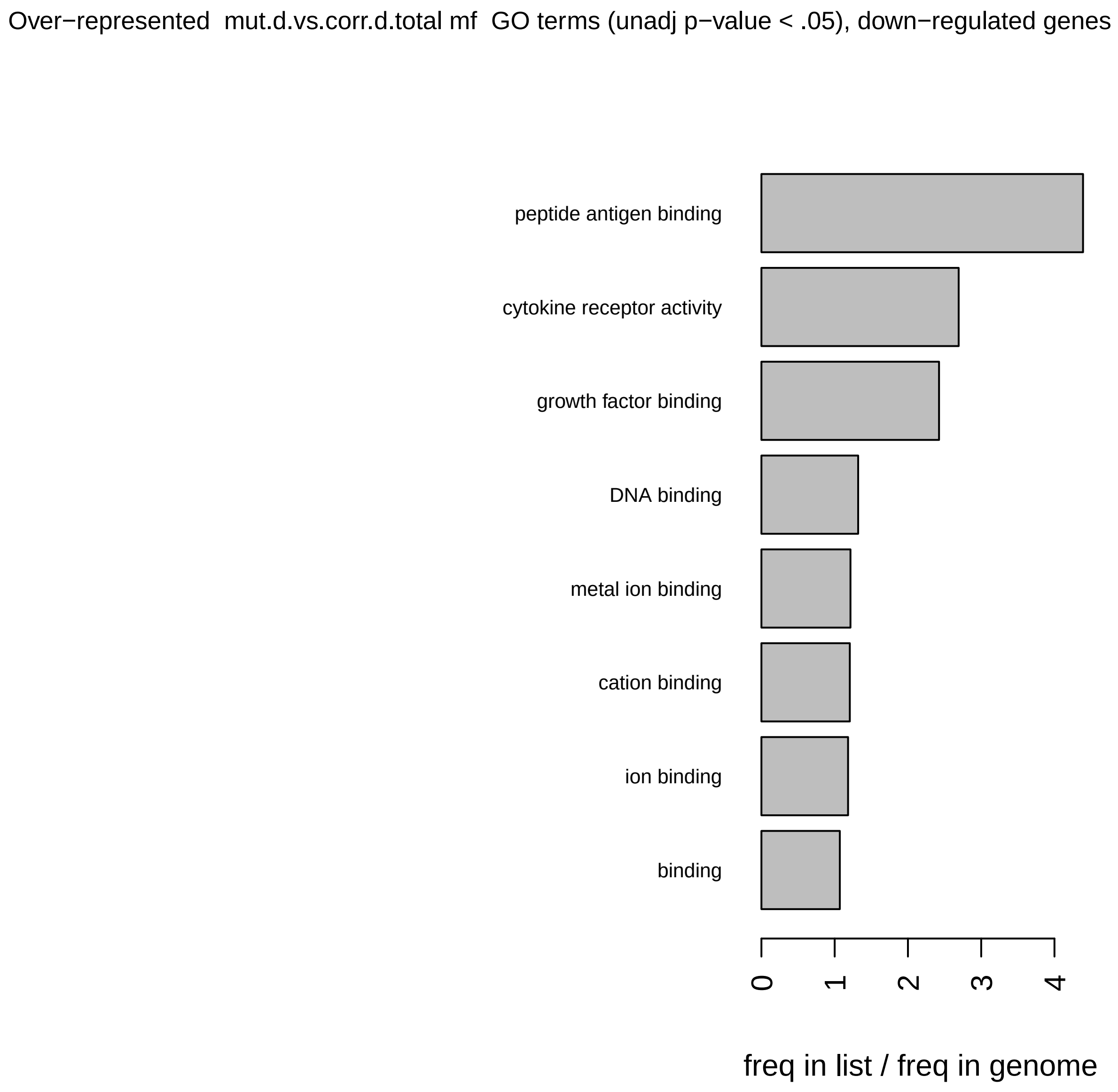
Over−represented mut.d.vs.corr.d.total mf GO terms (unadj p−value < .05), down−regulated genes
peptide antigen binding
cytokine receptor activity
growth factor binding
DNA binding
metal ion binding
cation binding
ion binding
binding
0
1
2
3
4
freq in list / freq in genome

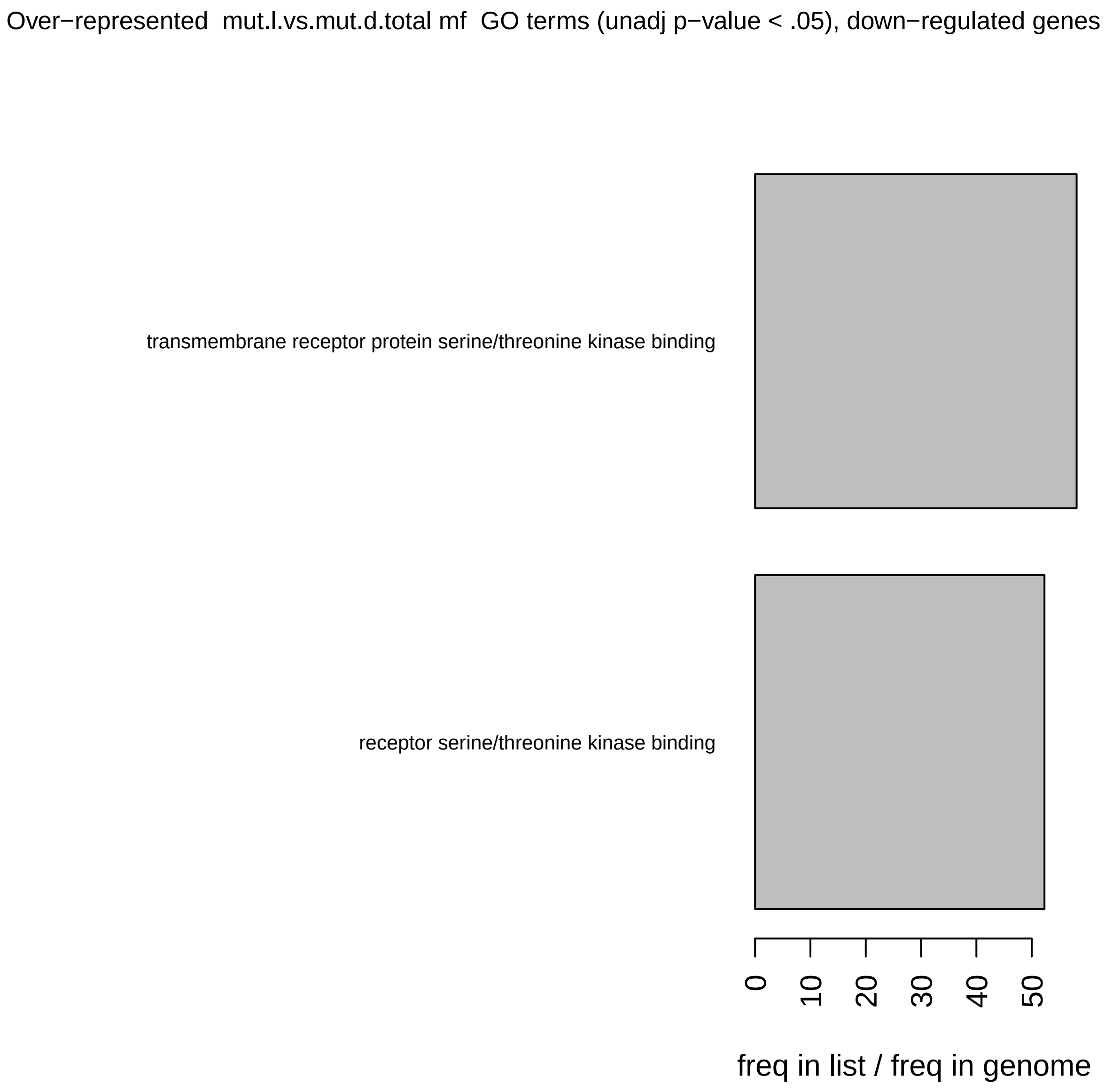

Over−represented mut.l.vs.mut.d.total mf GO terms (unadj p−value < .05), down−regulated genes
transmembrane receptor protein serine/threonine kinase binding
receptor serine/threonine kinase binding
0
10
20
30
40
50
freq in list / freq in genome

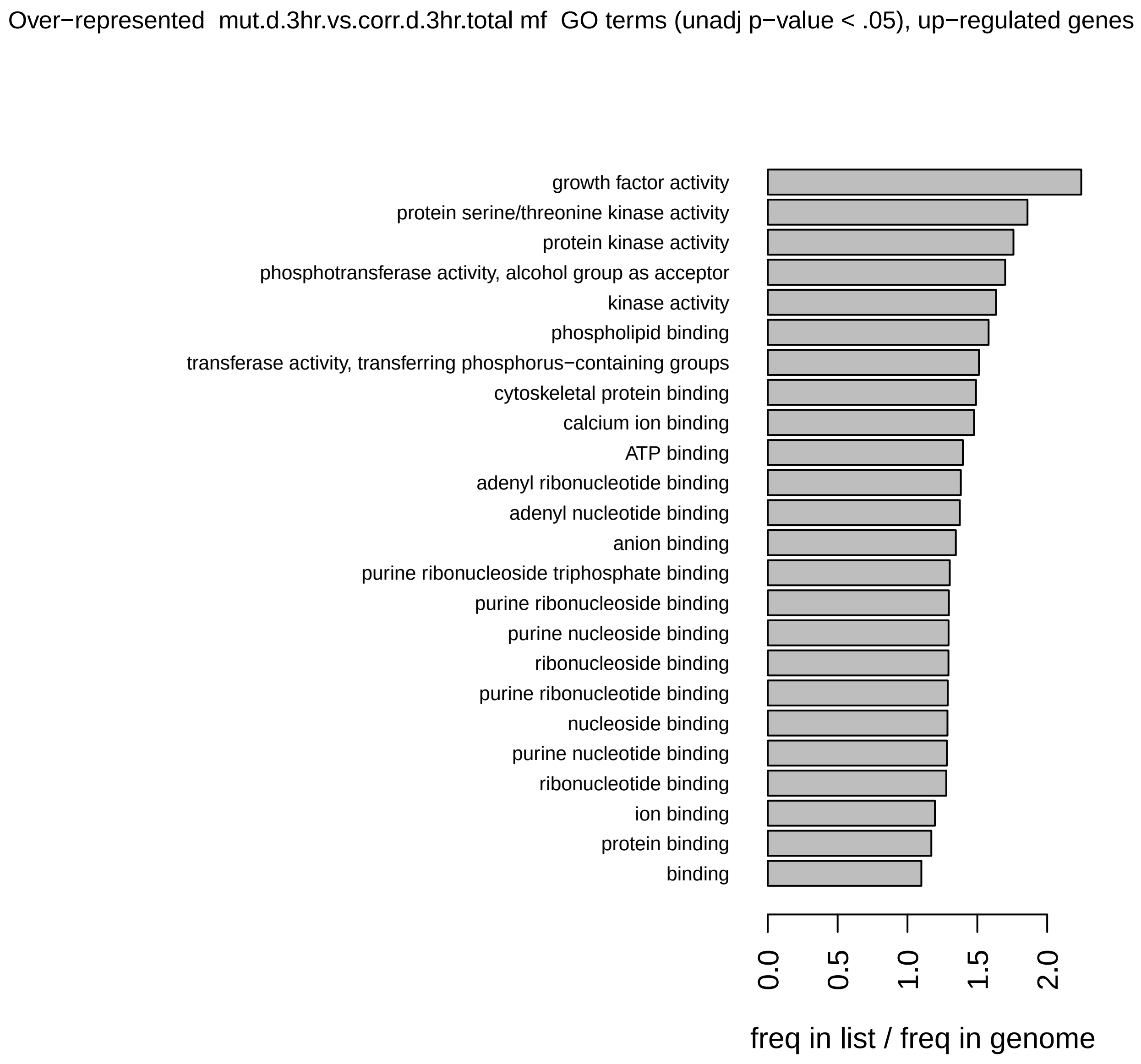
Over−represented mut.d.3hr.vs.corr.d.3hr.total mf GO terms (unadj p−value < .05), up−regulated genes
growth factor activity
protein serine/threonine kinase activity
protein kinase activity
phosphotransferase activity, alcohol group as acceptor
kinase activity
phospholipid binding
transferase activity, transferring phosphorus−containing groups
cytoskeletal protein binding
calcium ion binding
ATP binding
adenyl ribonucleotide binding
adenyl nucleotide binding
anion binding
purine ribonucleoside triphosphate binding
purine ribonucleoside binding
purine nucleoside binding
ribonucleoside binding
purine ribonucleotide binding
nucleoside binding
purine nucleotide binding
ribonucleotide binding
ion binding
protein binding
binding
0.0
0.5
1.0
1.5
2.0
freq in list / freq in genome

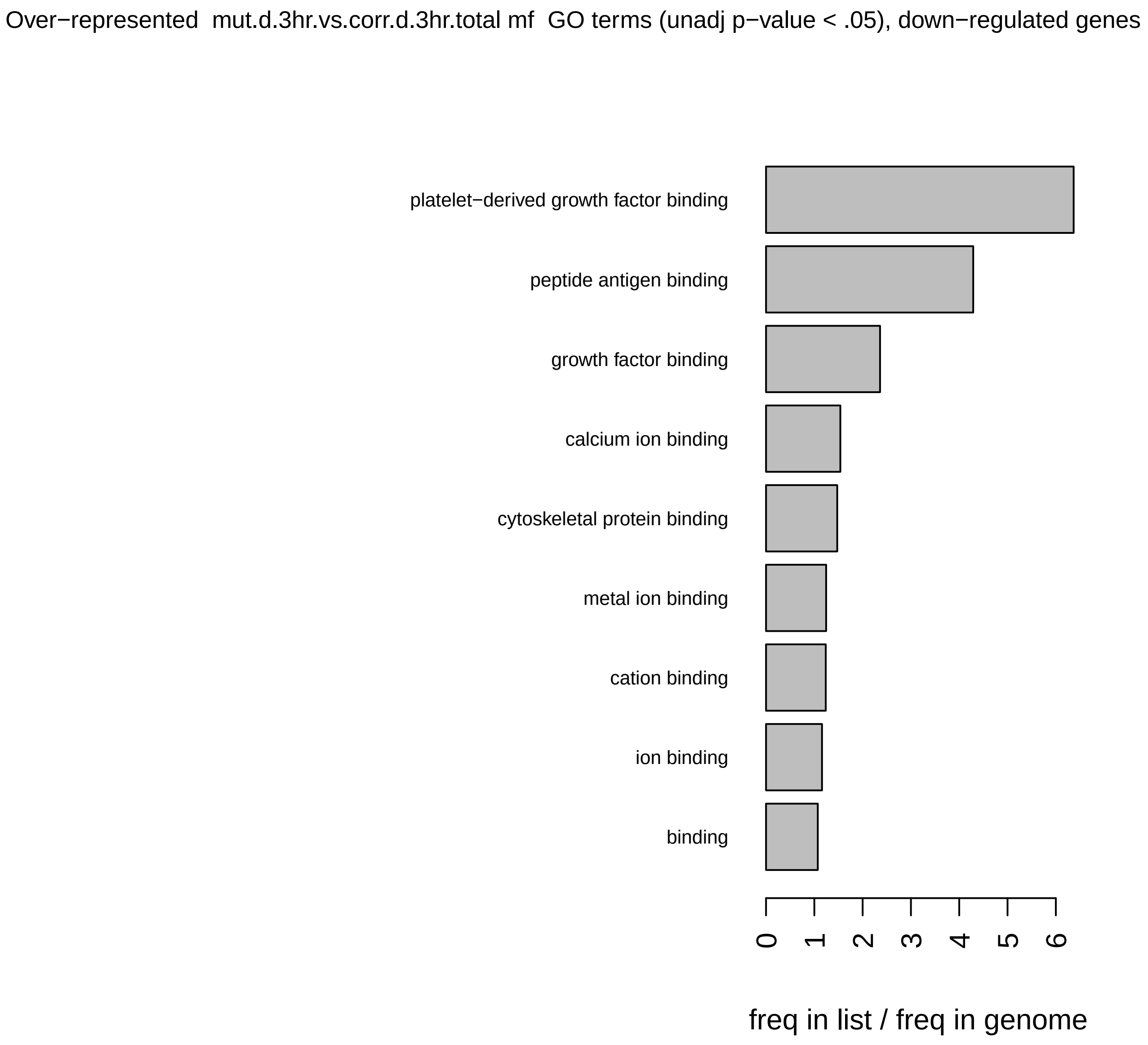

Over−represented mut.d.3hr.vs.corr.d.3hr.total mf GO terms (unadj p−value < .05), down−regulated genes
platelet−derived growth factor binding
peptide antigen binding
growth factor binding
calcium ion binding
cytoskeletal protein binding
metal ion binding
cation binding
ion binding
binding
0
1
2
3
4
5
6
freq in list / freq in genome

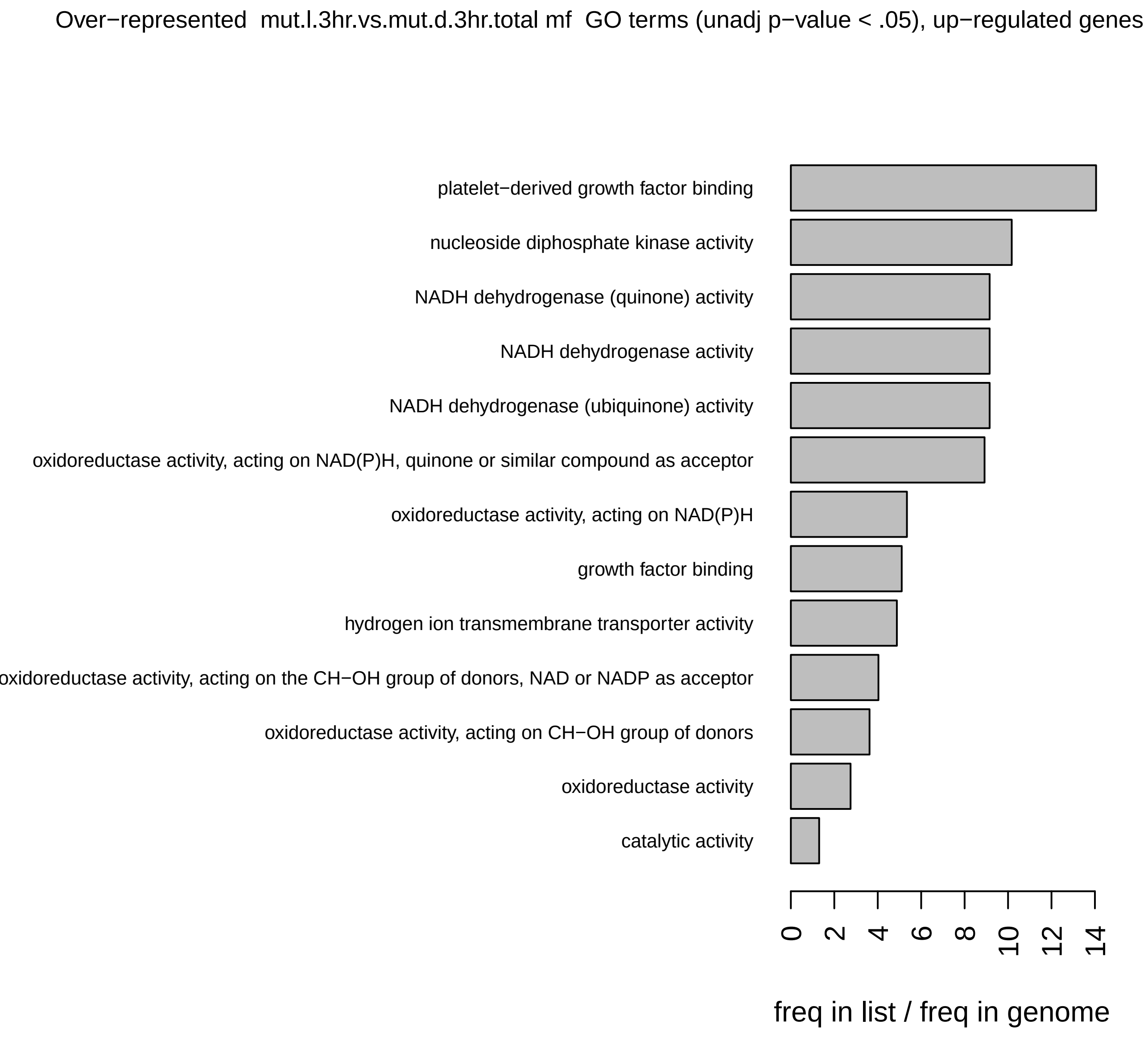

Over−represented mut.l.3hr.vs.mut.d.3hr.total mf GO terms (unadj p−value < .05), up−regulated genes
platelet−derived growth factor binding
nucleoside diphosphate kinase activity
NADH dehydrogenase (quinone) activity
NADH dehydrogenase activity
NADH dehydrogenase (ubiquinone) activity
oxidoreductase activity, acting on NAD(P)H, quinone or similar compound as acceptor
oxidoreductase activity, acting on NAD(P)H
growth factor binding
hydrogen ion transmembrane transporter activity
oxidoreductase activity, acting on the CH−OH group of donors, NAD or NADP as acceptor
oxidoreductase activity, acting on CH−OH group of donors
oxidoreductase activity
catalytic activity
0
2
4
6
8
10
12
14
freq in list / freq in genome

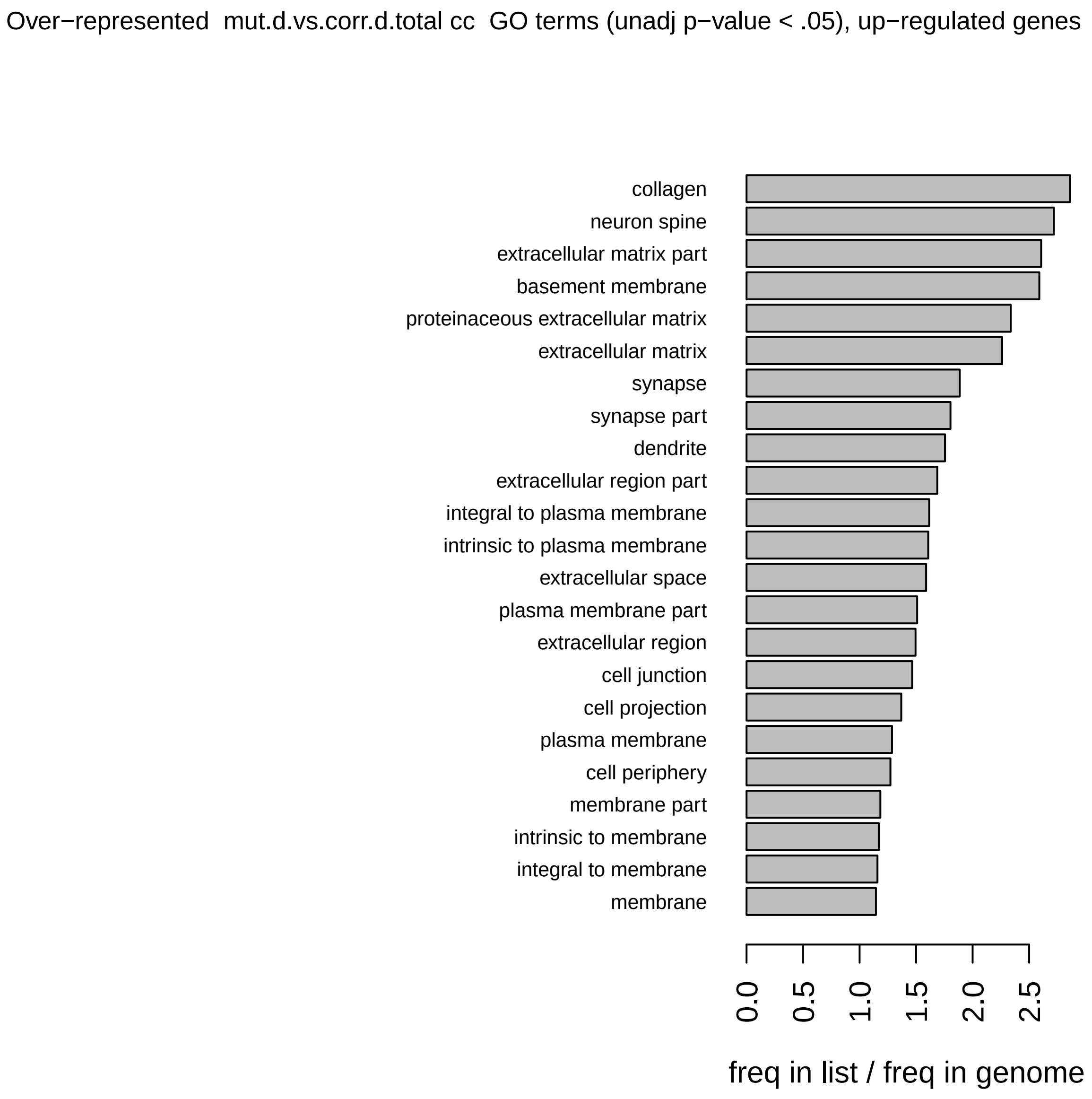

Over−represented mut.d.vs.corr.d.total cc GO terms (unadj p−value < .05), up−regulated genes
collagen
neuron spine
extracellular matrix part
basement membrane
proteinaceous extracellular matrix
extracellular matrix
synapse
synapse part
dendrite
extracellular region part
integral to plasma membrane
intrinsic to plasma membrane
extracellular space
plasma membrane part
extracellular region
cell junction
cell projection
plasma membrane
cell periphery
membrane part
intrinsic to membrane
integral to membrane
membrane
0.0
0.5
1.0
1.5
2.0
2.5
freq in list / freq in genome

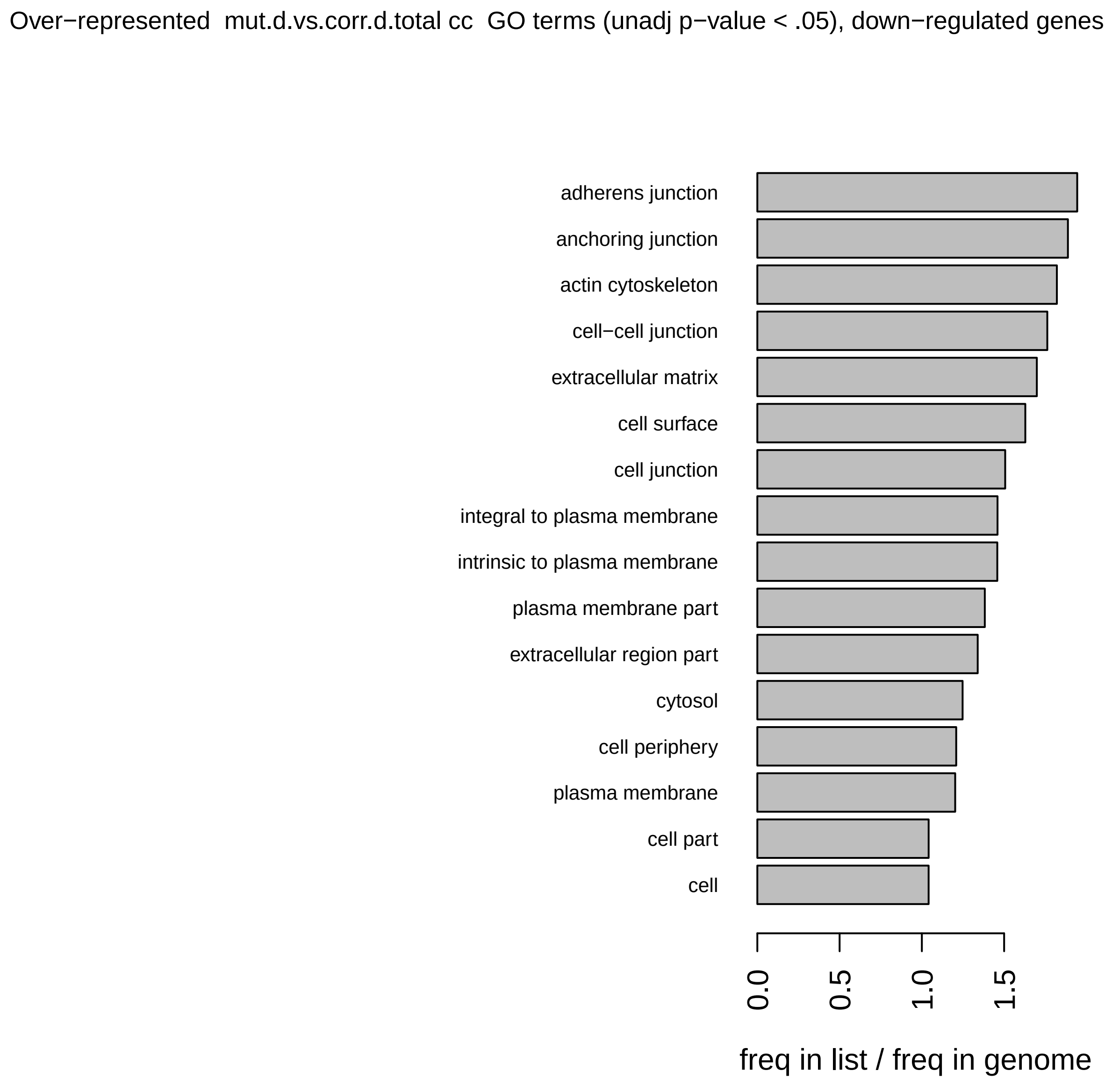
Over−represented mut.d.vs.corr.d.total cc GO terms (unadj p−value < .05), down−regulated genes
adherens junction
anchoring junction
actin cytoskeleton
cell−cell junction
extracellular matrix
cell surface
cell junction
integral to plasma membrane
intrinsic to plasma membrane
plasma membrane part
extracellular region part
cytosol
cell periphery
plasma membrane
cell part
cell
0.0
0.5
1.0
1.5
freq in list / freq in genome

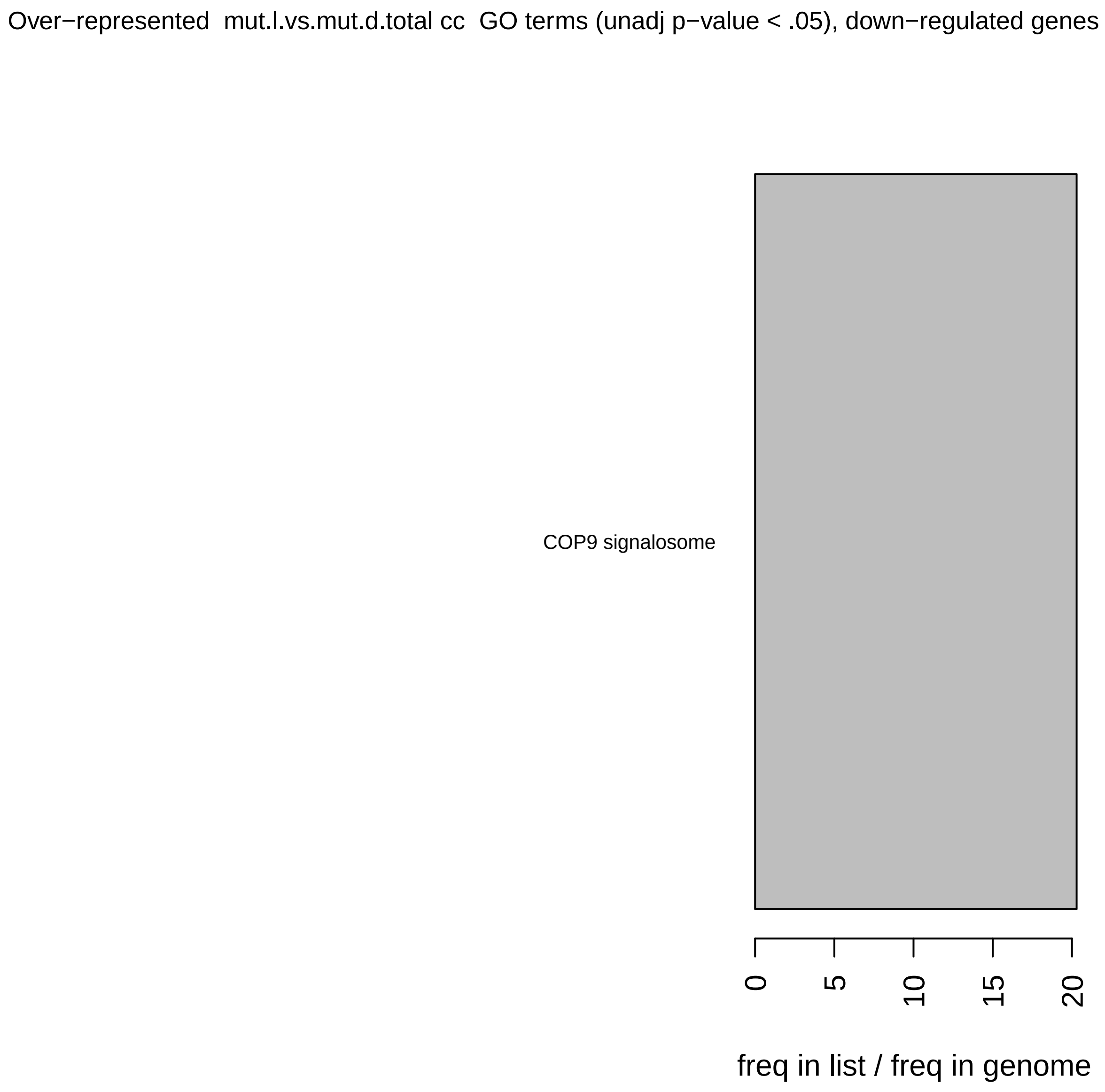
Over−represented mut.l.vs.mut.d.total cc GO terms (unadj p−value < .05), down−regulated genes
COP9 signalosome
0
5
10
15
20
freq in list / freq in genome

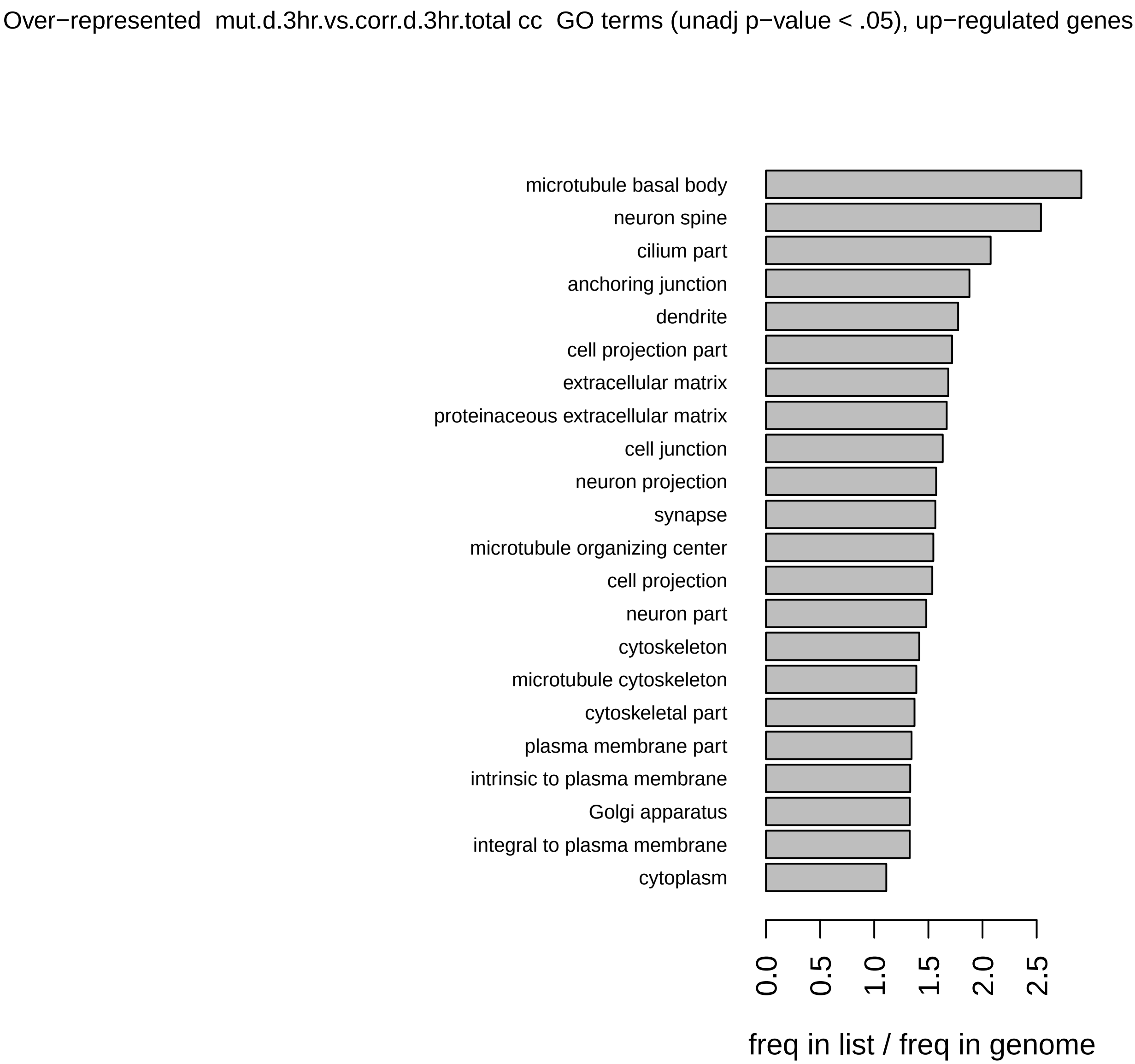
Over−represented mut.d.3hr.vs.corr.d.3hr.total cc GO terms (unadj p−value < .05), up−regulated genes
microtubule basal body
neuron spine
cilium part
anchoring junction
dendrite
cell projection part
extracellular matrix
proteinaceous extracellular matrix
cell junction
neuron projection
synapse
microtubule organizing center
cell projection
neuron part
cytoskeleton
microtubule cytoskeleton
cytoskeletal part
plasma membrane part
intrinsic to plasma membrane
Golgi apparatus
integral to plasma membrane
cytoplasm
0.0
0.5
1.0
1.5
2.0
2.5
freq in list / freq in genome

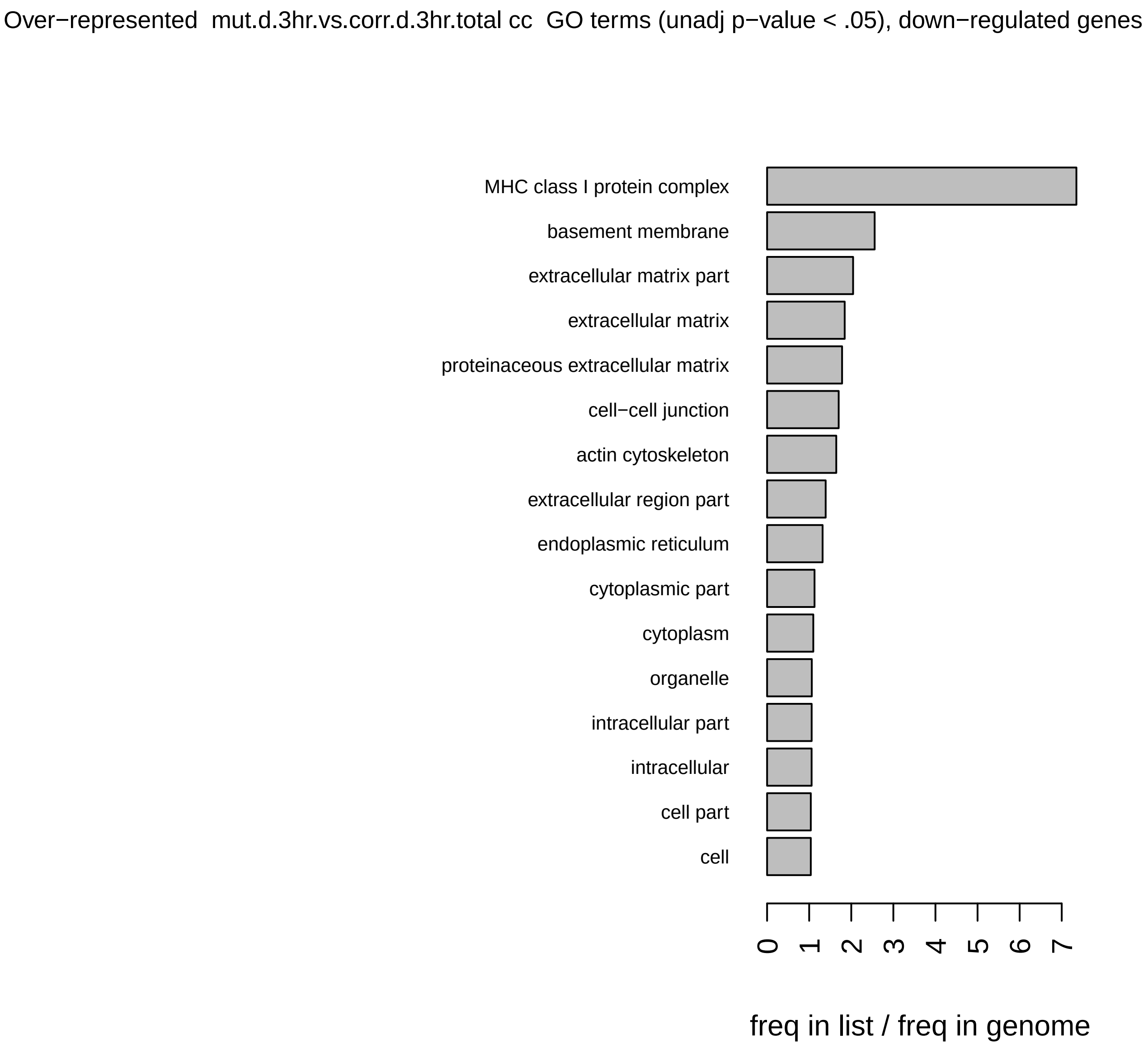
Over−represented mut.d.3hr.vs.corr.d.3hr.total cc GO terms (unadj p−value < .05), down−regulated genes
MHC class I protein complex
basement membrane
extracellular matrix part
extracellular matrix
proteinaceous extracellular matrix
cell−cell junction
actin cytoskeleton
extracellular region part
endoplasmic reticulum
cytoplasmic part
cytoplasm
organelle
intracellular part
intracellular
cell part
cell
0
1
2
3
4
5
6
7
freq in list / freq in genome

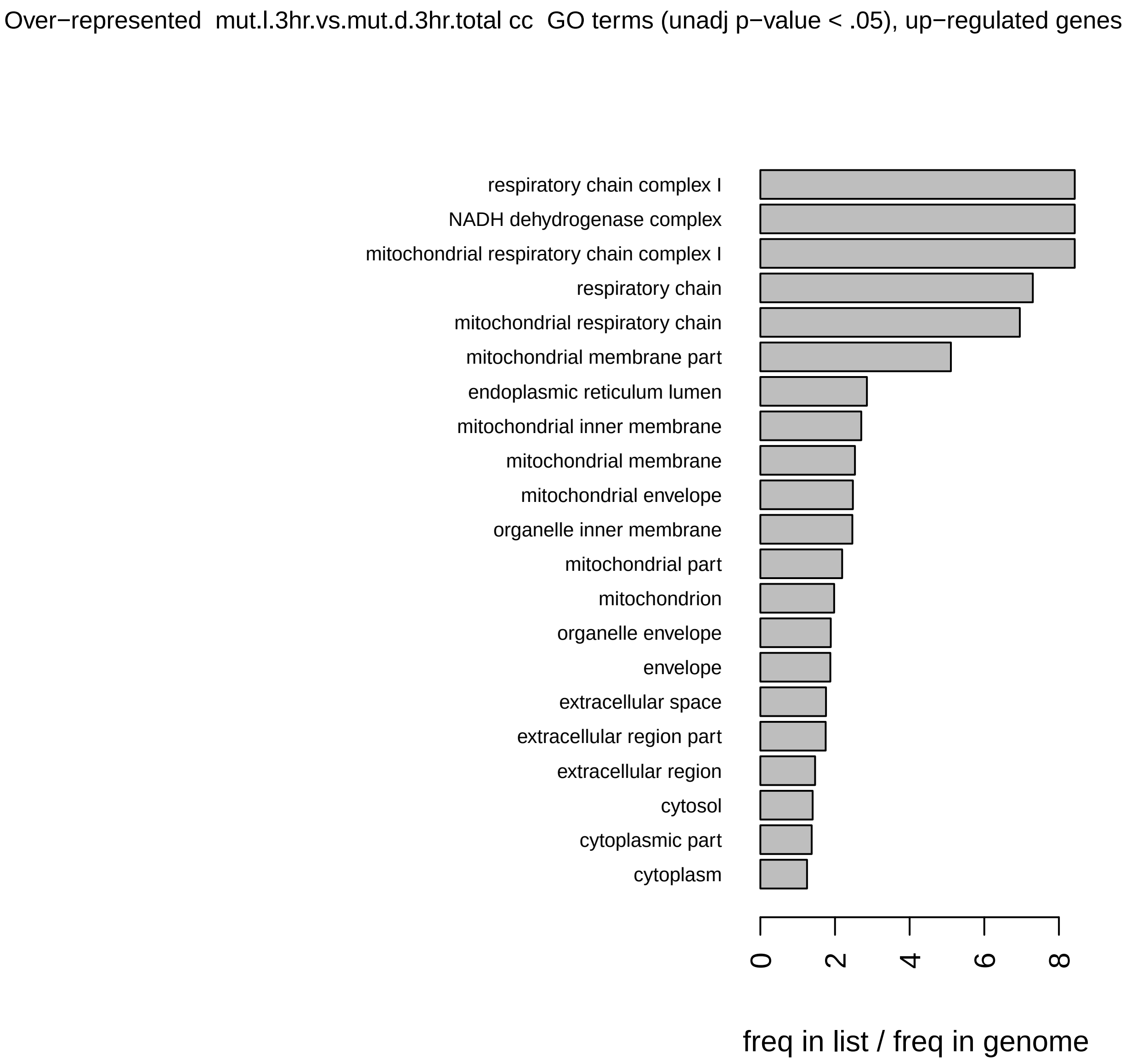
Over−represented mut.l.3hr.vs.mut.d.3hr.total cc GO terms (unadj p−value < .05), up−regulated genes
respiratory chain complex I
NADH dehydrogenase complex
mitochondrial respiratory chain complex I
respiratory chain
mitochondrial respiratory chain
mitochondrial membrane part
endoplasmic reticulum lumen
mitochondrial inner membrane
mitochondrial membrane
mitochondrial envelope
organelle inner membrane
mitochondrial part
mitochondrion
organelle envelope
envelope
extracellular space
extracellular region part
extracellular region
cytosol
cytoplasmic part
cytoplasm
0
2
4
6
8
freq in list / freq in genome
