# Supplementary material for: Improved transcription and translation with L-leucine stimulation of mTORC1 in Roberts syndrome
Source: BMC Genomics. 2016 Jan 5;17:25. doi: 10.1186/s12864-015-2354-y (PMC4700579; doi:10.1186/s12864-015-2354-y)
Supplement: Additional file 2: Table S1. — Summary table of Babel analysis. Table S2. GO term analysis of genes that show improved translational efficiency in RBS cells with L-leucine. Table S3. List for genes with fold change in translational efficiency greater than two and a total number of reads over 20 counts at 3 h. Table S4. List for genes with fold change in translational efficiency greater than two and a total number of reads over 20 counts at 24 h. Table S5. GO term analysis for genes with the new motif. Table S6. List of genes that have the new motif. Table S7. Summary table of data in boxplot of mTORC2-regulated gene show transcription and translation in Figure S5. Table S8. Summary table of data in heatmap for 868 mitochondrial genes—expression (Fig. 3) and translation. Table S9. GO term analysis for mitochondrial genes that show improve transcription with L-leucine. Table S10. GO term analysis for mitochondrial genes that improve translation with L-leucine. Table S11. Summary table of data in boxplot of snoRNAs expression in Fig. 4. Table S12. GO term analysis of differentially expressed genes in RBS cells. Table S13. List of genes in NADs and their expression, from Fig. 6. (ZIP 1.78 MB) [file 12864_2015_2354_MOESM2_ESM.zip › Table S10-go_barplots_gomito_transeff.pdf]

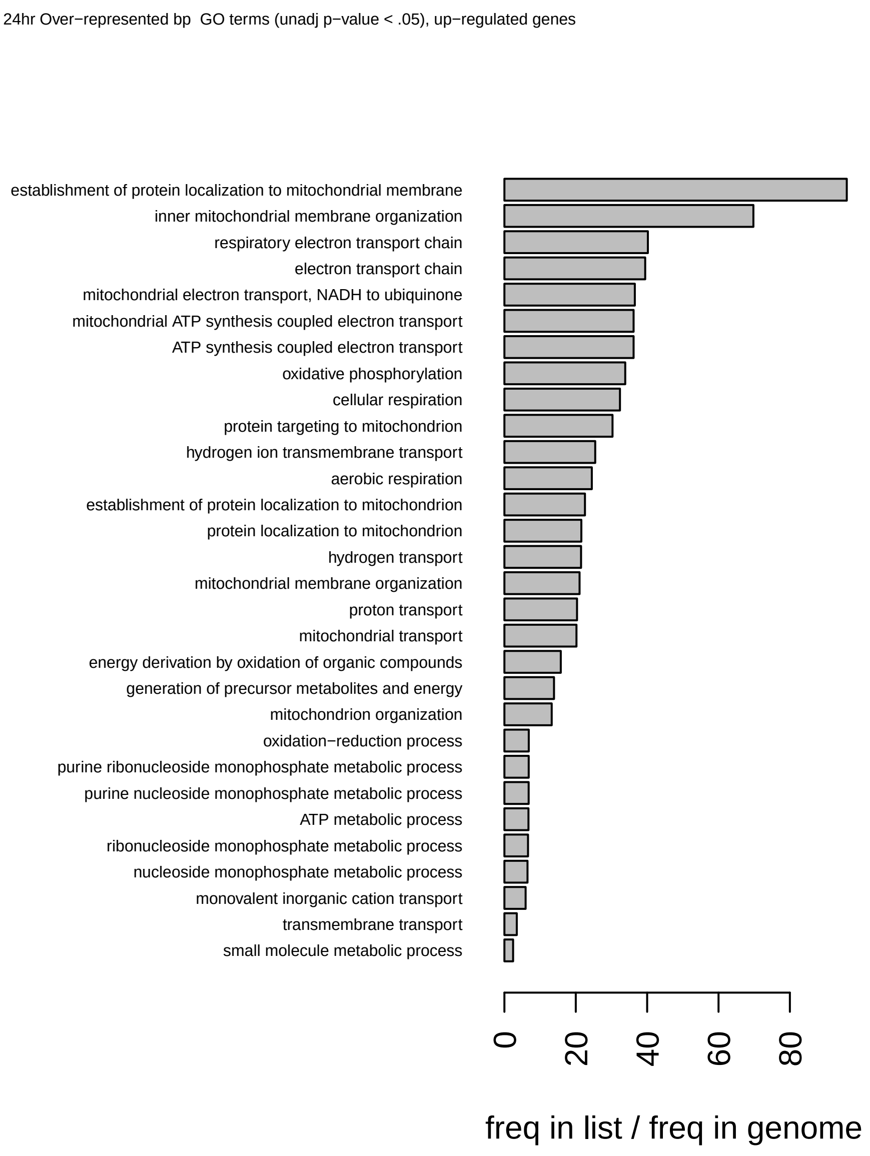

24hr Over−represented bp GO terms (unadj p−value < .05), up−regulated genes
establishment of protein localization to mitochondrial membrane
inner mitochondrial membrane organization
respiratory electron transport chain
electron transport chain
mitochondrial electron transport, NADH to ubiquinone
mitochondrial ATP synthesis coupled electron transport
ATP synthesis coupled electron transport
oxidative phosphorylation
cellular respiration
protein targeting to mitochondrion
hydrogen ion transmembrane transport
aerobic respiration
establishment of protein localization to mitochondrion
protein localization to mitochondrion
hydrogen transport
mitochondrial membrane organization
proton transport
mitochondrial transport
energy derivation by oxidation of organic compounds
generation of precursor metabolites and energy
mitochondrion organization
oxidation−reduction process
purine ribonucleoside monophosphate metabolic process
purine nucleoside monophosphate metabolic process
ATP metabolic process
ribonucleoside monophosphate metabolic process
nucleoside monophosphate metabolic process
monovalent inorganic cation transport
transmembrane transport
small molecule metabolic process
0
20
40
60
80
freq in list / freq in genome

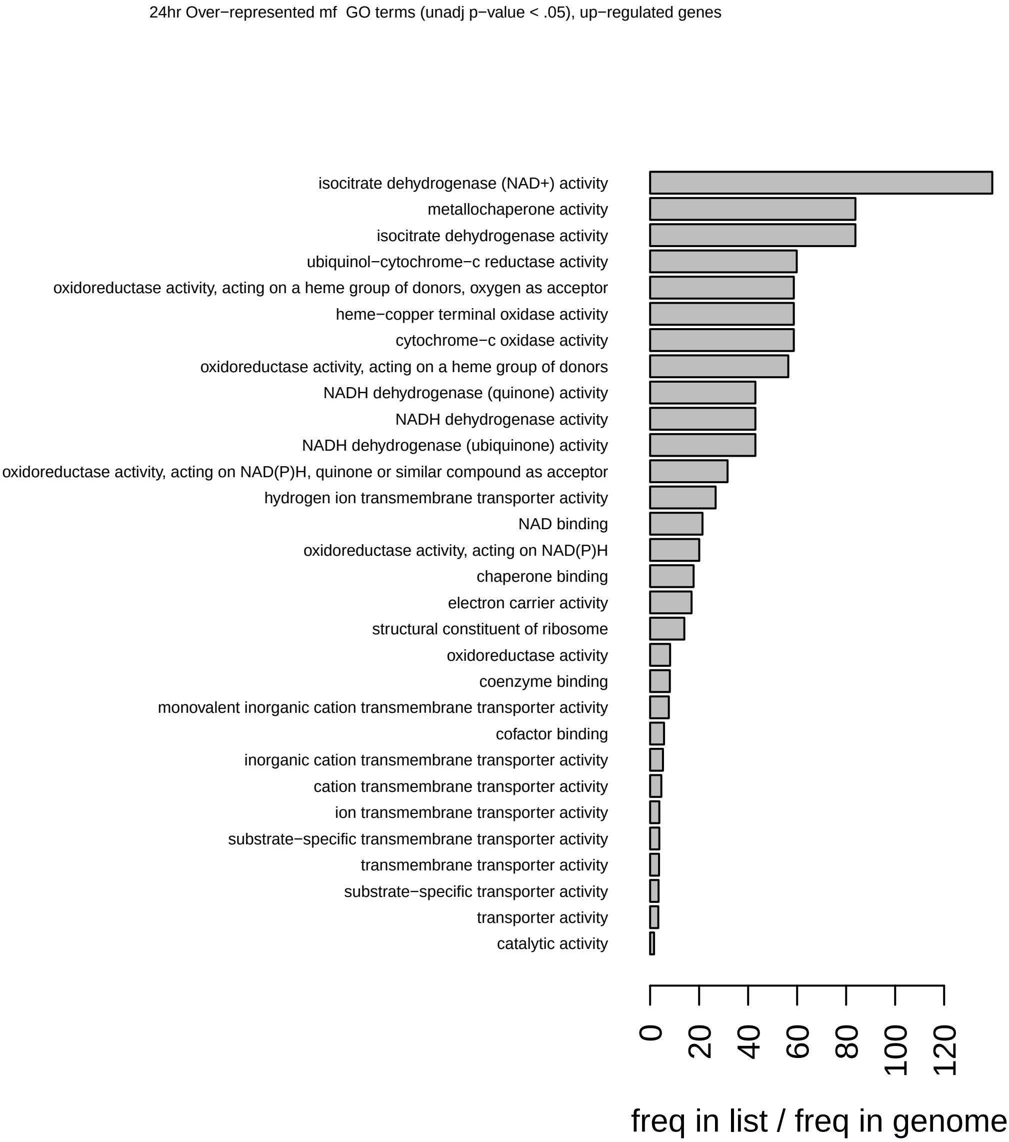
24hr Over−represented mf GO terms (unadj p−value < .05), up−regulated genes
isocitrate dehydrogenase (NAD+) activity
metallochaperone activity
isocitrate dehydrogenase activity
ubiquinol−cytochrome−c reductase activity
oxidoreductase activity, acting on a heme group of donors, oxygen as acceptor
heme−copper terminal oxidase activity
cytochrome−c oxidase activity
oxidoreductase activity, acting on a heme group of donors
NADH dehydrogenase (quinone) activity
NADH dehydrogenase activity
NADH dehydrogenase (ubiquinone) activity
oxidoreductase activity, acting on NAD(P)H, quinone or similar compound as acceptor
hydrogen ion transmembrane transporter activity
NAD binding
oxidoreductase activity, acting on NAD(P)H
chaperone binding
electron carrier activity
structural constituent of ribosome
oxidoreductase activity
coenzyme binding
monovalent inorganic cation transmembrane transporter activity
cofactor binding
inorganic cation transmembrane transporter activity
cation transmembrane transporter activity
ion transmembrane transporter activity
substrate−specific transmembrane transporter activity
transmembrane transporter activity
substrate−specific transporter activity
transporter activity
catalytic activity
0
20
40
60
80
100
120
freq in list / freq in genome

24hr Over−represented cc GO terms (unadj p−value < .05), up−regulated genes

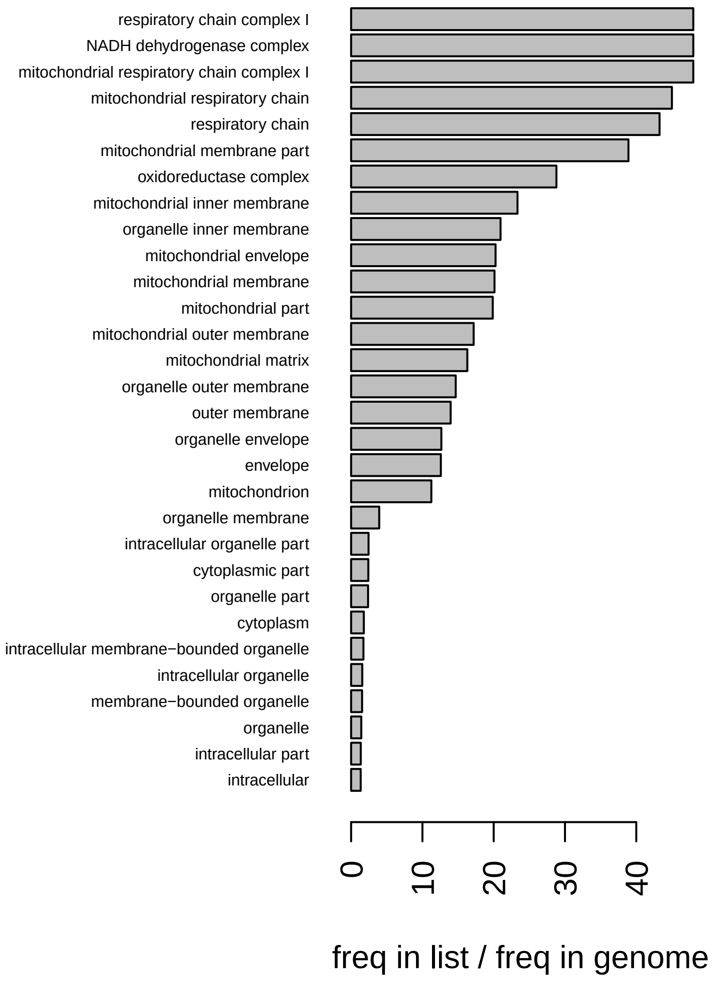

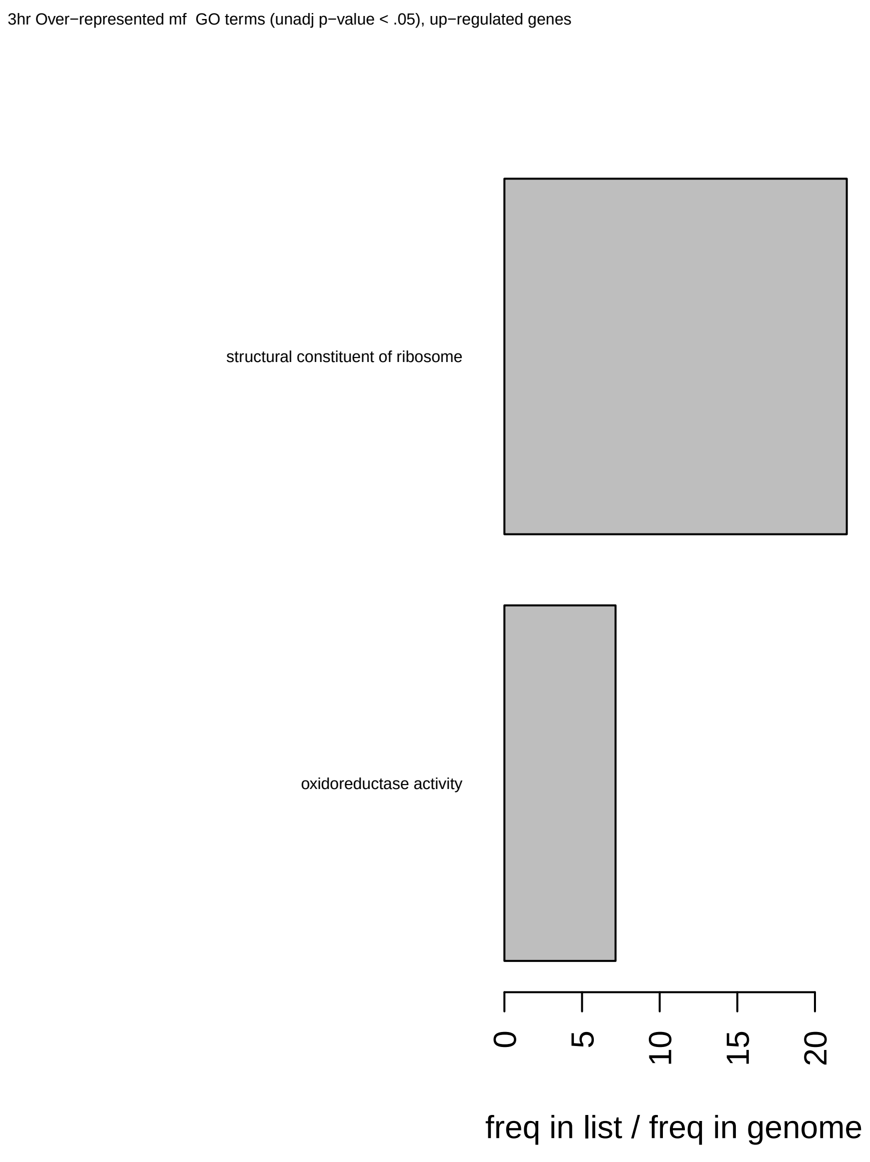

3hr Over−represented mf GO terms (unadj p−value < .05), up−regulated genes
structural constituent of ribosome
oxidoreductase activity
0
5
10
15
20
freq in list / freq in genome

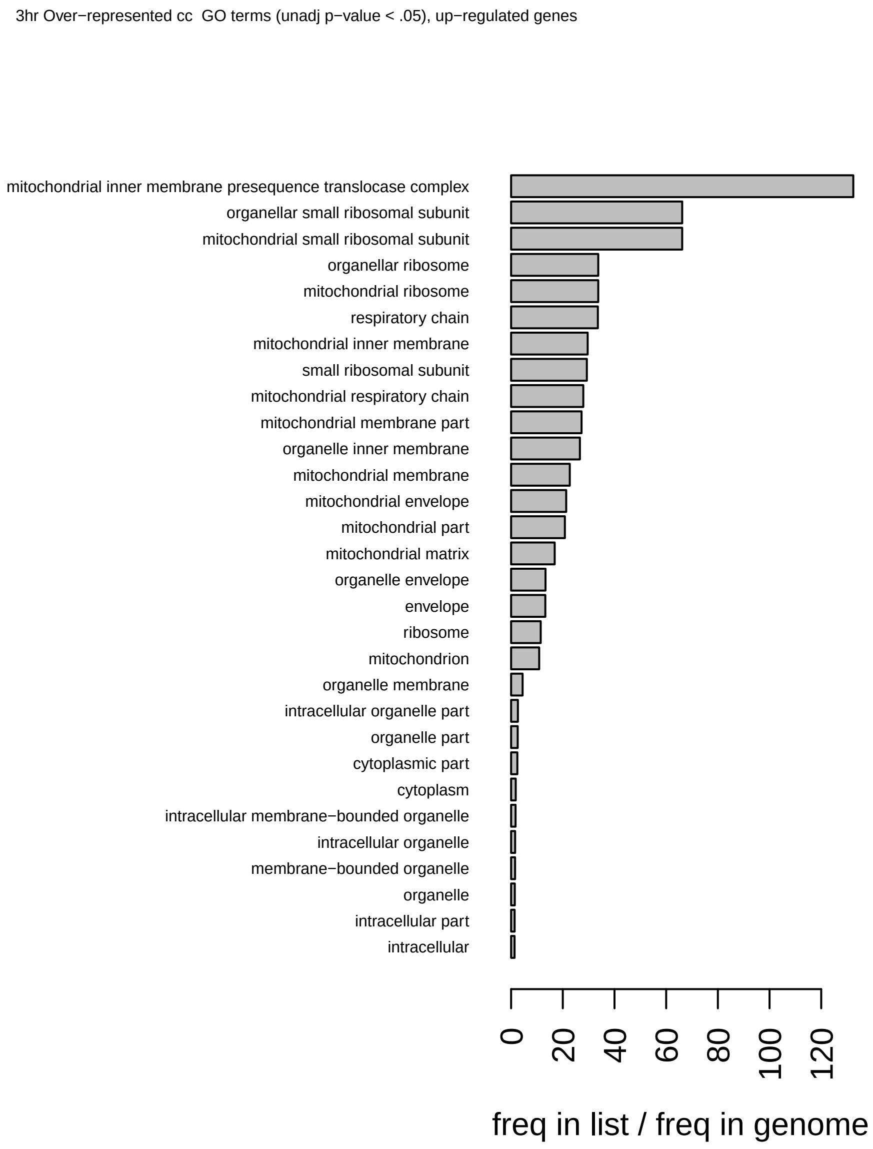
3hr Over−represented cc GO terms (unadj p−value < .05), up−regulated genes
mitochondrial inner membrane presequence translocase complex
organellar small ribosomal subunit
mitochondrial small ribosomal subunit
organellar ribosome
mitochondrial ribosome
respiratory chain
mitochondrial inner membrane
small ribosomal subunit
mitochondrial respiratory chain
mitochondrial membrane part
organelle inner membrane
mitochondrial membrane
mitochondrial envelope
mitochondrial part
mitochondrial matrix
organelle envelope
envelope
ribosome
mitochondrion
organelle membrane
intracellular organelle part
organelle part
cytoplasmic part
cytoplasm
intracellular membrane−bounded organelle
intracellular organelle
membrane−bounded organelle
organelle
intracellular part
intracellular
0
20
40
60
80
100
120
freq in list / freq in genome
